# Discovery of Niclosamide Analogs with Potent Mitochondrial Uncoupling Activity and Reduced Mitochondrial Inhibition–Associated Toxicity

**DOI:** 10.1021/acsmedchemlett.5c00439

**Published:** 2026-02-09

**Authors:** Haowen Jiang, Alessio Macorano, Enming Xing, Mohamed Jedoui, Shabber Mohammed, Vanessa Lee, Jeffrey Cheng, Lain McDonough, Xiaolin Cheng, Jiangbin Ye, Pui Kai Li

**Affiliations:** † Department of Radiation Oncology, 583939 Stanford University School of Medicine, Stanford, CA 94305; ‡ Division of Medicinal Chemistry and Pharmacognosy, College of Pharmacy, 15480 The Ohio State University, Columbus Ohio 43210, United States

**Keywords:** Niclosamide, Mitochondrial uncoupler, Structure−activity relationships, Warburg effect

## Abstract

Niclosamide, an FDA-approved anthelmintic, functions as a mitochondrial uncoupler with promising anticancer potential, yet its efficacy remains limited, often ascribed to poor bioavailability. We identify a more fundamental constraint—its narrow therapeutic window arising from a biphasic mechanism that promotes uncoupling at low doses but inhibits respiration at higher doses. To overcome this limitation, we synthesized 30 niclosamide analogs, systematically profiled their mitochondrial responses using Seahorse MitoTox assay, and developed QSAR models to uncover structural determinants of efficacy and toxicity. Niclosamide exhibited a narrow uncoupling range (0.5–1 μM) beyond which respiration was suppressed. Several analogs, including Nic-2, Nic-8, Nic-40, and Nic-43, sustained uncoupling for up to 9 h at concentrations up to 10 μM, with some showing improved signal modulation and reduced cytotoxicity. QSAR analysis revealed that substitution electronic properties and ring-specific hydrophobicity are related to the therapeutic index. These findings expand niclosamide’s therapeutic window through rational scaffold tuning, enabling safer mitochondrial reprogramming strategies for cancer therapy.

## Graphical abstract

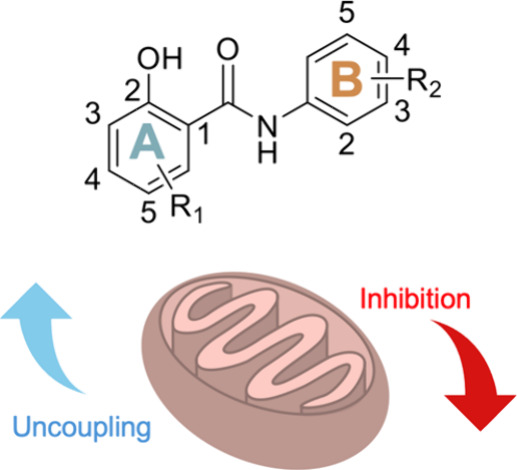

Mitochondrial uncoupling occurs naturally in various physiological contexts, such as thermogenesis, and is mediated by endogenous uncoupling proteins (UCPs), fatty acids, and hormones.^1^ Mitochondrial uncouplers (MUs) are compounds that induce mitochondrial uncoupling by dissipating the proton gradient generated by the electron transport chain (ETC), which normally drives ATP synthesis via ATP synthase.^2–5^ By dissipating the proton gradient, uncouplers eliminate the thermodynamic constraint on electron transport, enabling the ETC to operate at maximal rates as mitochondria strive to restore the gradient. This elevated electron flux drives increased oxygen consumption, as oxygen serves as the terminal electron acceptor.

Synthetic small-molecule mitochondrial uncouplers have emerged as promising therapeutics for obesity, metabolic syndrome, and aging-related disorders, with growing interest in their potential for cancer treatment.^2,6–10^ Among these, niclosamide (Nic)—an antiparasitic drug and mild mitochondrial uncoupler—has garnered significant attention for its potential when repurposed for cancer therapy.^10–18^ While Nic was initially studied for its inhibition of oncogenic pathways (e.g., Wnt/β-catenin, mTOR, STAT3),^11,12,19^ emerging evidence suggests that mitochondrial uncoupling could represent its primary anticancer effector mechanism, potentially driving metabolic and epigenetic remodeling in tumors. For instance, NEN (Nic’s ethanolamine salt) stimulates ETC activity, elevating NAD^+^/NADH to increase pyruvate/lactate (reversing Warburg effect) and α-KG/2-HG ratios^20,21^ while suppressing reductive carboxylation.^22^ These metabolic reprograming drive epigenetic remodeling via CpG-island demethylation. In neuroblastoma model, these changes promote neuronal differentiation, silence oncogenes (MYCN, β-catenin), and activate tumor suppressors (e.g., p53) while inhibiting HIF signaling.^20^

While suboptimal systemic bioavailability of Nic (plasma concentrations: 0.1–0.72 μM)^12,23,24^ has been widely implicated as its primary limitation, however, this rationale fails to explain its limited clinical adoption in colorectal cancer (NCT02687009 and NCT02519582), where the drug directly interacts with intestinal tumors at high local concentrations unconstrained by systemic absorption. This paradox highlights unresolved resistance mechanisms independent of pharmacokinetics, necessitating the development of novel Nic analogs that retain its mitochondrial uncoupling activity while overcoming this unknown limitation.

The uncoupling activity of Nic and its structural analogs involves shuttling protons across the mitochondrial membrane (Figure 1A). Upon deprotonation of the phenolic OH, an intramolecular six-membered hydrogen-bonded ring forms between that oxygen and the aniline nitrogen.^25^ This H-bonded ring delocalizes the negative charge, therefore stabilizing the anionic form of niclosamide and increase hydrophobicity of the salicylanilide scaffold ∼40-fold as indicated by Storey et al.^26^ Such weak acid with a hydrophobic scaffold has the intrinsic ability to carry protons across membrane as both anion A^–^ and neutral form HA can be absorbed in the membrane solution interface. Such capability is closely related to modifications on the salicylanilide core scaffold, which influences the dissociation ability of the phenolic hydroxyl group. Side chain substituents on the scaffold need to be electron-withdrawing to maintain activity,^17,19^ whereas the absence of electron-withdrawing groups or the introduction of electron-donating groups eliminates activity. At concentrations above its uncoupling threshold, niclosamide may inhibit mitochondrial respiration through nonuncoupling mechanisms, similar to FCCP. This effect likely reflects impaired substrate-supported respiration, potentially due to interference with mitochondrial substrate transport across the inner membrane, as direct inhibition of the electron transport chain has been ruled out.^27^ This high-dose inhibition critically depends on the strong electron-withdrawing 4-nitro substituent on B-ring^13,27^ (Figure 1A), whose removal abolishes oxygen consumption blockade.^27^ Moreover, excessive proton shuttling can trigger opening of the mitochondrial permeability transition pore (mPTP), leading to membrane potential collapse and matrix swelling.^7^ We hypothesize that niclosamide exerts a dose-dependent biphasic effect on mitochondrial function, where stimulation at low concentrations and inhibition at higher doses together result in a narrow therapeutic window that limits its clinical applicability. Therefore, optimizing the therapeutic window of niclosamide for anticancer applications requires balancing its mitochondrial uncoupling efficacy with the onset of direct respiratory inhibition at higher doses.

**Figure 1:**
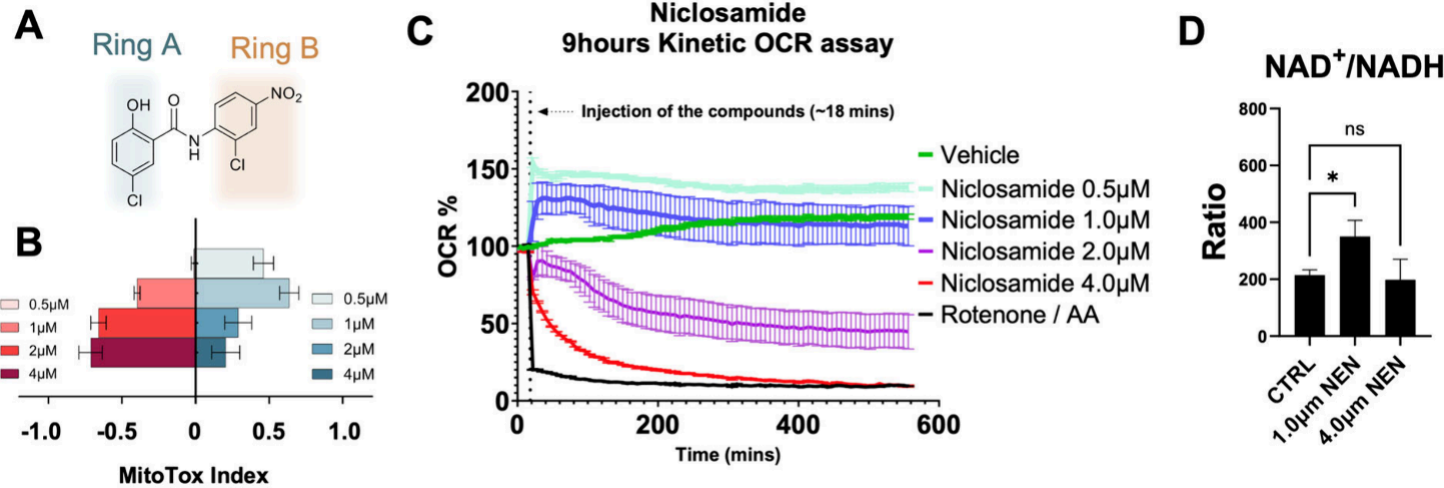
MitoTox assay and extended kinetic analysis illustrating the uncoupling effects and oxygen consumption ratio (OCR) of niclosamide. (A) the structure of niclosamide, with labeled rings for easy reference. (B) MitoTox profile of niclosamide. Blue bars represent the uncoupling activity, while the red bars indicate toxicity, as measured by a standard MitoTox assay (*n* = 3). (C) OCR assessed using a 9 h Seahorse assay. Values exceeding 100% suggest sustained uncoupling activity (*n* = 3), whereas OCR values below 100% signal inhibition of respiration. (D) Intracellular NAD^+^/NADH ratios in NB16 cells treated with niclosamide for 5 h, as measured by LC–MS (*n* = 3). Data represent mean ± SD; *P < 0.05.

## Dose-Dependent Biphasic Effect of Niclosamide on Mitochondrial Respiration

To validate our hypothesis, we systematically investigated the dose-dependent effects of Nic on mitochondrial status determined by MitoTox assay. There is an increase in the uncoupling activity as the concentration of Nic increased from 0.5 to 1 μM. However, the uncoupling activity decreases as the concentration increases 2 μM and 4 μM. In addition, there is a corresponding increase in mitochondria inhibition from 2 μM to 4 μM (Figure 1B). The longitudinal OCR analysis revealed distinct concentration-dependent trajectories: sustained protonophoric uncoupling persisted throughout the 9 h assay at 0.5 μM (evidenced by maintained OCR elevation ∼130% baseline), whereas 1 μM elicited transient uncoupling limited to the initial 5 h phase (OCR peak at 200 min followed by progressive decline to ∼95% baseline). Notably, higher concentrations (2–4 μM) induced rapid mitochondrial suppression, with 4 μM mirroring the complete respiratory inhibition observed in Rotenone/Antimycin A controls (OCR collapse to ∼5% baseline by 400 min), indicative of electron transport chain blockade (Figure 1C). These data collectively establish a narrow therapeutic window (0.5–1 μM) for Nic to exert uncoupling activity without triggering mitochondrial respiration failure, beyond which mitochondrial inhibition becomes the dominant phenotype. Intracellular NAD^+^/NADH ratios measured by LC–MS after 5 h Nic treatment showed a transient increase at 1 μM, which was not observed at 4 μM (Figure 1D), suggesting that changes in cellular redox state parallel the biphasic mitochondrial response.

## Synthesis and Evaluation of the Next Generation of Niclosamide Analogs

To overcome the narrow therapeutic window of Nic and mechanistically dissect how A/B-ring substitutions modulate the uncoupling-inhibition dichotomy, we rationally designed 30 analogs through fine-tuning phenolic hydroxyl (A-ring) and chlorophenyl (B-ring) modifications, followed by systematic therapeutic index quantification via MitoTox profiling (Figure 2). The MitoTox values are derived from oxygen consumption rate (OCR) measurements following sequential oligomycin and BAM15 injections, where positive values (0 to 1) indicate uncoupling and negative values (0 to −1) indicate inhibition.^28^ The salicylanilide scaffold—the structural core of Nic—functions as a weakly acidic, lipophilic protonophore, shuttling protons across the inner mitochondrial membrane to uncouple oxidative phosphorylation and stimulate ETC activity while dissipating the membrane potential. The *o*-hydroxy group can form an intramolecular hydrogen bond in both its protonated and deprotonated states; hence, fine-tuning the electronic properties of the A- and B-rings offers a route to modulate the hydroxy p*K*_a_, proton-binding affinity, and membrane permeability. Accordingly, we synthesized three analogs that preserve the salicylanilide core but vary substitution patterns on both phenyl rings to systematically investigate how these modifications reshape the electronic landscape and biological activity. All niclosamide analogs were synthesized via a one-step amide coupling reaction between the corresponding anilines and the substituted benzoic acids (detailed synthesis procedures can be found in the Supporting Information). The analogs demonstrated a striking divergence in protonophoric efficacy and mitochondrial safety profiles, establishing a critical structure-dependent dissociation between uncoupling activity and cytotoxic liability.

**Figure 2:**
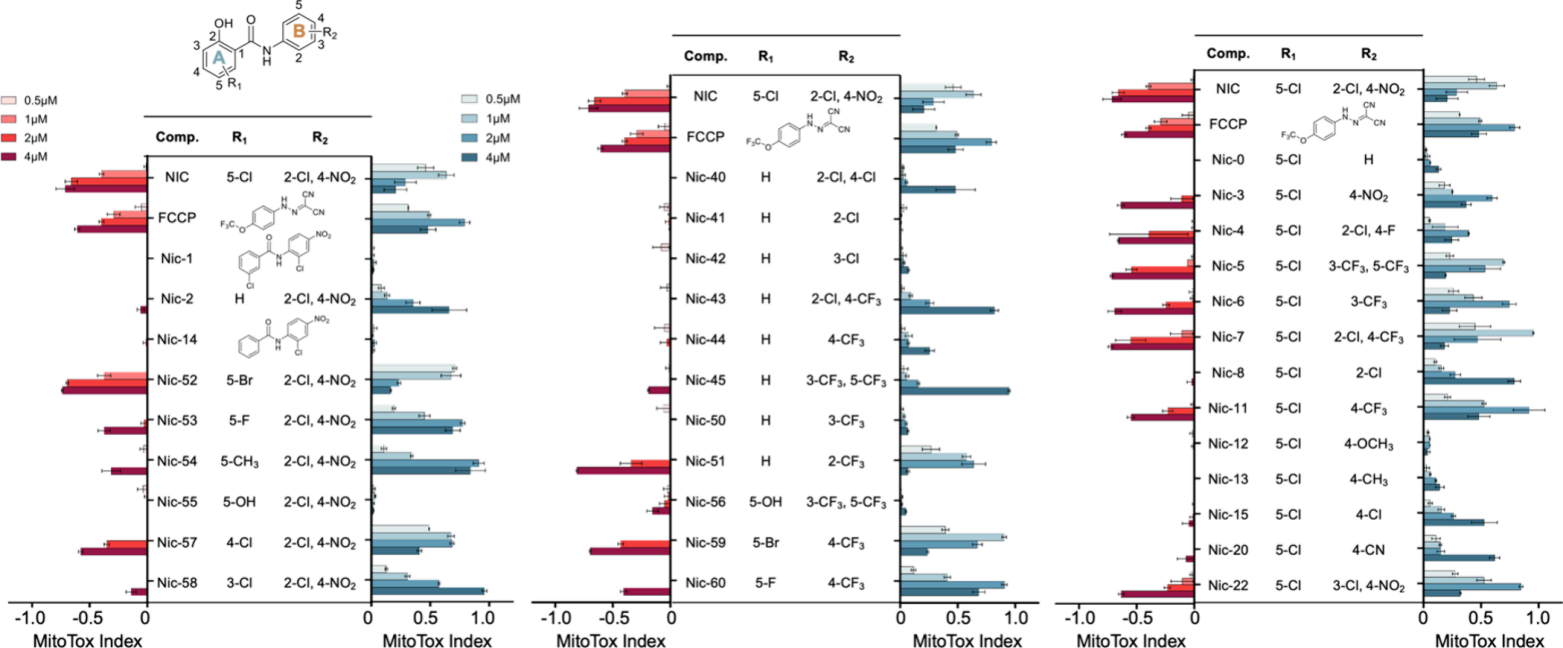
MitoTox profiles of synthesized niclosamide analogs. Blue bars indicate uncoupling activity, while red bars represent inhibitory toxicity, as measured by a standard MitoTox assay (*n* = 3). (left) Variations only in the A-ring substituents. (right) Variations only in the B-ring substituents. (middle) Variations in substituents of both rings.

Initial observations established a fundamental prerequisite: analogs devoid of mitochondrial uncoupling activity (e.g., Nic-1, Nic-12, Nic-14, Nic-41, Nic-42, Nic-50, Nic-55, Nic-56) consistently showed no mitochondrial toxicity, confirming the functional linkage between uncoupling activity and inhibitory potential. Significantly, this inhibition dependence manifested clear structural specificity, as evidenced by the divergent safety profiles of Nic-52, Nic-4–6 (high inhibition) versus Nic-2, Nic-8, Nic-40, Nic-43 (low inhibition) despite comparable uncoupling capacities.

Delving into structural determinants, we first dissected A-ring pharmacophores through systematic deconstruction. Removal of the phenolic hydroxyl group (Nic-1, Nic-14) abolished detectable uncoupling activity within the tested concentration range (0.25–4 μM), whereas chlorine substitution (Nic-2 vs parent Nic) proved nonessential. This established the hydroxyl group as the critical hydrogen-bonding motif for proton shuttle formation. Parallel A-ring investigations (Nic-53–58 series) revealed an electronic requirement: only electron-withdrawing substituents sustained uncoupling activity. Halogen positioning and electronic character modulated efficacy gradients, with electron-donating groups (Nic-12/13 methyl/methoxy) completely suppressing mitochondrial uncoupling function.

Therapeutic optimization emerged through combinatorial ring engineering. Maintaining the essential A-ring hydroxyl while tuning B-ring electronics allowed precise control over uncoupling intensity—a parametric relationship validated across hybrid analogs (Figure 2, middle panel). Notably, inhibition divergence within active analogs suggested secondary structural determinants beyond basic electronic requirements, possibly involving steric interactions with mitochondrial membranes or off-target binding.

## Dynamic Oxygen Consumption Profiling of Novel Nic Analogs Across Extended Time-Course and Concentration Gradients

To evaluate whether structural modifications to niclosamide improved mitochondrial selectivity and uncoupling durability, we performed extended oxygen consumption profiling on four lead analogs—Nic-2, Nic-8, Nic-40, and Nic-43—across a 9 h Seahorse assay (Figure 3A and Supplementary Figure 5). All four compounds demonstrated sustained OCR elevation throughout the entire time course, in contrast to the biphasic trajectory of niclosamide, which exhibits transient uncoupling followed by delayed mitochondrial inhibition. We observed that these niclosamide analogs exhibited a similar profile as industrial standard BAM15, and produced a more sustained elevation in OCR compared with Nic or FCCP (Supplementary Figures 3 and 4).^8,29^ Notably, Nic-43 and Nic-8 maintained OCR levels exceeding 120% of baseline during the final 100 min, reflecting durable protonophoric activity with minimal late-phase toxicity. Quantitative analysis of average OCR between 400–500 min further confirmed that these analogs preserved respiratory activity significantly better than niclosamide (Figure 3B). These enhancements are linked to strategic A- and B-ring modifications that maintain the critical A-ring hydroxyl while fine-tuning B-ring electronics to optimize uncoupling efficacy and reduce inhibition. To further assess the downstream signaling effects of these mitochondrial modulators, we examined β-catenin, p53, and mTORC1 pathway activity following 24 h treatment of NB16 cells with niclosamide and its analogs. Among the four candidates, Nic-8 and Nic-43 showed a more favorable signaling profile, effectively repressing β-catenin expression and inhibiting mTORC1 signaling even at 1 μM. In contrast, Nic-2 and Nic-40 required higher concentrations (4 μM) to achieve comparable inhibition. Notably, all analogs robustly activated p53, but Nic-8 and Nic-43 induced the strongest response at both concentrations (Figure 3C). Consistent with their reduced mitochondrial toxicity, these analogs also exhibited markedly lower acute cytotoxicity than niclosamide, maintaining significantly higher cell viability across a broad dose range (Figure 3D). Collectively, these findings highlight the capacity of rational design to decouple uncoupling activity from respiratory suppression and expand the therapeutic window of mitochondrial uncouplers.

**Figure 3:**
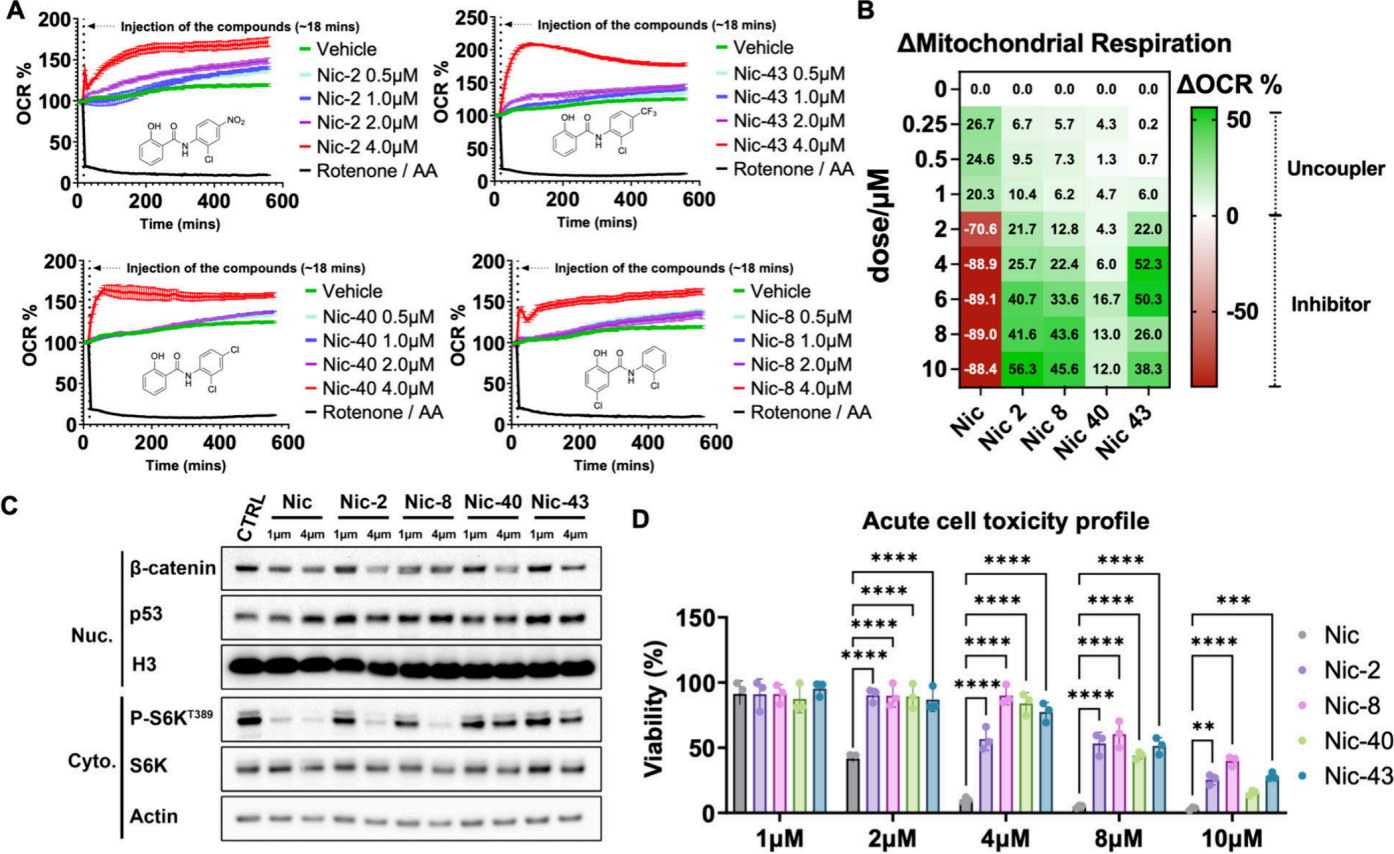
Dynamic oxygen consumption profiling of novel Nic analogs. (A) Nine-hour oxygen consumption kinetics of promising compounds with optimal therapeutic windows: Nic-2, Nic-8, Nic-40, and Nic-43 (*n* = 3). (B) Average oxygen consumption (represented by mitochondria respiration difference compared to no treatment) over a 400–500 min window indicates that these compounds demonstrate significantly enhanced uncoupling effects compared to niclosamide, while exhibiting minimal toxicity within the tested concentration range (*n* = 3). (C) NB16 cells were treated by different concentrations of Nic for 24 h, and the cytosolic and nuclear parts were analyzed by Western blot for the indicated proteins. Representative results are shown. (D) NB16 cells were treated with various concentrations of Nic and its analogs for 24 h, and cell viability was assessed using trypan blue staining (*n* = 3).

## QSAR Modeling of Inhibitory and Uncoupling Activities of Niclosamide Analogs

To understand how aromatic substituents on the salicylanilide core influence mitochondrial uncoupling versus respiratory inhibition, we developed quantitative structure–activity relationship (QSAR) models correlating electronic and steric descriptors with MitoTox assay results using Support Vector Regression (SVR). Model performance was concentration-dependent, with the 2 μM data set yielding optimal predictive capacity (*Q* ^2^ = 0.594 for uncoupling; *Q* ^2^ = 0.746 for respiratory inhibition under leave-one-out cross-validation; Figure 4A,B,E). Feature importance analysis (Figure 4C,D,F,G) revealed that A-ring hydrophobicity (∑π_A) predominantly influenced uncoupling activity, while the Hammett σ constants at the para and ortho positions (R1–5_σ_p_, R1–3_σ_o_-phenol; see Figure 5 for annotation) were more critical for respiratory inhibition. Anion stabilization energy (De_TOTAL) contributed comparably to both activities. These findings suggest that enhanced uncoupling may be achieved by retaining a weakly acidic, membrane-permeable protonophore with adequate A-ring hydrophobicity and mild electron-withdrawing groups, while toxicity reduction requires avoiding strong electron-withdrawing groups on the A-ring and large nonpolar surface areas. Detailed QSAR methodology, further discussion, concentration-dependent model evaluation, and applicability domain analyses are provided in .

**Figure 4:**
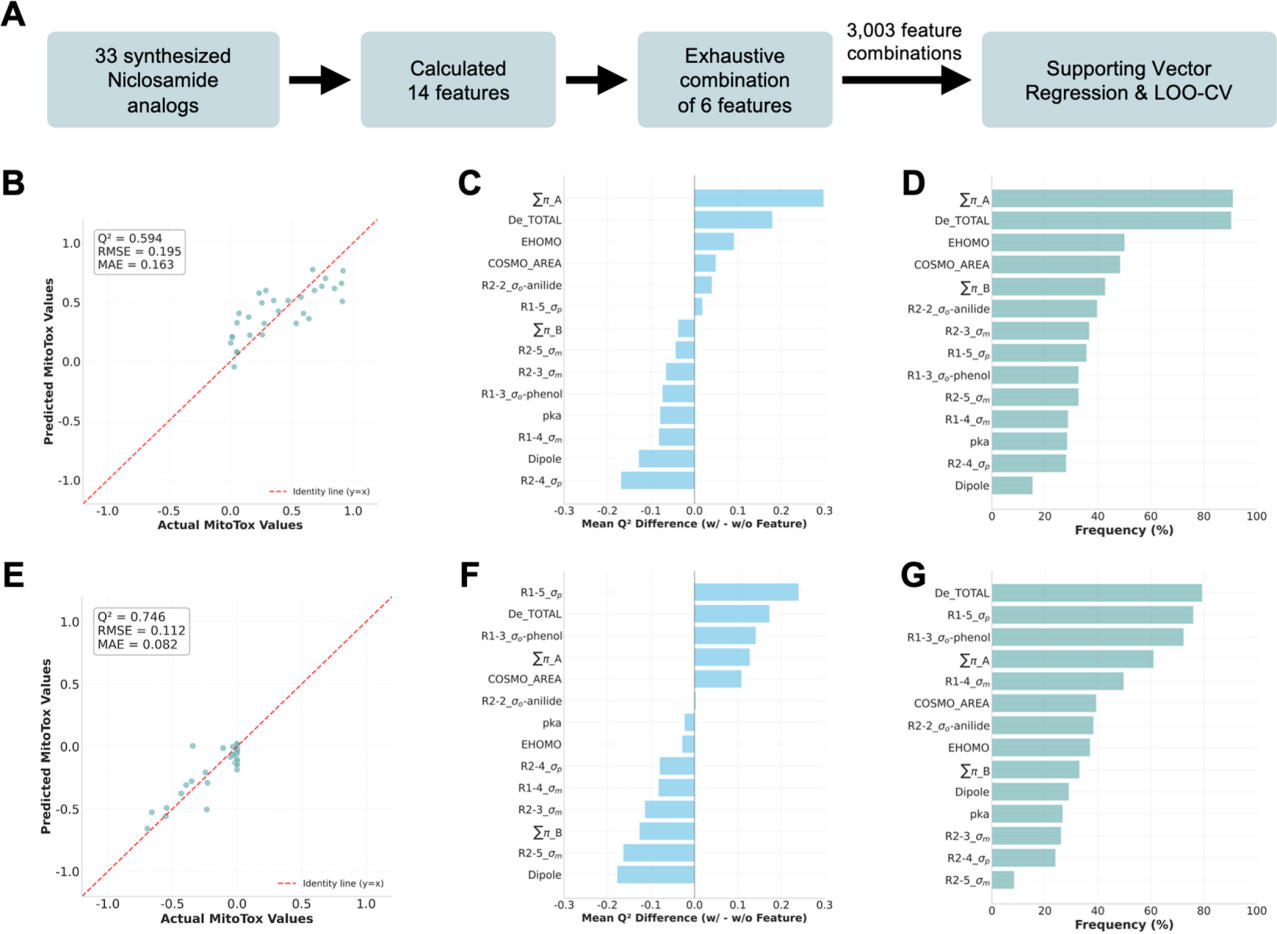
QSAR modeling analysis of niclosamide analogs. (A) QSAR modeling workflow for all evaluated analogs. (B–D) Uncoupling activity analysis at 2 μM: (B) predicted versus actual MitoTox profiling values; (C) feature impact ranking based on mean LOOCV *Q* ^2^ difference; (D) feature occurrence frequency among top 10% performance models. (E–G) Inhibitory activity analysis at 2 μM: (E) predicted versus actual MitoTox profiling values; (F) feature impact ranking; (G) feature occurrence frequency among top 10% performance models.

**Figure 5:**
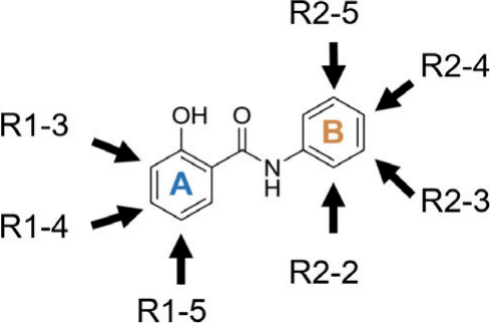
Annotation of positions for assigning substituents’ Hammett inductive parameters.

## Conclusion

Niclosamide (Nic) is widely recognized as a well-established mitochondrial uncoupler, we demonstrated that mitochondrial uncoupling is a promising approach to inhibit the Warburg effect and reprogram metabolism in cancer cells.^12,31–34^ Recently, we also discovered that NEN and retinoic acid (RA) exert a synergistic effect in promoting tumor differentiation both *in vitro* and *in vivo*.^34^ While Nic could be an effective tool for inducing uncoupling, its narrow therapeutic window presents a significant challenge. In this work, we successfully showed that an improved therapeutic window can be established to balance pure uncoupling activity and minimize toxicity induced by Nic. By systematically fine-tuning and modifying different substituents on the Nic core scaffold, we identified Nic-2, Nic-8, Nic-40 and Nic-43 as promising candidates with sustained uncoupling activity and reduced mitochondrial inhibition at high concentrations, which may indicate reduced potential for mitochondrial inhibition–related toxicity compared to niclosamide. In the future, these compounds will be further evaluated in both *in vitro* and *in vivo* cancer models to assess their anticancer activity, pharmacokinetics, and pharmacodynamics. To further elucidate the structure–activity relationship, we also developed a quantitative structure–activity relationship (QSAR) model to decipher the molecular features that contribute to uncoupling activity and inhibition-related toxicity. Our work lays a solid foundation for utilizing mitochondrial uncouplers to reverse abnormal metabolic states in cancer cells. This approach offers the advantage of reprogramming the metabolic network rather than targeting a specific molecular entity, thereby potentially avoiding the rapid development of drug resistance.

## Safety Statement

No unexpected or unusually high safety hazards were encountered.

## Supplementary Material



## Notes

Supplemental figures, materials and methods, and characterization data (PDF)

## §

Key Laboratory of Tropical Translational Medicine of Ministry of Education, International Center for Aging and Cancer, Hainan Medical University, Haikou 571199, ChinaAuthor: Please confirm the city and postal code for Key Laboratory of Tropical Translational Medicine of Ministry of Education, International Center for Aging and Cancer, Hainan Medical University
