# Supplementary material for: Discovery of Niclosamide Analogs with Potent Mitochondrial Uncoupling Activity and Reduced Mitochondrial Inhibition–Associated Toxicity
Source: ACS Med Chem Lett. 2026 Feb 9;17(3):642–8. doi: 10.1021/acsmedchemlett.5c00439 (PMC12989994; doi:10.1021/acsmedchemlett.5c00439)
Supplement: Supplementary file 1 [file ml5c00439_si_001.pdf]

**Supplemental Materials**

**Title: Discovery of Niclosamide Analogs with Potent Mitochondrial Uncoupling Activity and Reduced Mitochondrial Inhibition–Associated Toxicity.**

**Authors:** Haowen Jiang[a,#], Alessio Macorano[b,#], Enming Xing[b,#], Mohamed Jedoui[a,#], Shabber Mohammed[b], Vanessa Lee[a], Jeffrey Cheng[b], Lain McDonough[b], Xiaolin Cheng*, Jiangbin Ye*^, Pui Kai Li*

[a] Department of Radiation Oncology, Stanford University School of Medicine, Stanford, CA 94305.

[b] Division of Medicinal Chemistry and Pharmacognosy, College of Pharmacy, The Ohio State University, Columbus OH, 43210

[#] These authors contributed equally.

* Corresponding Authors: Pui Kai Li (li.27@osu.edu), Jiangbin Ye (yej1@stanford.edu), Xiaolin Cheng (cheng.1302@osu.edu)

^Current Address: Key Laboratory of Tropical Translational Medicine of Ministry of Education, International Center for Aging and Cancer, Hainan Medical University

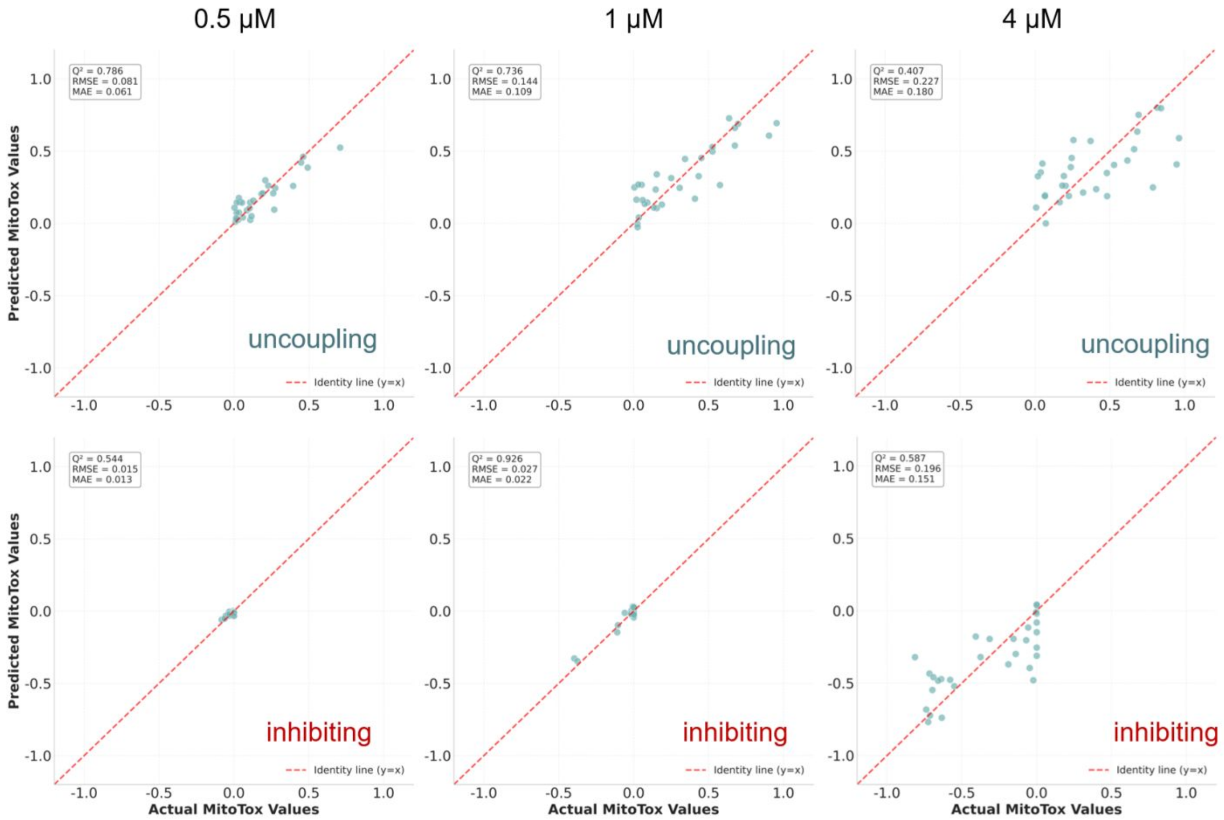


**Supplement Figure 1**. **Actual vs. predicted plots for 0.5 μM, 1 μM, and 4 μM**. An exhaustive combination of 6 out of 14 features for 0.5 μM, 1 μM, and 4 μM with uncoupling and inhibitory activities were performed, and the predicted versus actual MitoTox profiling values from the best model ranked by $Q^2$ scores were plotted. Leave-one-out cross-validation (LOOCV) were performed for each concentration and activity type.

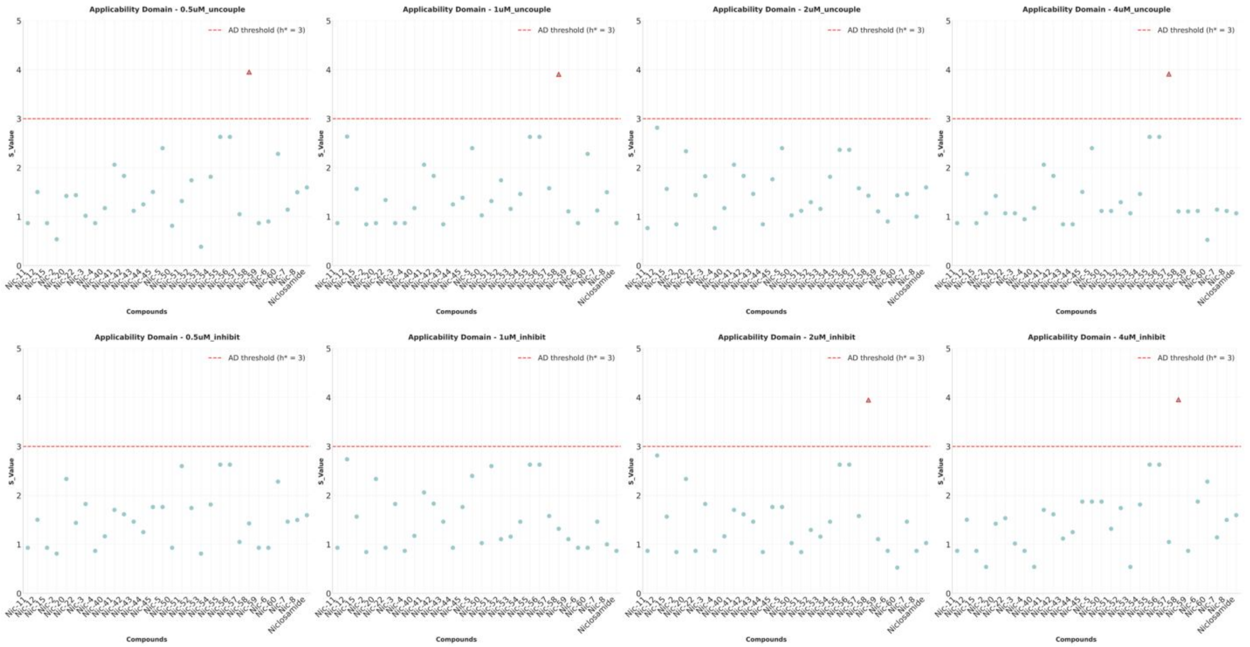

**Supplementary Figure 2. Applicability domain S_value vs. Compound IDs.** The applicability domain of the best models obtained at four concentrations, for both uncoupling activity and inhibitory toxicity, using leave-one-out cross-validation (LOOCV) is shown. For each concentration and activity type, scatter plots of the computed S_value versus compound IDs were presented. AD threshold: Applicability Domain threshold.

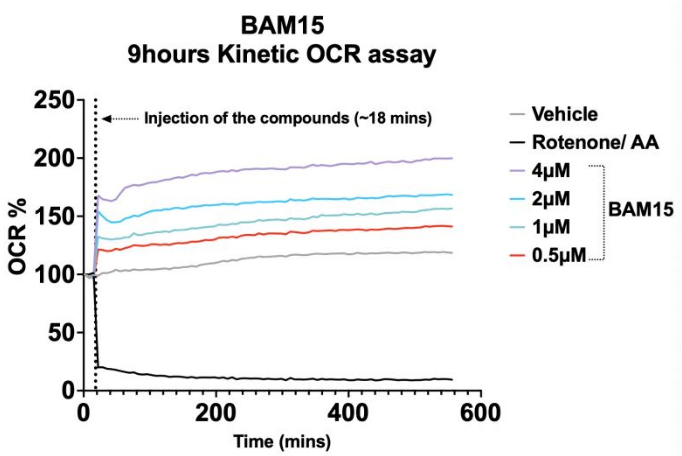


**Supplementary Figure 3. Dynamic Oxygen Consumption Profiling of BAM15.** Nine-hour oxygen consumption kinetics of BAM15 at 0.5μM, 1μM, 2μM, and 4 μM.

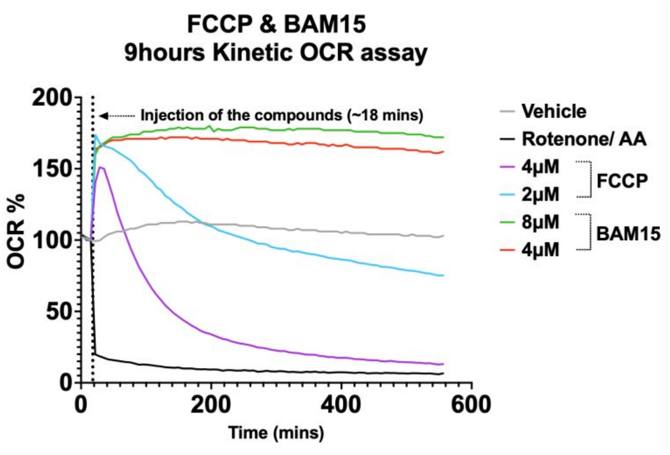


**Supplementary Figure 4. Dynamic Oxygen Consumption Profiling of FCCP and BAM15.** Nine-hour oxygen consumption kinetics of FCCP at 2 μM and 4 μM compared with BAM15 at 4 μM and 8 μM.

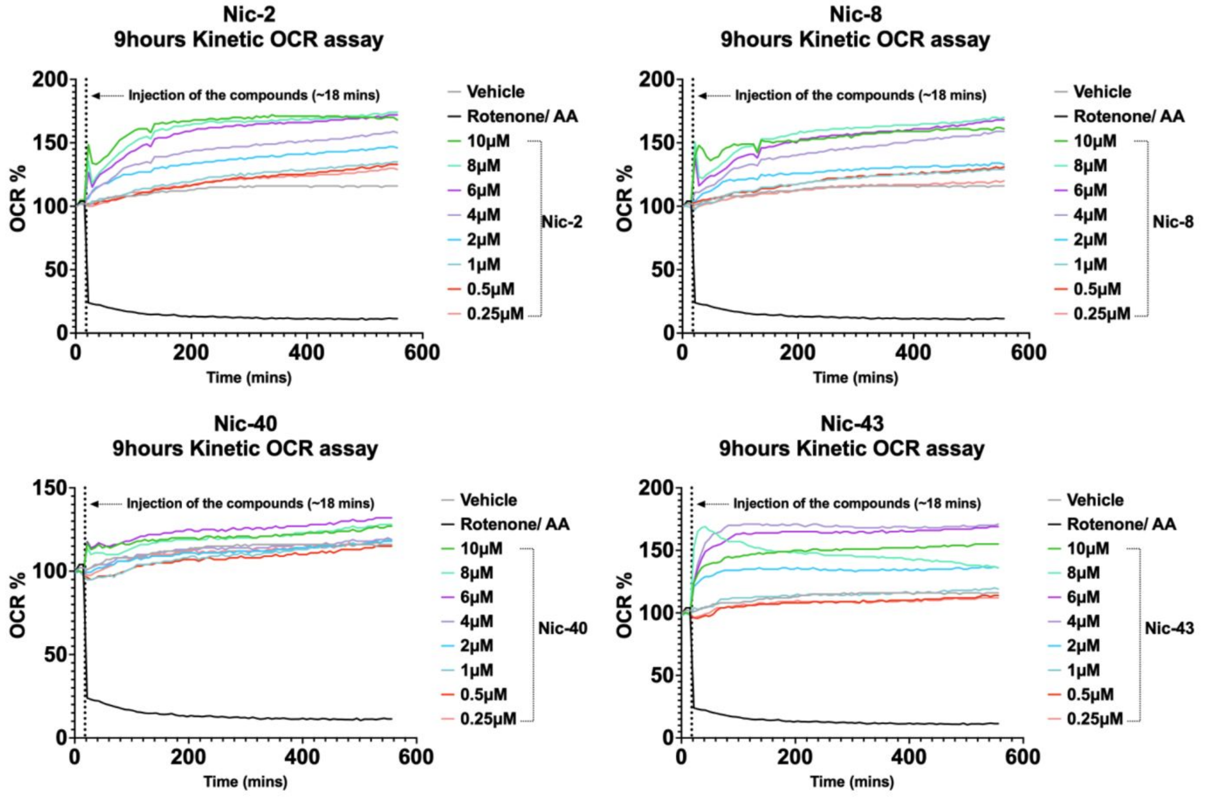


**Supplementary Figure 5. Dynamic Oxygen Consumption Profiling of Novel Nic Analogs.** Nine-hour oxygen consumption kinetics of promising compounds with optimal therapeutic windows (Nic-2, Nic-8, Nic-40, and Nic-43) at the concentration ranging from 0.25 μM to 10 μM.

## Materials and Methods

**Cell Culture**

NB16 neuroblastoma cells were cultured in DMEM/F12 medium (Gibco; 11320033) supplemented with 10% fetal bovine serum (Sigma-Aldrich; F0926), 1% penicillin–streptomycin (Gibco; 15140122), and 1 mmol/L glutamine (Gibco; 25030081). Cells were maintained at 37°C in a humidified incubator with 5% $CO_2$ and were routinely tested for Mycoplasma contamination using the MycoAlert Mycoplasma Detection Kit (Lonza). All experiments were performed using cells between passages 5 and 15.

**Seahorse Assay**

NB16 neuroblastoma cells were used to assess the effects of compounds on mitochondrial function using the Seahorse XF Analyzer. For the acute mitochondrial toxicity (MitoTox) assay, 10,000 NB16 cells were seeded into Seahorse XF96 cell culture microplates, incubated overnight, and then treated with the indicated compounds or vehicle control via port A after baseline OCR measurements. OCR was recorded over a 96-minute period following compound injection. MitoTox assay data were analyzed using the Agilent Seahorse Analytics online platform. For the 9-hour assay, 10,000 NB16 cells were seeded into Seahorse plates, incubated overnight, and then subjected to continuous OCR measurement over 9 hours, during which the indicated compounds or vehicle control were injected through port A at the start of the assay. In both assays, Hoechst 33342 stain (Thermo; #33342) was injected through port D at the end of the run to monitor cell number, although all OCR values were normalized to baseline measurements. The mitochondrial toxicity index (MTI) was calculated using the Agilent Seahorse XF Mito Tox assay, which derives MTI values from oxygen consumption rate (OCR) measurements following sequential oligomycin and BAM15 injections. Positive MTI values (0 to 1) indicate uncoupling, while negative values (0 to –1) indicate inhibition, with vehicle and Rot/AA groups serving as reference controls[1].

**Western Blot**

NB16 cells were treated with different concentrations of Niclosamide and its analogs (Nic-2, Nic-8, Nic-40, and Nic-43) for 24 hours. Briefly, Cells were washed with ice-cold PBS and lysed in harvest buffer [10 mmol/L HEPES (pH 7.9), 50 mmol/L NaCl, 500 mmol/L sucrose, 0.1 mmol/L EDTA, and 0.5% Triton X-100] supplemented with 1% protease and phosphatase inhibitors (Thermo Fisher Scientific, 78443) for 10 minutes on ice. Lysates were centrifuged at 3,000 rpm

for 3 minutes at 4 °C to separate the cytosolic fraction. The nuclear pellet was resuspended in nuclear lysis buffer [10 mmol/L HEPES (pH 7.9), 0.5 mol/L NaCl, 0.1 mmol/L EDTA, 0.1 mmol/L EGTA, and 0.1% NP-40] containing 1% inhibitors, followed by brief sonication and centrifugation at 15,000 rpm for 10 minutes to obtain nuclear extracts. Protein concentrations were measured using the BCA assay (Thermo Fisher Scientific, 23227). Western blotting was performed as described previously [2], and representative blots are shown.

**Cell viability assay**

Cell viability was assessed using trypan blue exclusion. NB16 cells were seeded in 6-well plates and treated with different concentrations of Niclosamide and its analogs (Nic-2, Nic-8, Nic-40, and Nic-43) for 24 hours. At the end of treatment, both floating and adherent cells were collected, washed once with PBS, and resuspended in fresh medium. An equal volume of 0.4% trypan blue solution (Gibco, 15250061) was added to the cell suspension and incubated for 3 minutes at room temperature. Viable and non-viable cells were counted using a hemocytometer under a light microscope. Cell viability was calculated as the percentage of unstained (viable) cells over the total cell number.

**LC–MS analysis of $NAD^+$/NADH**

The intracellular $NAD^+$/NADH ratio was determined by LC–MS. NB16 cells were treated with different concentrations of Niclosamide for 5 hours, and metabolites were extracted with 80% methanol. Metabolomic analysis was performed on an Agilent 1290 Infinity Liquid Chromatography (LC) System coupled to a Q-TOF 6545 mass spectrometer (Agilent Technologies). Targeted analysis and data processing were carried out using Agilent Profinder B.10.00 software as previously described[3]. Data are presented as mean ± SD from three biological replicates (n = 3).

**Molecular Features Analysis**

The following computational tools were used to develop the molecular features used in QSAR model development—Gaussian 16[4], Alogps[5], VegaZZ[6], and Mopac 2016[7]—to derive molecular descriptors for both the protonated (AH) and deprotonated ($A^-$) states of each compound. The workflow began with ligand preparation, where we used LigPrep[8] and Epik[9] from the Schrödinger Molecular Modelling Suite to generate protonation states within a pH range of 6.7 to 8, as well as

relevant tautomers and stereoisomers, optimized under the OPLS3e force field[10]. Conformational searches were conducted using Macromodel[11-13], applying an OPLS3e force field in a water solvent model with mixed torsional/low-mode sampling. Only conformers with an energy difference of ≤ 5.02 kcal/mol from the lowest-energy conformer were retained. The top 10 conformers for each molecule underwent to structure optimization and frequency calculations using Density Functional Theory (DFT) with the B3LYP/6-31G(d,p) level of theory and an implicit SMD water model. Further refinement included high-level optimization at B3LYP/6-311++G(d,p), followed by single-point energy calculation with MP2/aug-cc-PVDZ, providing a highly accurate electronic energy estimate[14, 15]. The Gibbs free energy was then averaged across conformers using the Boltzmann distribution, providing a weighted calculation of key molecular properties. Additional descriptors were calculated using VegaZZ in combination with the PM7 Hamiltonian (Mopac 2016), providing descriptors relevant to medicinal chemistry.

Specifically, we calculated:

1) pKa: Calculated for the phenolic hydroxyl group, relevant to bioavailability.
2) De_TOTAL: Stabilization energy of the anionic form upon protonation.
3) Dipole: The dipole moment, reflecting charge distribution, is impacted by electron-withdrawing/donating groups.
4) COSMO_AREA: Solvent-excluded surface area to highlight non-polar regions.
5) EHOMO: Energy of the highest occupied molecular orbital, indicating molecular reactivity.

We also assign the Hammett Sigma Constants for substituents R1-3 through R2-5, and the sum of ring-specific hydrophobic constants (∑π_A and ∑π_B)[16-19].

**QSAR pipeline and discussion**

In this study, we used the Scikit-learn package[20] for QSAR modeling. Specifically, we implemented an exhaustive feature combination workflow to systematically evaluate all possible descriptor combinations. This comprehensive strategy allows the identification of optimal feature subsets without relying on stepwise feature selection methods, which can be prone to local optima. Molecular features were first normalized to a mean of 0 and variance of 1 to ensure equal weighting of descriptors during model development. For model training, we employed Support Vector Regression (SVR) with an RBF kernel (C=10.0), focusing on combinations containing a fixed number of features (typically 5-6) to maintain an appropriate sample-to-feature ratio mitigate the

risk of overfitting given our limited dataset size. For model validation, we implemented leave-one-out cross-validation (LOOCV) as it maximizes the size of the training set while still providing an unbiased performance estimate. Model quality was primarily assessed using the cross-validated determination coefficient ($Q^2$), with root-mean-square error (RMSE) and mean absolute error (MAE) as supporting metrics. For each feature combination, we systematically evaluated model performance through cross-validation, stored all evaluation metrics, and identified the combinations yielding the highest predictive accuracy. The exhaustive approach ensures that all possible descriptor combinations are evaluated, avoiding the limitations of greedy optimization algorithms that may miss optimal solutions. For the best-performing feature combinations, comprehensive visualization tools were employed to interpret the models, including actual versus predicted plots, feature importance analyses, and chemical space representations. This rigorous evaluation framework ensures both statistical validity and mechanistic interpretability of the resulting QSAR models. The applicability domain was evaluated using the standardization approach proposed here[21], in which the S_value were calculated using the same algorithm described in the paper and the corresponding values were plotted against the compound IDs.

We computed a variety of electronic and steric descriptors for the Nic analogs and developed QSAR models using Support Vector Regression (SVR) to correlate these computational chemistry features with MitoTox assay results. Fourteen descriptors were initially considered, including phenolic hydroxyl pKa (pKa), stabilization energy of the anionic form upon deprotonation (De_TOTAL), dipole moment (Dipole), COSMO solvent-excluded surface area (COSMO_AREA), HOMO energy (EHOMO), ring-specific hydrophobicity constants (∑π_A and ∑π_B), and Hammett Sigma Constants (σ□ for *meta* position at R1-4, R2-3, and R2-5; and σ□ for *para* position at R1-5, and R2-4, relative to the anilide nitrogen and phenolic hydroxy)[16, 19]. Due to the influence of steric effect at the *ortho* position, the substituent constant $\sigma_0$ cannot be defined as straightforwardly as the *meta* and *para* positions. Previous comprehensive reviews by Tribble et al.[17] and Charton[18] investigated $\sigma_0$ values for various substituents. In these works, the hydroxyl chemical shifts of 2-substituted phenols and the amine chemical shifts of 2-substituted anilines were employed as surrogate measures for the *ortho* substituent constants $\sigma_0$ for position R1-3 relative to phenolic hydroxy and R2-2 relative to anilide nitrogen. To maintain an appropriate feature-to-sample ratio for the dataset (30 compounds), model fitting was constrained to 6 features, resulting in an exhaustive evaluation of 3,003 unique feature combinations (combinatorial

selection of 6 out of 14 features). Model performance exhibited concentration-dependent limitations. At 0.5 μM for both inhibitory and uncoupler activities, and at 1 μM for inhibitory activity, response distributions were largely uniform, rendering them uninformative for QSAR modeling due to minimal activity variation (**Supplement Figure 1**). Similarly, at 4 μM, model performance remained poor across all feature combinations ($Q^2$ = 0.407 for uncoupling and 0.587 for inhibition, **Supplement Figure 1)**, with applicability domain analyses provided in **Supplement Figure 2**. These findings suggest that at higher concentrations, non-structure-specific toxicity may dominate assay outcomes, potentially masking structure-activity relationships. Consequently, we focused our modeling efforts on the 2 μM dataset, where moderate structure–activity correlations were observed. To further assess the importance of each individual feature, we performed a bootstrapping analysis across models of all six-feature combinations. For each feature, we compared the mean $Q^2$ values of models with versus without the feature. The A-ring hydrophobic constant (∑π_A) had a greater influence on uncoupling activity. In contrast, the Hammett Sigma Constants at the *para* and *ortho* positions on the A-ring (R1-5_σ□ and R1-3_$\sigma_o$-phenol) played more dominant roles in respiratory inhibition. Interestingly, hydrophobic constant on B-ring (∑π_B) showed much less impact to the model fitting on both activities. The stabilization energy of the deprotonated anionic form (De_TOTAL) showed a comparable impact on both activities. COSMO_AREA slightly favored respiratory inhibition over uncoupling activity, and the HOMO energy (EHOMO) contributed modestly to the fitting of uncoupling activity but had little effect on respiratory inhibition. We also selected the top 10% of model based on $Q^2$ rankings under LOOCV, and investigated feature occurrence frequency, which corroborated the results from the bootstrapping analysis. Applicability domain analyses (**Supplement Figure 2**) revealed that only Nic-57 and Nic-58 exceeded the S_value threshold of 3. Excluding these descriptors placed all compounds within the applicability domain, confirming that structural divergence arises specifically from local inductive effects.

Collectively, our analyses indicate that the mechanisms of action for niclosamide and its analogs are multifaceted. The distinct sets of features governing uncoupling and inhibitory activities yielded inconsistent predictive performance (**Supplement Figure 1**), likely reflecting the complex, multi-target nature of the niclosamide scaffold. Model performance at higher concentrations (4 μM) was notably inferior compared to that at lower concentrations (i.e., 2 μM), indicating that the compounds may involve more than one mechanism at elevated concentrations.

Anion stabilization energy and ring-specific hydrophobicity have been thought to be key factors in predicting MitoTox assay outcomes for Nic analogs. Though our QSAR results revealed limited differentiation in the descriptor space contributing to uncoupling versus inhibitory activity, we would like to conclude three aspects on structural features of niclosamide and its analogs: 1) 5-chloro substitution at the position parallel to the hydroxyl group in niclosamide appears to contribute more to toxicity than to uncoupling. This is evident when comparing Nic-2 and Nic-54 to the parent compound (Nic) and is supported by bootstrapped leave-one-out cross-validation, where the R1-5_σp feature showed significantly greater importance for toxicity than for uncoupling; 2) A-ring substitution hydrophobicity is more strongly associated with uncoupling than toxicity. For example, Nic-54 and Nic-55 at 2 μM differ in their uncoupling activity, which aligns with the ∑π_A feature ranking much higher for uncoupling than for toxicity; 3) Electronic and steric descriptors: HOMO energy (EHOMO) is more influential for uncoupling activity, while COSMO_AREA has a greater impact on respiratory inhibition. B-ring hydrophobicity (∑π_B) contributes minimally to either effect, while anion stabilization energy (De_TOTAL) influences both. Therefore, these findings suggest: (i) To enhance uncoupling, retain a weakly acidic, membrane-permeable protonophore (i.e., phenolic OH with adequate A-ring hydrophobicity and mild electron-withdrawing groups on both rings); (ii) To reduce toxicity, avoid strong electron-withdrawing groups on the A-ring and large non-polar surface areas.

**Chemistry**

**Organic Synthesis**

Commercial reagents were used as received, without further purification. Anhydrous grade solvents were purchased and utilized directly without additional treatment. Flash column chromatography was performed using a CombiFlash instrument fitted with Flash Pure Buchi columns. Proton nuclear magnetic resonance ($^1$H NMR) spectra were obtained on a 400 MHz Bruker spectrometer. High-resolution electrospray ionization mass spectrometry (HR-ESI-MS) spectra were recorded on a Thermo Fisher Orbitrap Velos with an autosampler. Low-resolution mass spectrometry was conducted via liquid chromatography–mass spectrometry (LC-MS) on a Waters Acquity UPLC system, using atmospheric pressure chemical ionization (APCI) or electrospray ionization (ESI) as required.

The general synthetic scheme of all the Niclosamide analogs is shown below (**scheme 1**).

**Scheme 1**

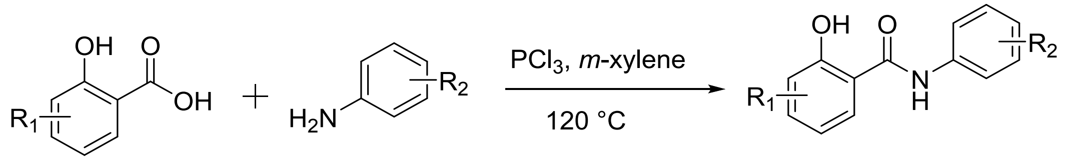


To the solution of corresponding carboxylic acids (1.0 eq) in *m*-xylene was added corresponding anilines (1.1 eq) and heated to 110°C. After 20 min, $PCl_3$ (0.4 mmol) was added to reaction mixture and raised the temperature to 120 °C and stirred for another 3 hours. The reaction mixture was brought to 80°C and diluted with water. Major of the compounds are precipitated and filtered off, washed with Hexane and pet-ether and dried. The compounds which are not precipitated, followed the Combi flash purification by using EtOAc in Hexane system.

**5-chloro-2-hydroxy-N-phenylbenzamide** (**Nic-0**) [22]

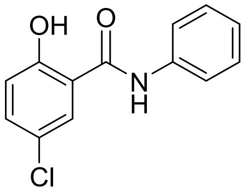

HNMR (300 MHz, DMSO-$d_6$) δ 11.85 (s, 1H), 10.41 (s, 1H), 7.96 (d, *J* = 2.6 Hz, 1H), 7.70 (d, *J* = 7.6 Hz, 2H), 7.47 (dd, *J* = 8.8 Hz, 2.7 Hz, 1H), 7.38 (t, *J* = 7.5 Hz, 2H), 7.15 (t, *J* = 7.3 Hz, 1H), 7.01 (d, *J* = 8.7 Hz, 1H), LC-MS (ESI); $[M-H]^-$: 246.0

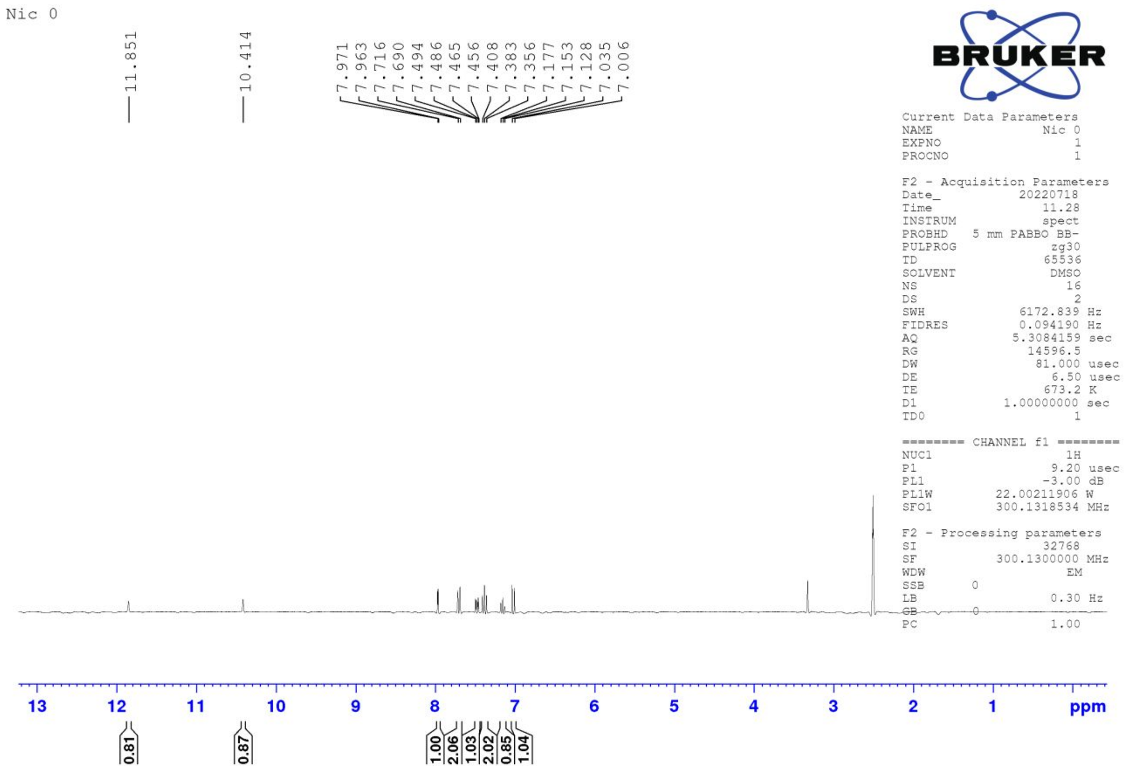

Vial:2:6
Date:16-Sep-2024
Method:C:\MassLynx\OpenLynx_Methods\ESI-_100-1250+PDA.olp
MS Method:OA_ESI-_Default
ID:mohammed210_147-11-20240916-1113
Time:13:11:41
Inlet Method:OA_Default
File:mohammed210_147-11-20240916-1113_COD
Description:NIC-0
Instrument:ACQ-QDA#KBD6021
Detectors:Waters Acquity PDA

Printed: Mon Sep 16 13:31:21 2024

**Sample Report (continued):**

**Sample 11 NIC-0 16-Sep-2024 13:11:41 File: mohammed210_147-11-20240916-1113_COD**

1: MS ES- :TIC Smooth (SG, 2x3) 2.5e+006

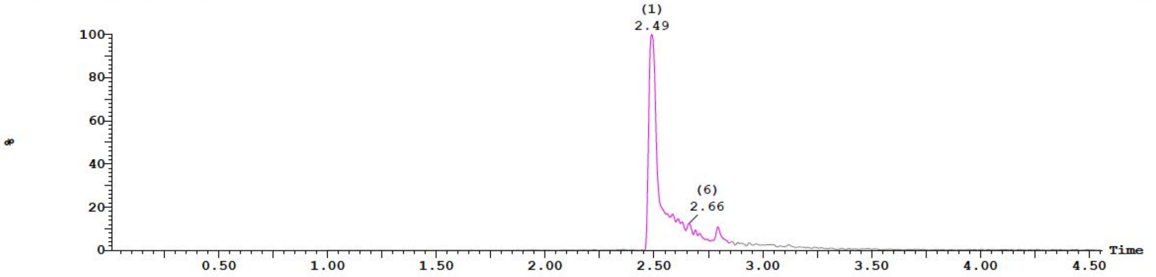


2: UV Detector: TAC: Wavelength Range: (210 - 499) 3.087e+1
Range: 3.087e+1

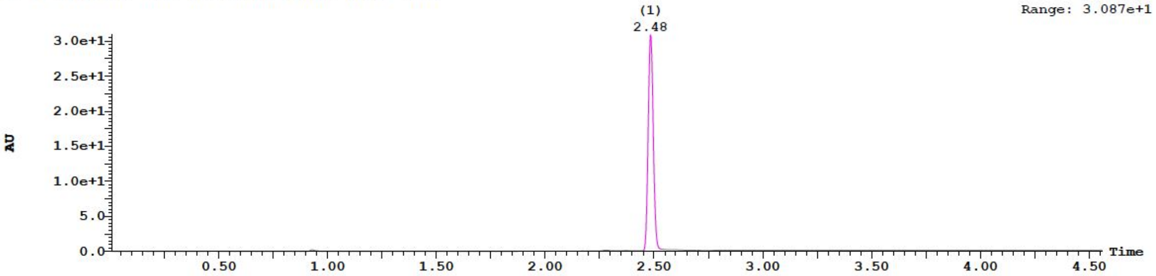


**Sample Report (continued):**

| Peak ID | Time |
|---|---|
| 1 | 2.49 |

1:(Time: 2.48) Combine (415:475-(385:414+476:505)) 1:MS ES- 3.0e+005

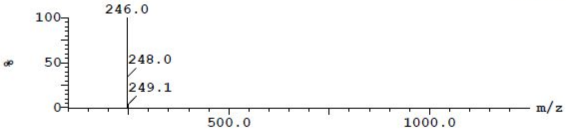


| Peak ID | Time |
|---|---|
| 1 | 2.49 |

1:(Time: 2.48) Combine (743) 2:UV Detector 4.291e-1 AU

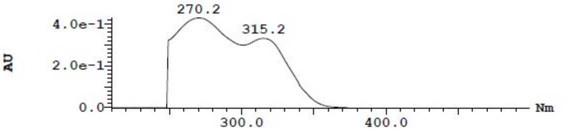


| Peak ID | Time |
|---|---|
| 2 | 2.56 |

2:(Time: 2.56) Combine (438:498-(408:437+499:528)) 1:MS ES- 4.3e+005

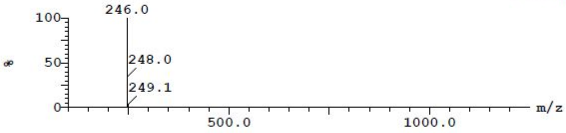


| Peak ID | Time |
|---|---|
| 3 | 2.59 |

3:(Time: 2.59) Combine (446:506-(416:445+507:536)) 1:MS ES- 2.9e+005

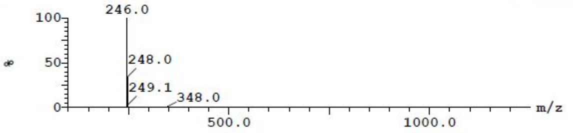


| Peak ID | Time |
|---|---|
| 4 | 2.61 |

4:(Time: 2.61) Combine (454:514-(424:453+515:544)) 1:MS ES- 2.2e+003

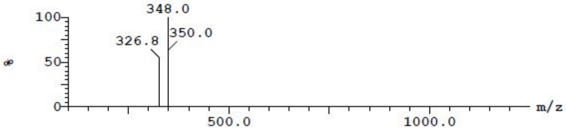


| Peak ID | Time |
|---|---|
| 5 | 2.63 |

5:(Time: 2.63) Combine (459:519-(429:458+520:549)) 1:MS ES- 3.3e+003

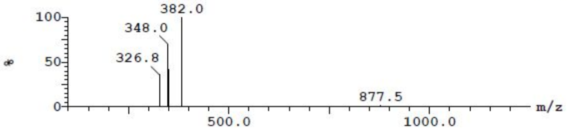

**3-chloro-N-(2-chloro-4-nitrophenyl)benzamide (Nic-1)**[22]

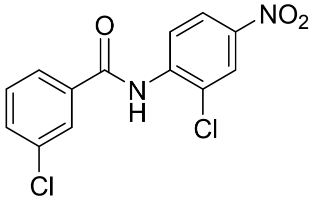


$^{1}$HNMR (400 MHz, DMSO-$d_6$) δ 10.46 (s, 1H), 8.41 (d, *J* = 2.5 Hz, 1H), 8.26 (dd, *J* = 8.9 Hz, 2.6 Hz, 1H), 8.04 - 8.0 (m, 2H), 7.96 - 7.94 (m, 1H), 7.74 - 7.71 (m, 1H), 7.61 (t, *J* = 7.8 Hz, 1H); LC-MS (ESI); $[M-H]^-$: 308.9

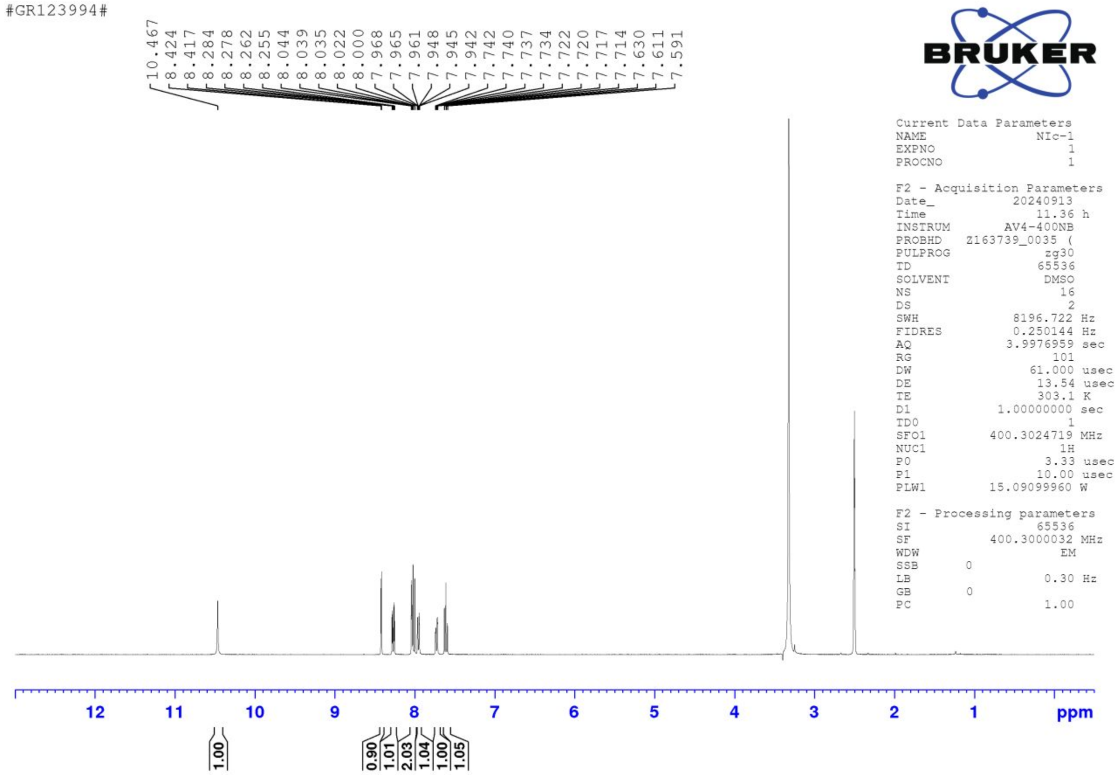

Vial:1:45 | ID:mohammed210_147-02-20240916-1113 | File:mohammed210_147-02-20240916-1113_COD
Date:16-Sep-2024 | Time:12:18:03 | Description:NIC-1
Method:C:\MassLynx\OpenLynx_Methods\ESI-_100-1250+PDA.olp | Instrument:ACQ-QDA#KBD6021
MS Method:OA_ESI-_Default | Inlet Method:OA_Default | Detectors:Waters Acquity PDA

Printed: Mon Sep 16 13:31:21 2024

**Sample Report (continued):**

**Sample 2 NIC-1 16-Sep-2024 12:18:03 File: mohammed210_147-02-20240916-1113_COD**

1: MS ES- :TIC Smooth (SG, 2x3) 1.3e+006

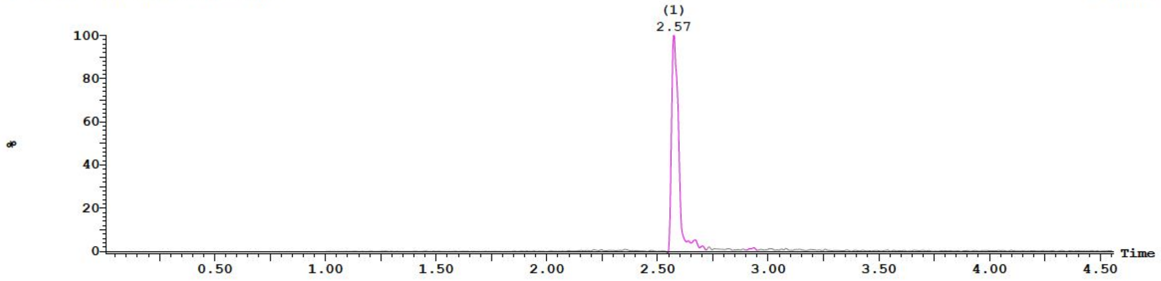

2: UV Detector: TAC: Wavelength Range: (210 - 499) 2.42e+1 Range: 2.42e+1

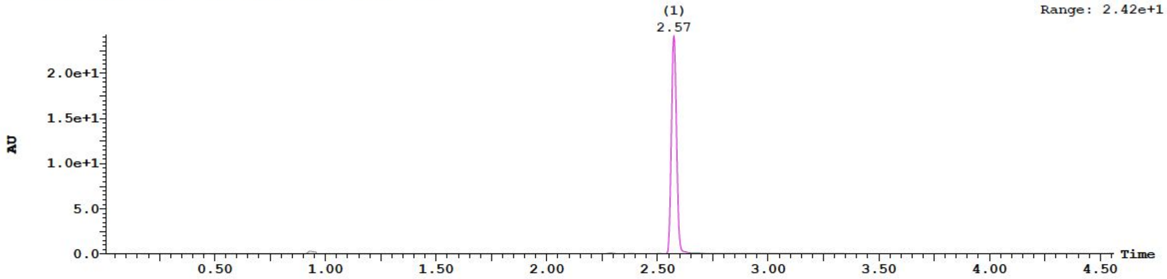

**Sample Report (continued):**

**Peak ID** 1 **Time** 2.57
1:(Time: 2.57) Combine (442:502-(412:441+503:532)) 1:MS ES- 1.2e+005

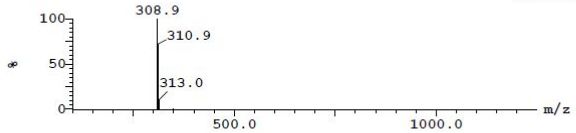

**Peak ID** 1 **Time** 2.57
1:(Time: 2.57) Combine (770) 2:UV Detector 3.341e-1 AU

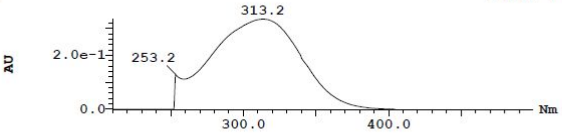

**Peak ID** 2 **Time** 2.64
2:(Time: 2.64) Combine (463:523-(433:462+524:553)) 1:MS ES- 1.3e+005

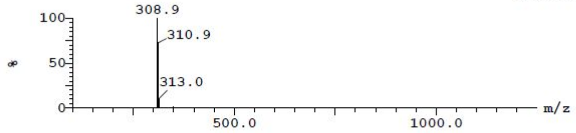

**Peak ID** 3 **Time** 2.67
3:(Time: 2.67) Combine (471:531-(441:470+532:561)) 1:MS ES- 9.0e+004

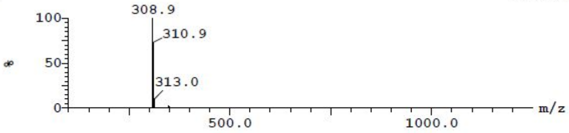

**Peak ID** 4 **Time** 2.70
4:(Time: 2.70) Combine (481:541-(451:480+542:571)) 1:MS ES- 2.0e+003

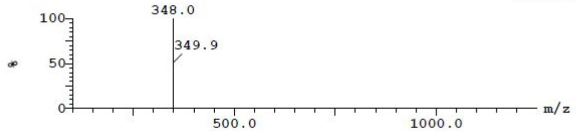

**Peak ID** 5 **Time** 2.94
5:(Time: 2.94) Combine (551:611-(521:550+612:641)) 1:MS ES- 4.9e+002

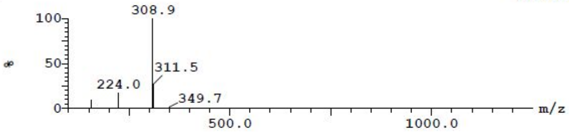

**N-(2-chloro-4-nitrophenyl)-2-hydroxybenzamide (Nic-2)**[22]

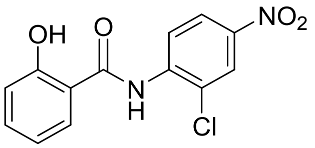


$^1$HNMR (400 MHz, DMSO-d$_6$) δ 12.12 (s, 1H), 11.37 (s, 1H), 8.83 (d, *J* = 9.2 Hz, 1H), 8.40 (d, *J* = 2.5 Hz, 1H), 8.26 (dd, *J* = 9.2 Hz, 2.5 Hz, 1H), 8.03 (dd, *J* = 7.8 Hz, 1.4 Hz, 1H), 7.48 (td, *J* = 8.4 Hz, 1.5 Hz, 1H), 7.07 - 7.00 (m, 2H); LC-MS (ESI); [M-H]$^-$: 290.9

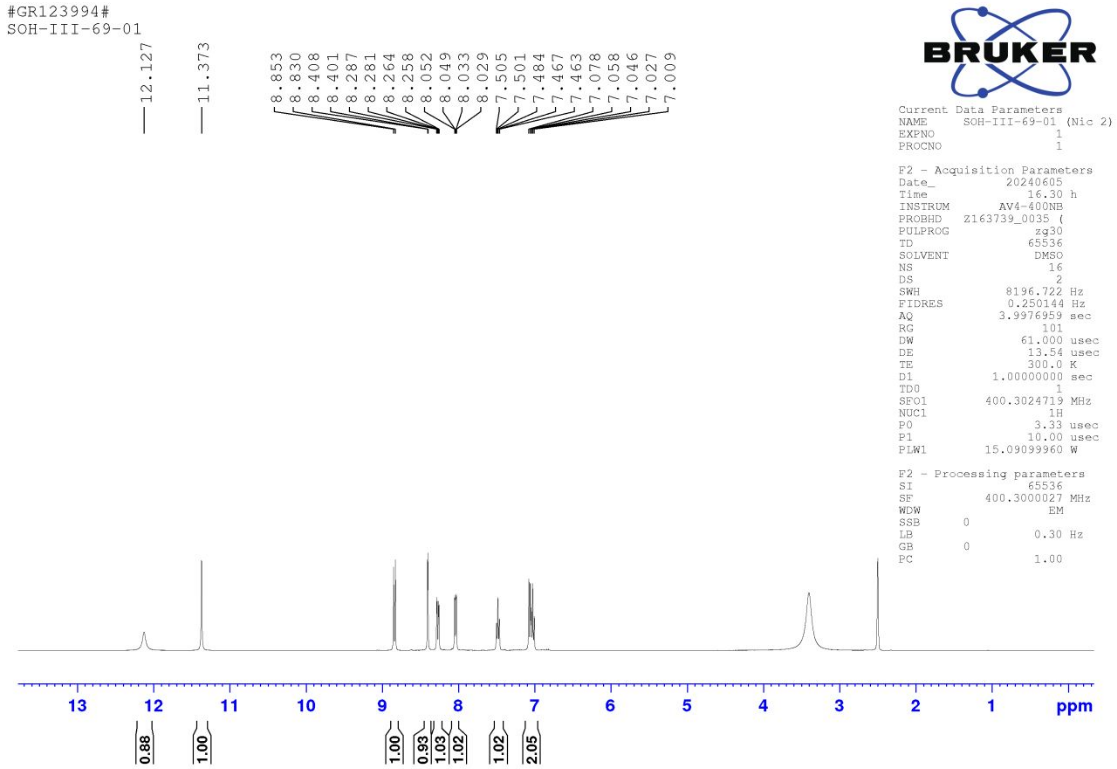

Vial:1:12
Date:19-Sep-2024
Method:C:\MassLynx\OpenLynx_Methods\ESI-_100-1250+PDA.olp
MS Method:OA_ESI-_Default
ID:mohammed210_149-2-20240919-1212
Time:12:25:13
Inlet Method:OA_Default
File:mohammed210_149-2-20240919-1212_COD
Description:Nic-2
Instrument:ACQ-QDA#KBD6021
Detectors:Waters Acquity PDA

Printed: Thu Sep 19 13:03:11 2024

**Sample Report (continued):**

**Sample 2 Nic-2 19-Sep-2024 12:25:13 File: mohammed210_149-2-20240919-1212_COD**

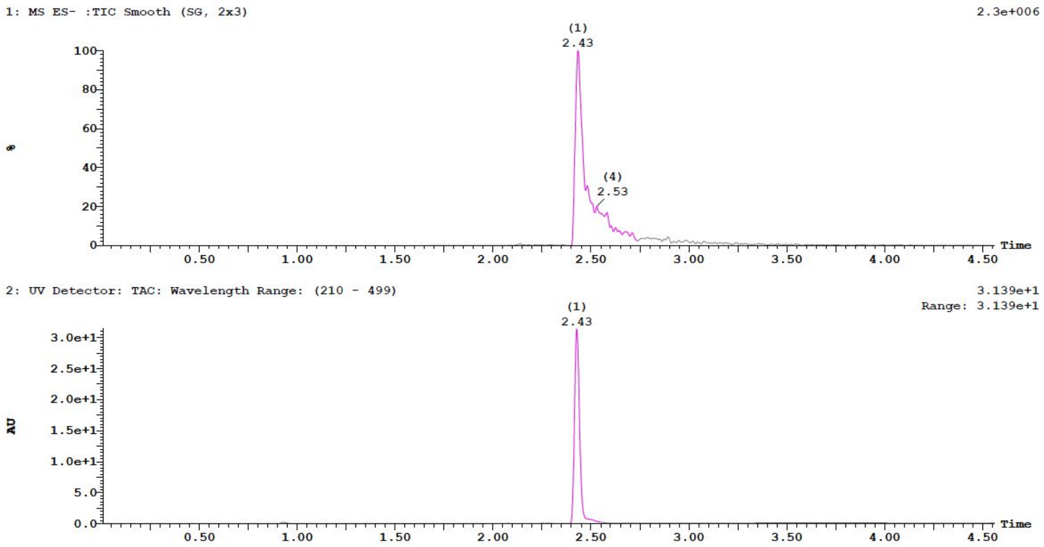


**Sample Report (continued):**

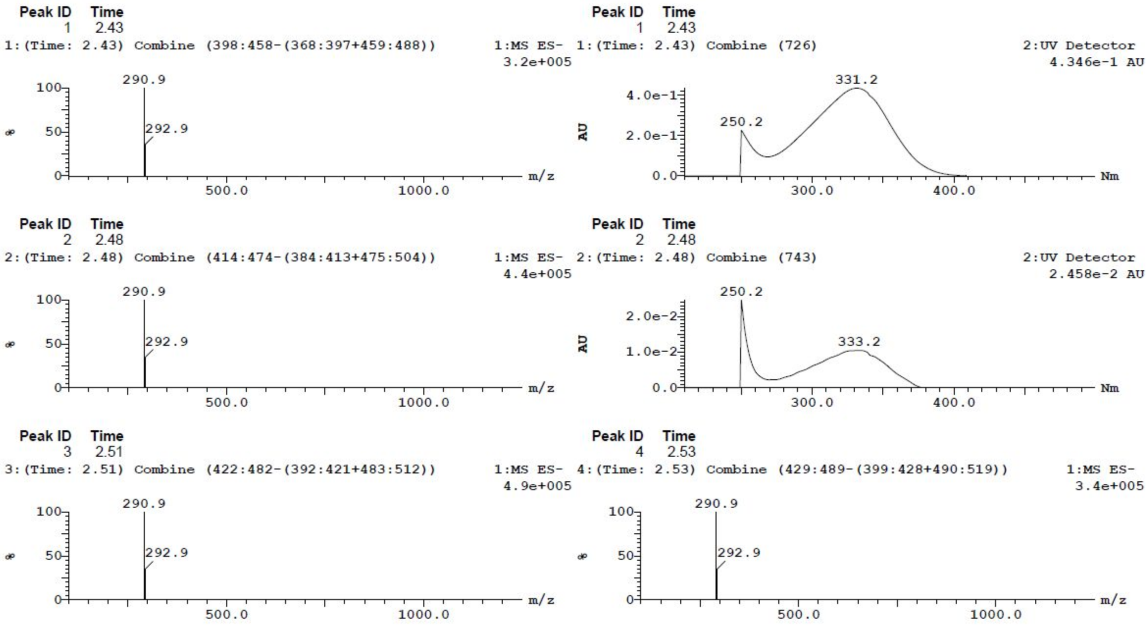

**5-chloro-2-hydroxy-N-(4-nitrophenyl)benzamide** (**Nic 3**)[23]

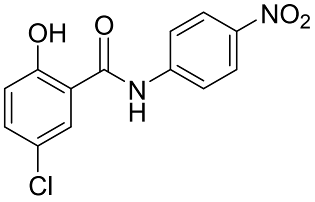


$^1$HNMR (400 MHz, $CD_3OD$) δ 8.28 (d, *J* = 9.2 Hz, 2H), 8.02 - 7.97 (m, 3H), 7.44 (dd, *J* = 8.8 Hz, 2.6 Hz, 1H), 6.99 (d, *J* = 8.8 Hz, 1H); LC-MS (ESI); $[M+H]^+$: 315.6

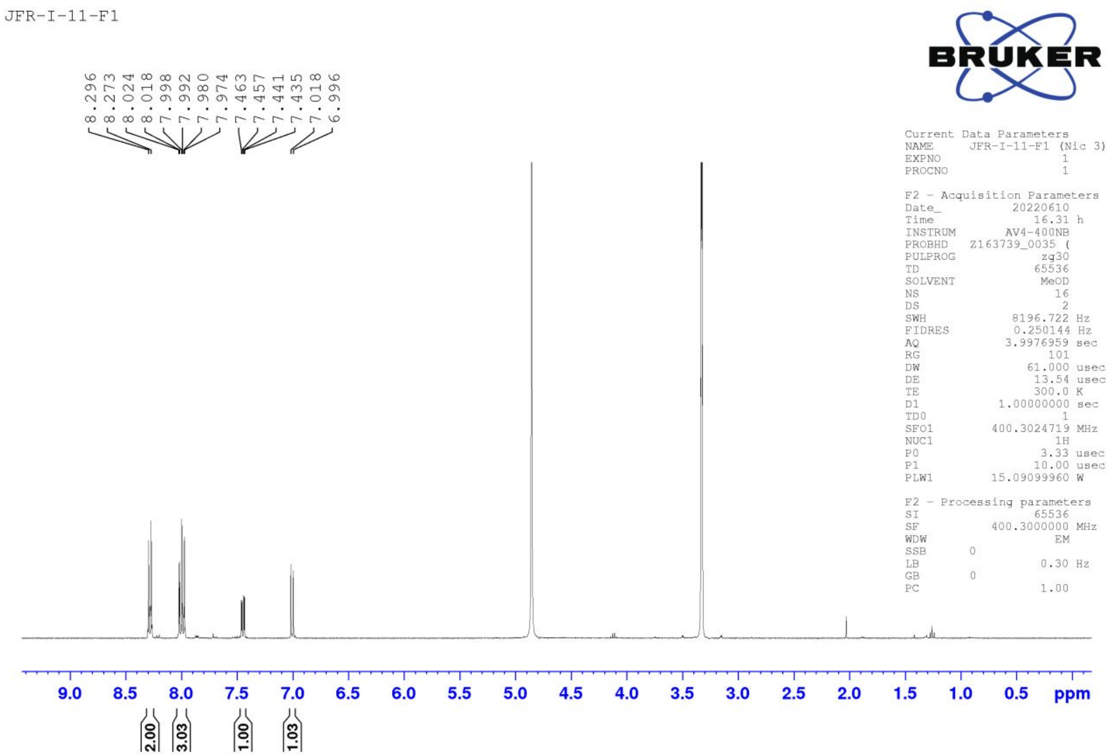

Vial:1:13
Date:19-Sep-2024
Method:C:\MassLynx\OpenLynx_Methods\ESI-_100-1250+PDA.olp
MS Method:OA_ESI-_Default

ID:mohammed210_149-3-20240919-1212
Time:12:31:11
Inlet Method:OA_Default

File:mohammed210_149-3-20240919-1212_COD
Description:Nic-3
Instrument:ACQ-QDA#KBD6021
Detectors:Waters Acquity PDA

Printed: Thu Sep 19 13:03:11 2024

**Sample Report (continued):**

**Sample 3 Nic-3 19-Sep-2024 12:31:11 File: mohammed210_149-3-20240919-1212_COD**

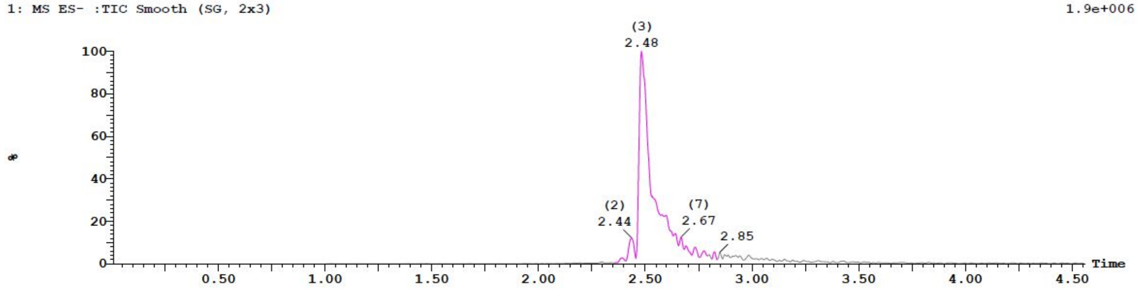


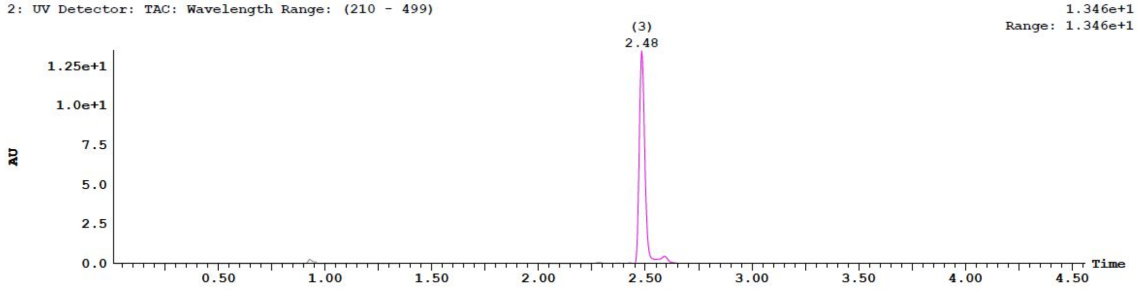


**Sample Report (continued):**

**Peak ID** 1 **Time** 2.39

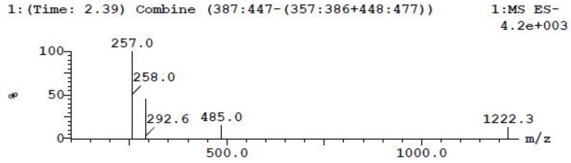


**Peak ID** 2 **Time** 2.44

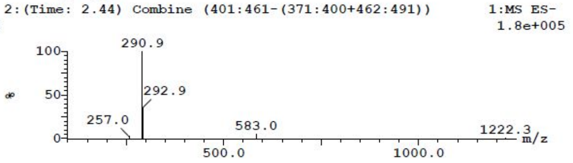


**Peak ID** 3 **Time** 2.48

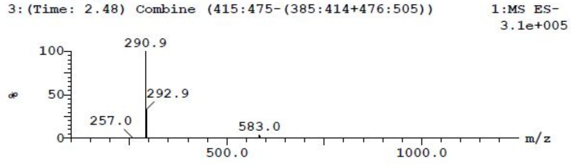


**Peak ID** 3 **Time** 2.48

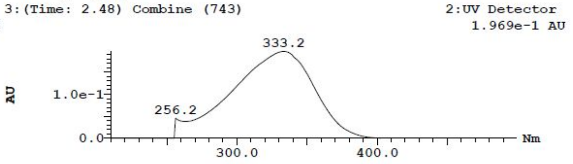


**Peak ID** 4 **Time** 2.53

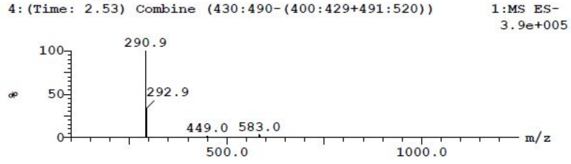


**Peak ID** 5 **Time** 2.60

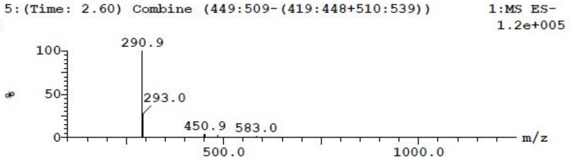

**5-chloro-N-(2-chloro-4-fluorophenyl)-2-hydroxybenzamide** (Nic-4)[24]

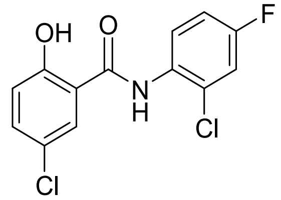

$^{1}$HNMR (400 MHz, DMSO-$d_6$) δ 12.22 (s, 1H), 10.78 (s, 1H), 8.32 - 8.28 (m, 1H), 7.98 (d, *J* = 2.8 Hz, 1H), 7.59 -7.49 (m, 2H), 7.32 -7.27(m, 1H), 7.06 (d, *J* = 8.7 Hz, 1H); LC-MS (ESI); [M-H]$^-$: 297.9

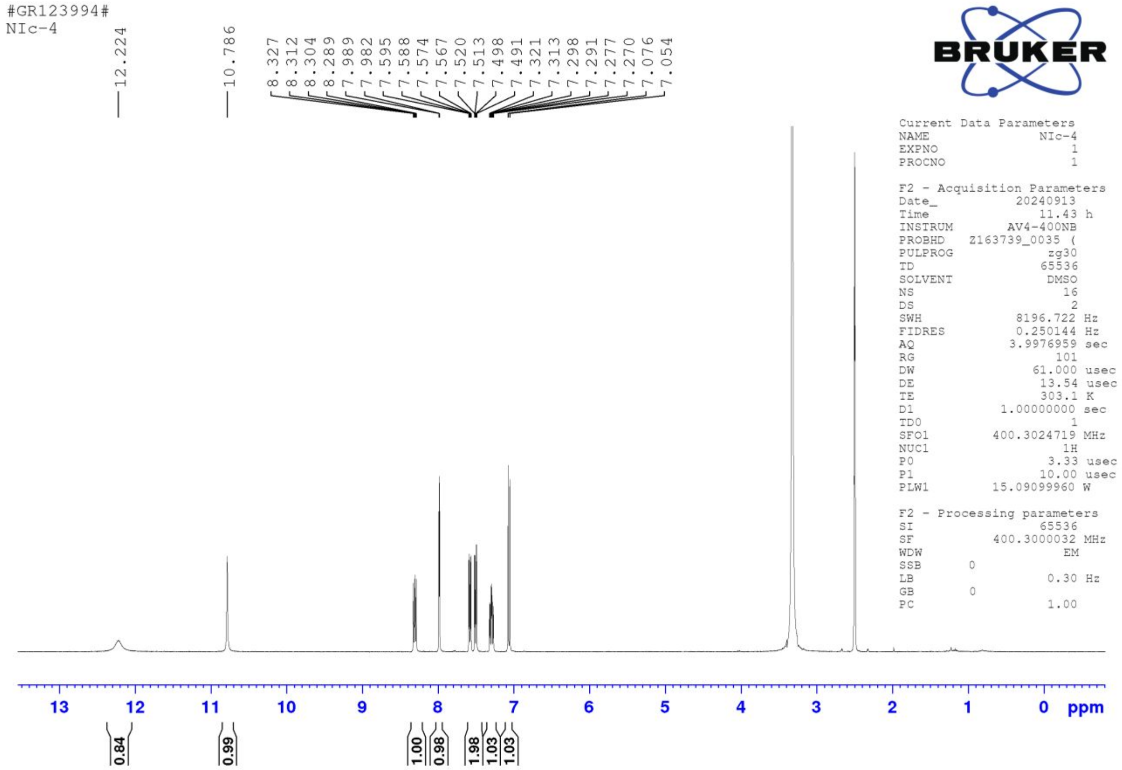

Vial:1:48
Date:16-Sep-2024
Method:C:\MassLynx\OpenLynx_Methods\ESI-_100-1250+PDA.olp
MS Method:OA_ESI-_Default

ID:mohammed210_147-05-20240916-1113
Time:12:35:59
Inlet Method:OA_Default

File:mohammed210_147-05-20240916-1113_COD
Description:NIC-4
Instrument:ACQ-QDA#KBD6021
Detectors:Waters Acquity PDA

Printed: Mon Sep 16 13:31:21 2024

**Sample Report (continued):**

**Sample 5 NIC-4 16-Sep-2024 12:35:59 File: mohammed210_147-05-20240916-1113_COD**

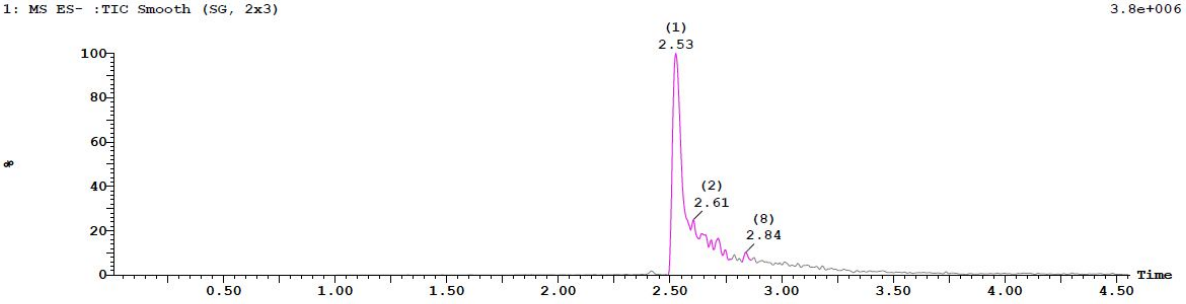


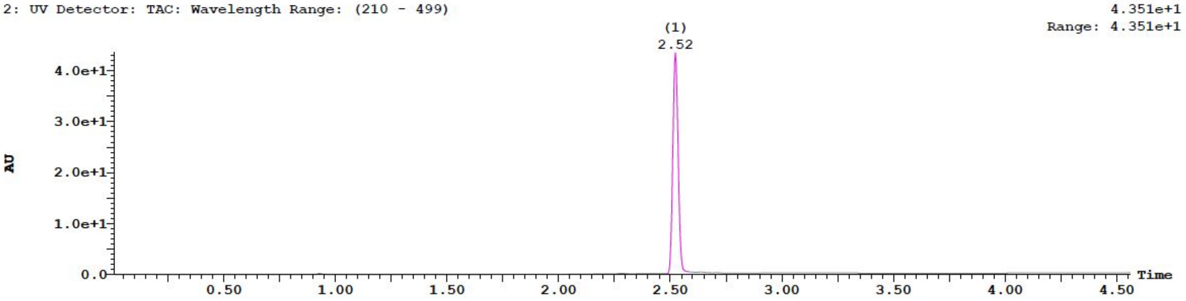


**Sample Report (continued):**

Peak ID 1 Time 2.53

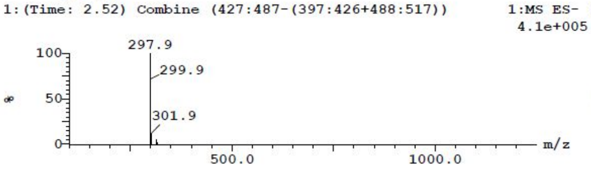


Peak ID 1 Time 2.53

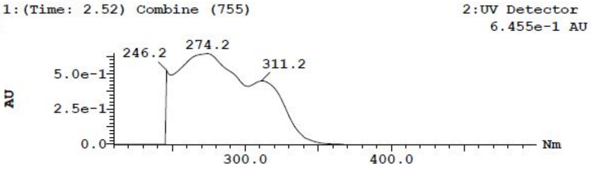


Peak ID 2 Time 2.61

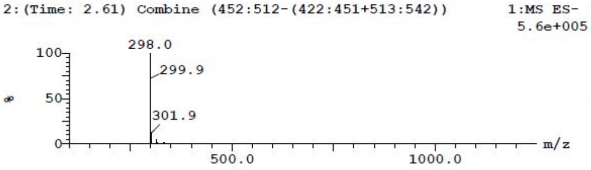


Peak ID 3 Time 2.64

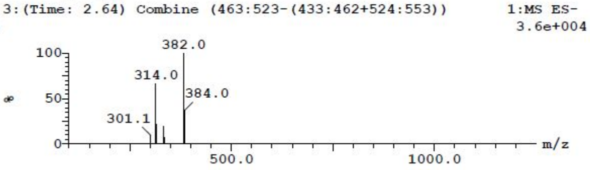


Peak ID 4 Time 2.66

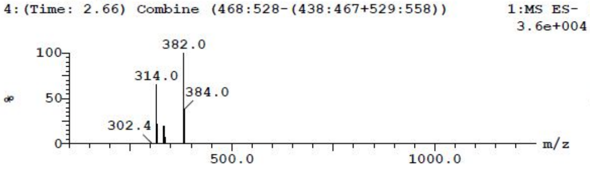


Peak ID 5 Time 2.69

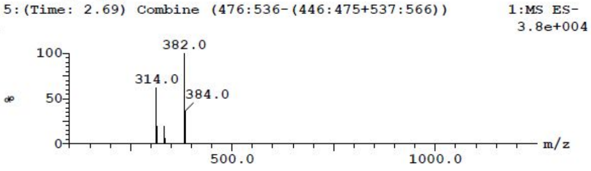

**N-(3,5-bis(trifluoromethyl)phenyl)-5-chloro-2-hydroxybenzamide (Nic 5)[25]:**

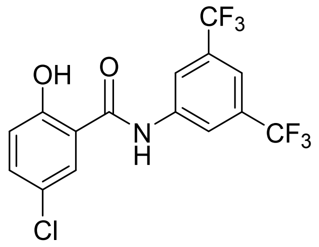

$^1$HNMR (400 MHz, $CDCl_3$) δ 11.35 (s, 1H), 8.16 - 8.11 (m, 3H), 7.74 (s, 1H), 7.55 (d, *J* = 2.4 Hz, 1H), 7.47 (dd, *J* = 8.8 Hz, 2.4 Hz, 1H), 7.05 (d, *J* = 8.8 Hz, 1H); LC-MS (ESI); $[M-H]^-$: 382.0

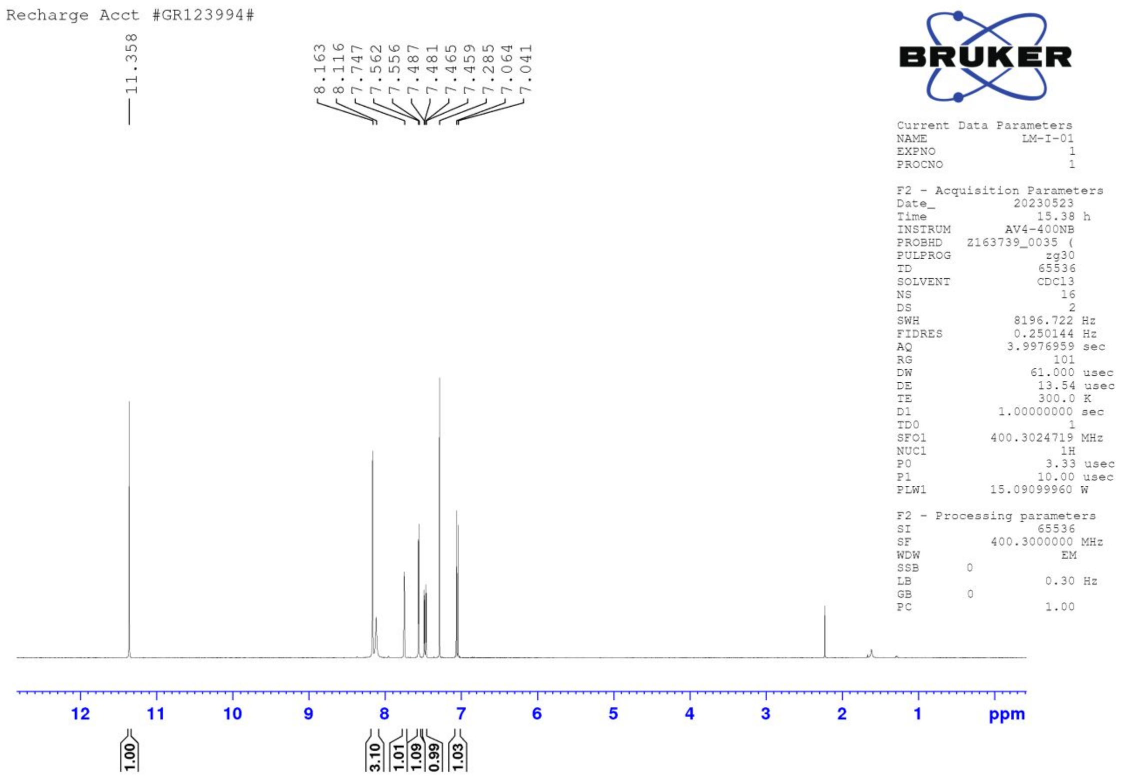

**Openlynx Report – mohammed210**

Vial:1:46
Date:16-Sep-2024
Method:C:\MassLynx\OpenLynx_Methods\ESI-_100-1250+PDA.olp
MS Method:OA_ESI-_Default
ID:mohammed210_147-03-20240916-1113
Time:12:24:03
Inlet Method:OA_Default
File:mohammed210_147-03-20240916-1113_COD
Description:LM-I-01
Instrument:ACQ-QDA#KBD6021
Detectors:Waters Acquity PDA

Printed: Mon Sep 16 13:31:21 2024

**Sample Report (continued):**

**Sample 3 LM-I-01 16-Sep-2024 12:24:03 File: mohammed210_147-03-20240916-1113_COD**

1: MS ES- :TIC Smooth (SG, 2x3) 3.4e+006

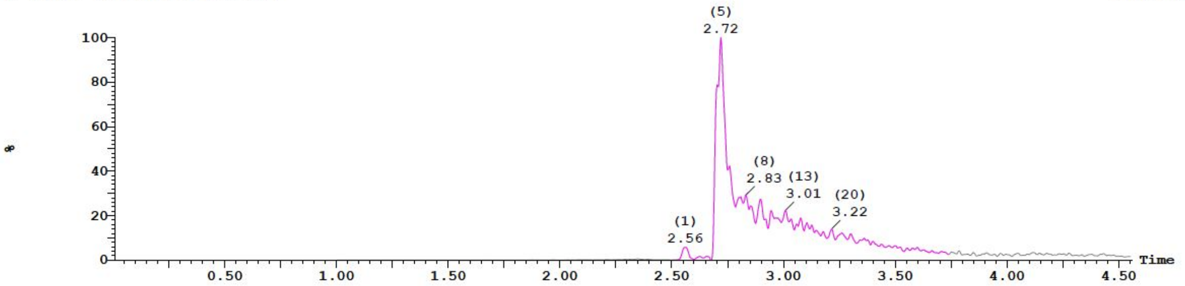


2: UV Detector: TAC: Wavelength Range: (210 - 499) 1.188e+2 Range: 1.188e+2

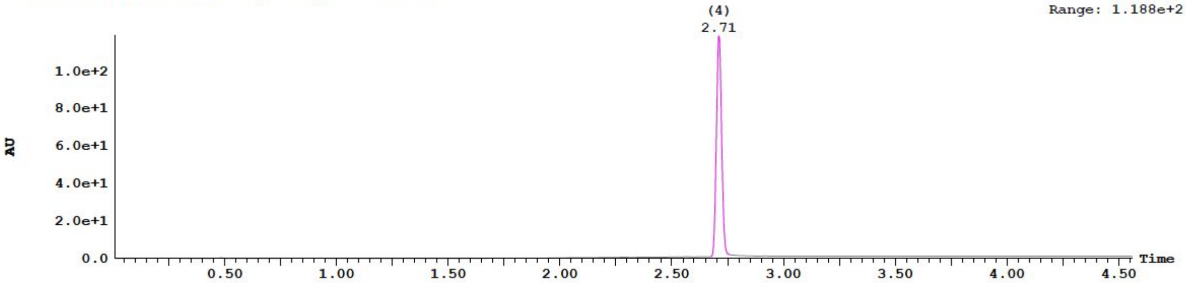


**Sample Report (continued):**

**Peak ID** 1 **Time** 2.56
1: (Time: 2.56) Combine (438:498-(408:437+499:528)) 1:MS ES- 1.5e+004

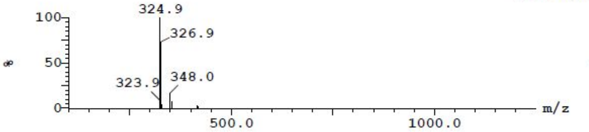


**Peak ID** 2 **Time** 2.63
2: (Time: 2.63) Combine (458:518-(428:457+519:548)) 1:MS ES- 1.5e+004

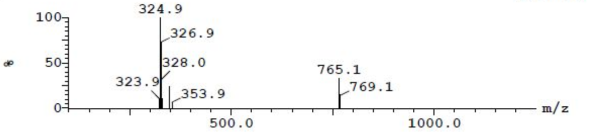


**Peak ID** 3 **Time** 2.66
3: (Time: 2.66) Combine (467:527-(437:466+528:557)) 1:MS ES- 1.7e+005

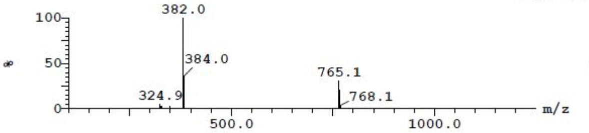


**Peak ID** 4 **Time** 2.70
4: (Time: 2.71) Combine (483:543-(453:482+544:573)) 1:MS ES- 3.6e+005

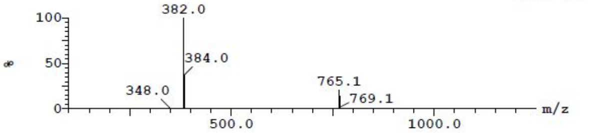


**Peak ID** 4 **Time** 2.70
4: (Time: 2.71) Combine (811) 2:UV Detector 1.907 AU

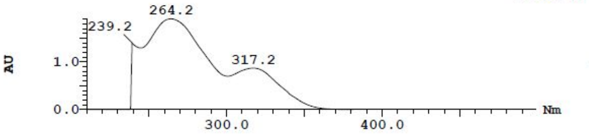


**Peak ID** 5 **Time** 2.72
5: (Time: 2.72) Combine (486:546-(456:485+547:576)) 1:MS ES- 3.9e+005

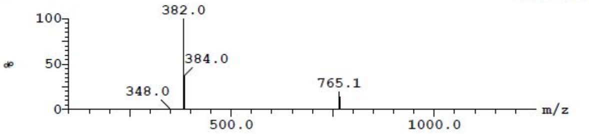

**5-chloro-2-hydroxy-N-(3-(trifluoromethyl)phenyl)benzamide (Nic-6)**[22]

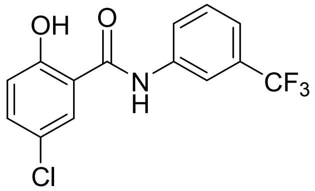

$^1$HNMR (400 MHz, DMSO-$d_6$) δ 11.62 (s, 1H), 7.91 (bs, 2H), 7.56 - 7.41 (m, 4H), 7.01 (dd, *J* = 8.8 Hz, 1H); LC-MS (ESI); [M-H]$^-$: 314.0

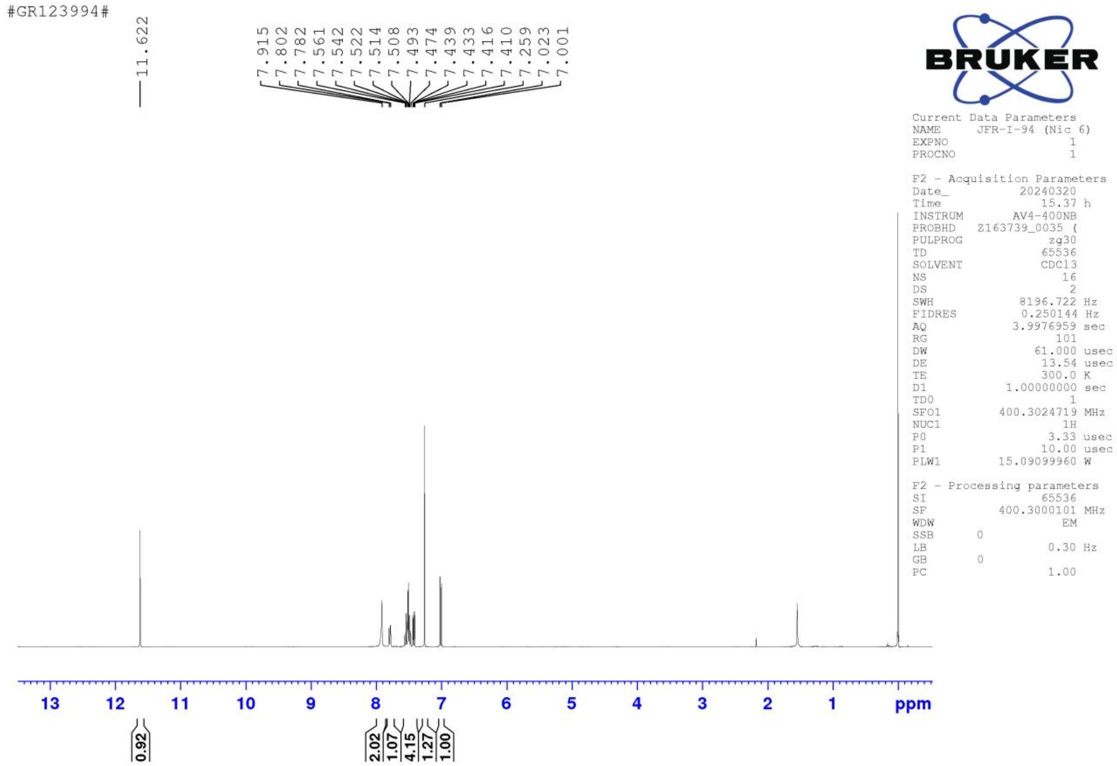

**Openlynx Report – mohammed210**

| | | |
|---|---|---|
| Vial:1:47 | ID:mohammed210_147-04-20240916-1113 | File:mohammed210_147-04-20240916-1113_COD |
| Date:16-Sep-2024 | Time:12:30:00 | Description:NIC-6 |
| Method:C:\MassLynx\OpenLynx_Methods\ESI-_100-1250+PDA.olp | | Instrument:ACQ-QDA#KBD6021 |
| MS Method:OA_ESI-_Default | Inlet Method:OA_Default | Detectors:Waters Acquity PDA |

Printed: Mon Sep 16 13:31:21 2024

**Sample Report (continued):**

**Sample 4 NIC-6 16-Sep-2024 12:30:00 File: mohammed210_147-04-20240916-1113_COD**

1: MS ES- :TIC Smooth (SG, 2x3) 4.1e+006

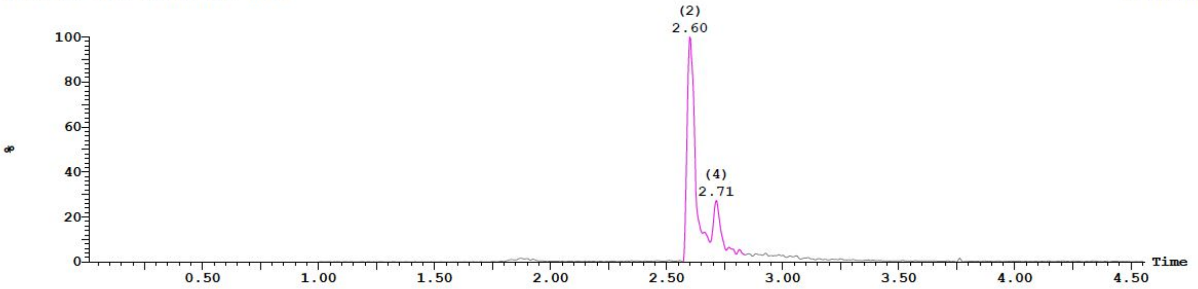


2: UV Detector: TAC: Wavelength Range: (210 - 499) 9.911
Range: 9.911

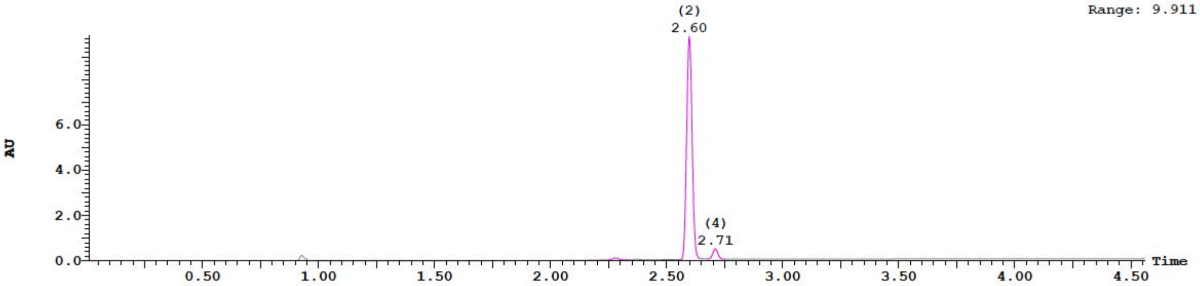


**Sample Report (continued):**

**Peak ID** **Time**
1 2.28

1:(Time: 2.28) Combine (354:414-(324:353+415:444)) 1:MS ES- 7.9e+002

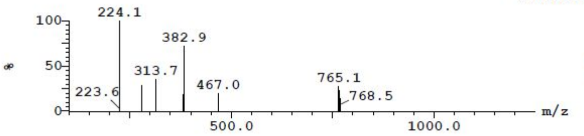


**Peak ID** **Time**
1 2.28

1:(Time: 2.28) Combine (682) 2:UV Detector 1.366e-2 AU

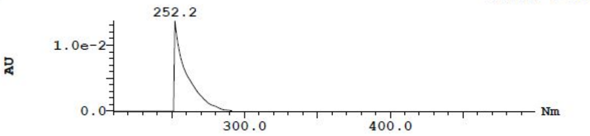


**Peak ID** **Time**
2 2.60

2:(Time: 2.60) Combine (449:509-(419:448+510:539)) 1:MS ES- 5.7e+005

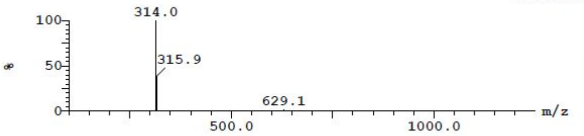


**Peak ID** **Time**
2 2.60

2:(Time: 2.60) Combine (777) 2:UV Detector 1.684e-1 AU

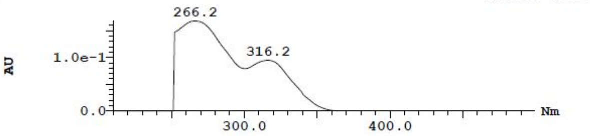


**Peak ID** **Time**
3 2.66

3:(Time: 2.66) Combine (469:529-(439:468+530:559)) 1:MS ES- 5.7e+005

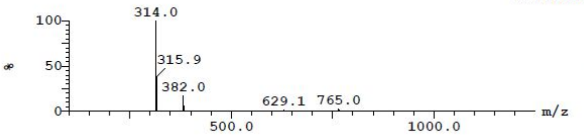


**Peak ID** **Time**
4 2.71

4:(Time: 2.71) Combine (484:544-(454:483+545:574)) 1:MS ES- 1.1e+005

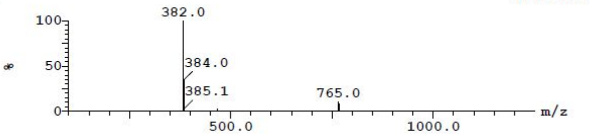

**5-chloro-N-(2-chloro-4-(trifluoromethyl)phenyl)-2-hydroxybenzamide** (Nic-7)[26]

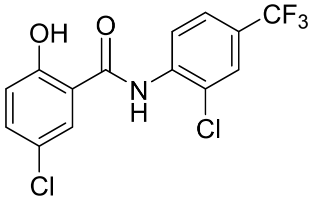


$^{1}$HNMR (400 MHz, DMSO-$d_6$) δ 12.4 (s, 1H), 11.17 (s, 1H), 8.73 (d, *J* = 8.4 Hz, 1H), 8.0 - 7.98 (m, 2H), 7.79 (dd, *J* = 8.7 Hz, 1.6 Hz, 1H), 7.53 (dd, *J* = 8.7 Hz, 2.8 Hz, 1H), 7.10 (d, *J* = 4.3 Hz, 1H); LC-MS (ESI); [M-H]$^-$: 348.0

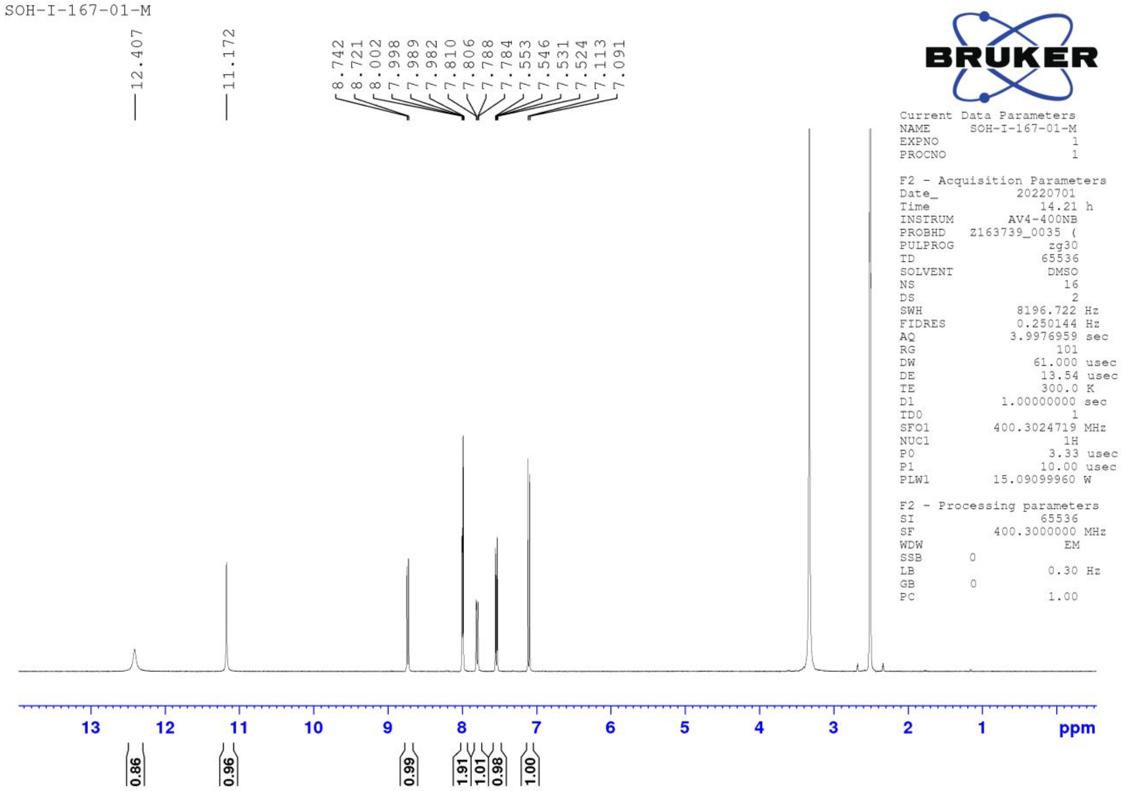

**OSU College of Pharmacy Shared Instrumentation Facility**
**Openlynx Report – mohammed210**

| | | |
|---|---|---|
| Vial:2:2 | ID:mohammed210_147-07-20240916-1113 | File:mohammed210_147-07-20240916-1113_COD |
| Date:16-Sep-2024 | Time:12:47:55 | Description:SOH-I-167-01 |
| Method:C:\MassLynx\OpenLynx_Methods\ESI-_100-1250+PDA.olp | | Instrument:ACQ-QDA#KBD6021 |
| MS Method:OA_ESI-_Default | Inlet Method:OA_Default | Detectors:Waters Acquity PDA |

Printed: Mon Sep 16 13:31:21 2024

**Sample Report (continued):**

**Sample 7 SOH-I-167-01 16-Sep-2024 12:47:55 File: mohammed210_147-07-20240916-1113_COD**

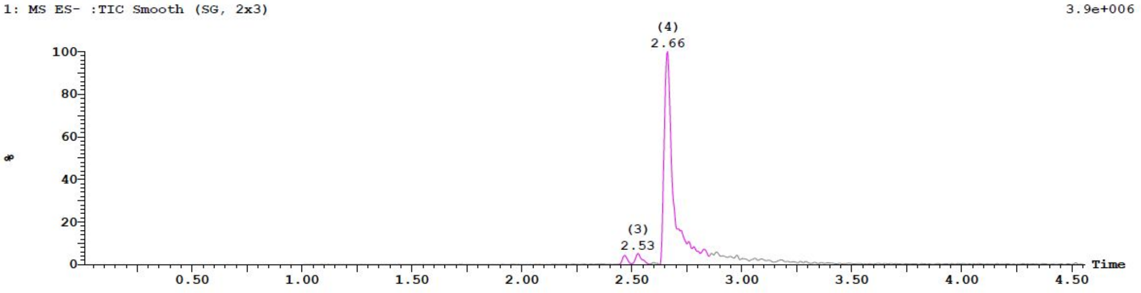


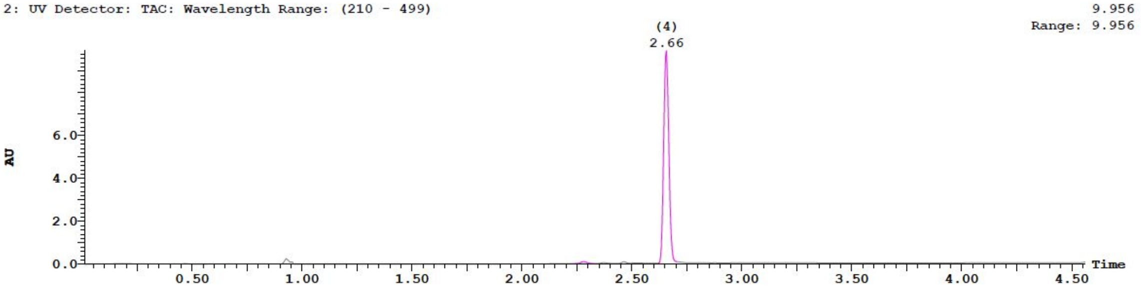


**Sample Report (continued):**

**Peak ID** 1 **Time** 2.28

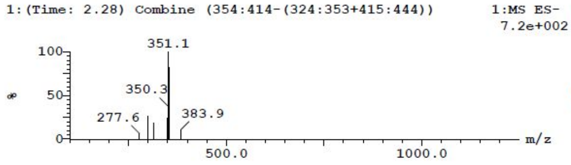


**Peak ID** 1 **Time** 2.28

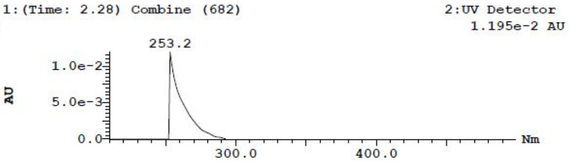


**Peak ID** 2 **Time** 2.47

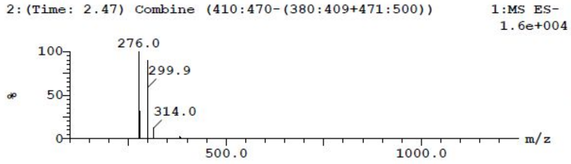


**Peak ID** 3 **Time** 2.53

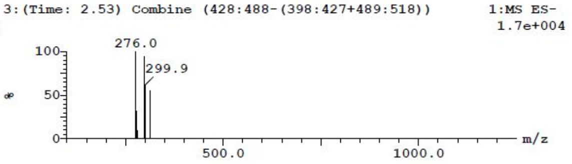


**Peak ID** 4 **Time** 2.66

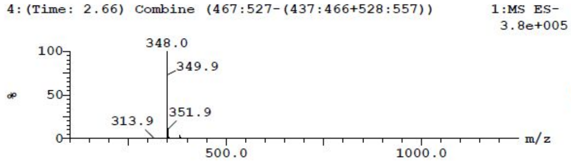


**Peak ID** 4 **Time** 2.66

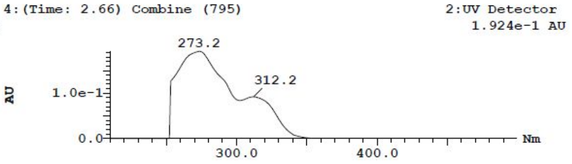

**5-chloro-N-(2-chlorophenyl)-2-hydroxybenzamide** (**Nic-8**)[23]

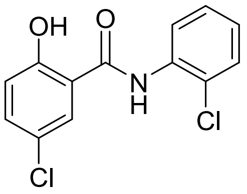


$^{1}$HNMR (400 MHz, DMSO-$d_6$) δ 12.25 (s, 1H), 10.88 (s, 1H), 8.39 (dd, *J* = 8.2 Hz, 1.48 Hz, 1H), 7.99 (d, *J* = 2.7 Hz, 1H), 7.58 - 7.50 (m, 2H), 7.40 (td, *J* = 8.7 Hz, 1.4 Hz, 1H), 7.20 (td, *J* = 7.9 Hz, 1.5 Hz, 1H), 7.06 (d, *J* = 8.7 Hz, 1H); LC-MS (ESI); [M+H]$^{+}$: 282.0

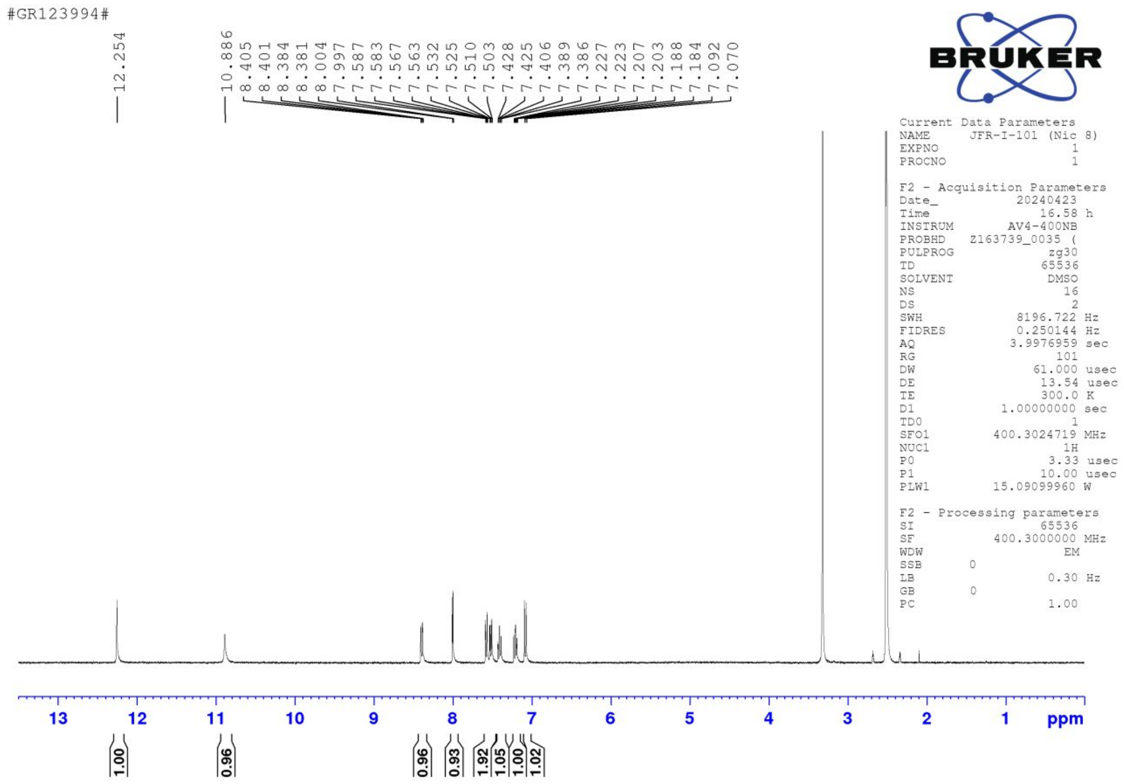

Vial:2:8
Date:25-Jun-2024
Method:C:\MassLynx\OpenLynx_Methods\ESI+_100-1250+PDA.olp
MS Method:OA_ESI+_Default

ID:nic-analogs-09-20240625-0902
Time:09:55:42
Inlet Method:OA_Default

File:nic-analogs-09-20240625-0902_COD
Description:nic 8
Instrument:ACQ-QDA#KBD6021
Detectors:Waters Acquity PDA

Printed: Tue Jun 25 10:50:57 2024

**Sample Report (continued):**

**Sample 9 nic 8 25-Jun-2024 09:55:42 File: nic-analogs-09-20240625-0902_COD**

1: MS ES+ :TIC Smooth (SG, 2x3) 7.9e+006

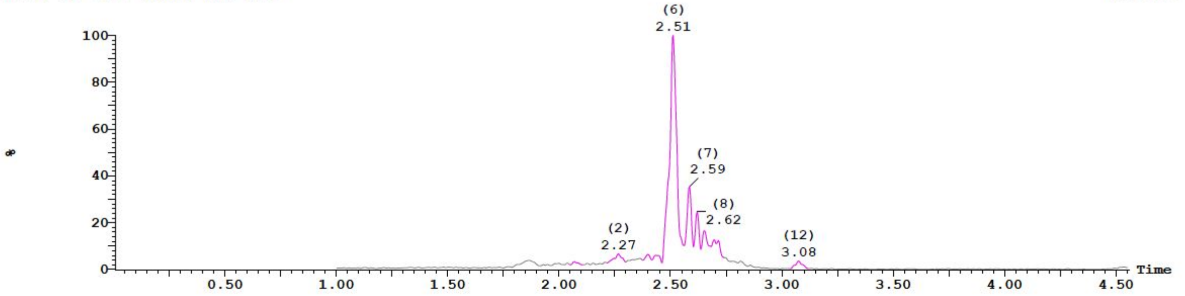


2: UV Detector: TAC: Wavelength Range: (210 - 499) 1.507e+2
Range: 1.507e+2

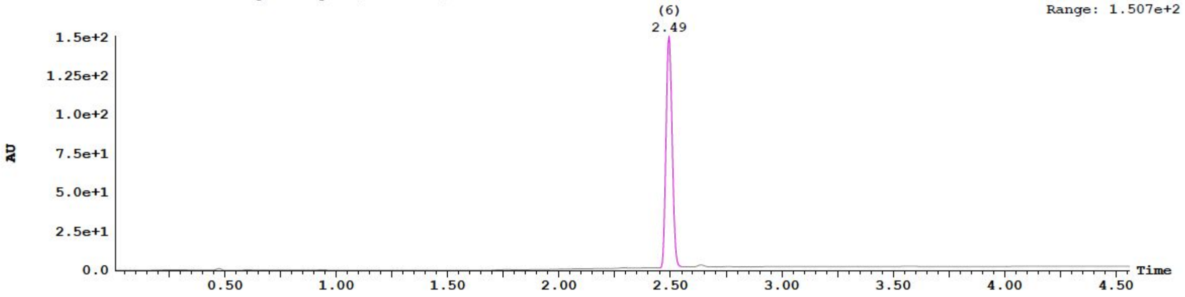


**Sample Report (continued):**

**Peak ID** 1 **Time** 2.07
1:(Time: 2.07) Combine (291:351-(261:290+352:381)) 1:MS ES+ 1.1e+004

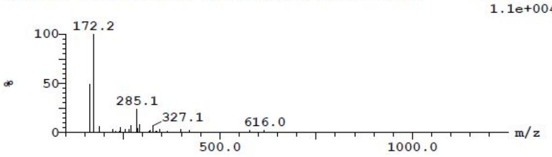


**Peak ID** 2 **Time** 2.27
2:(Time: 2.27) Combine (350:410-(320:349+411:440)) 1:MS ES+ 4.1e+004

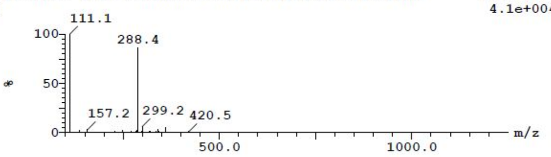


**Peak ID** 3 **Time** 2.40
3:(Time: 2.40) Combine (390:450-(360:389+451:480)) 1:MS ES+ 4.8e+004

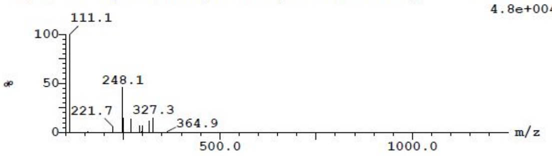


**Peak ID** 4 **Time** 2.44
4:(Time: 2.44) Combine (401:461-(371:400+462:491)) 1:MS ES+ 4.8e+005

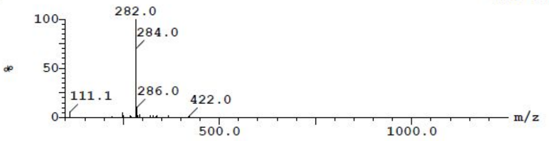


**Peak ID** 5 **Time** 2.45
5:(Time: 2.45) Combine (404:464-(374:403+465:494)) 1:MS ES+ 5.3e+005

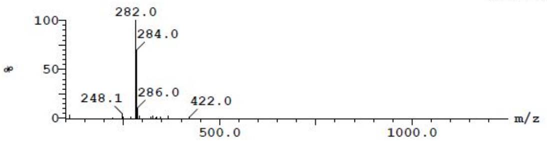


**Peak ID** 6 **Time** 2.50
6:(Time: 2.49) Combine (418:478-(388:417+479:508)) 1:MS ES+ 6.4e+005

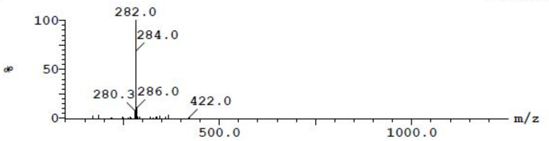

**5-chloro-2-hydroxy-N-(4-(trifluoromethyl)phenyl)benzamide (Nic-11)**[27]

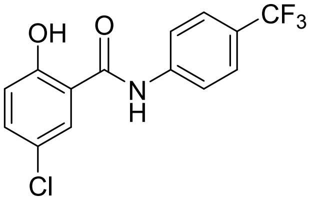

$^{1}$HNMR (400 MHz, DMSO-$d_6$) δ 11.5 (s, 1H), 10.6 (s, 1H), 7.94 (d, *J* = 8.4 Hz, 2H), 7.88 (d, *J* = 2.6 Hz, 1H), 7.74 (d, *J* = 8.6 Hz, 2H), 7.47 (dd, *J* = 8.8 Hz, 2.7 Hz, 1H), 7.03 (d, *J* = 8.7 Hz, 1H); LC-MS (ESI); [M-H]$^{-}$: 313.9

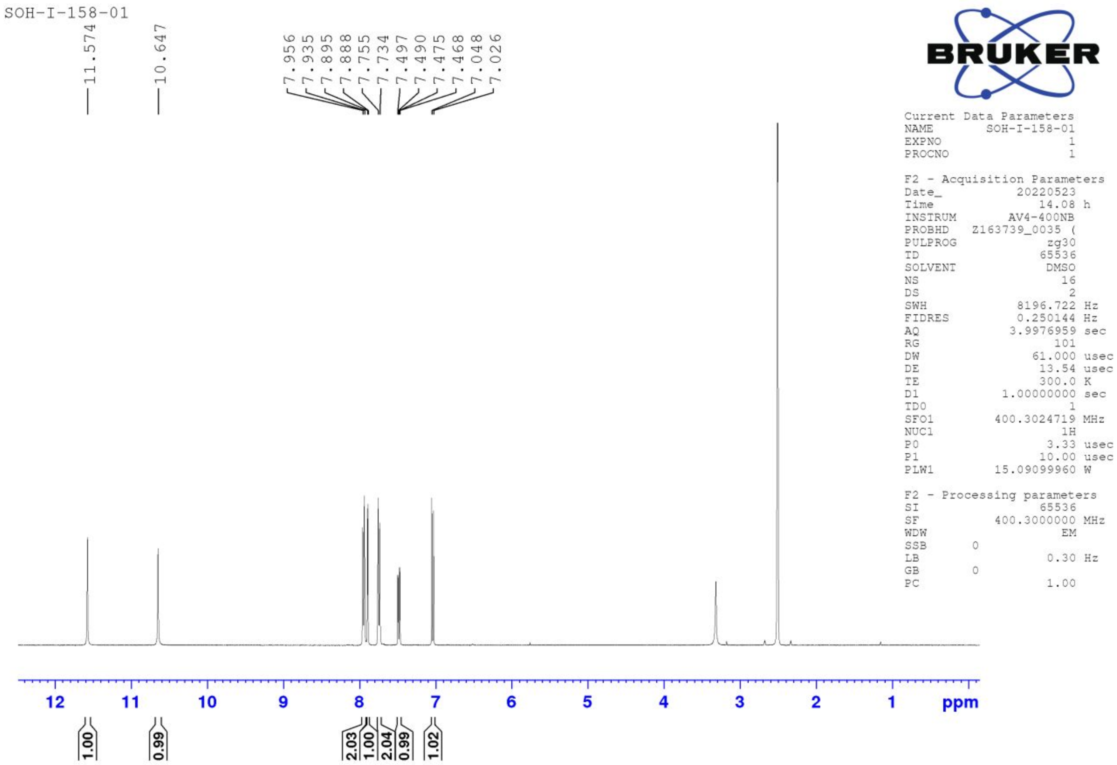

Vial:1:14
Date:19-Sep-2024
Method:C:\MassLynx\OpenLynx_Methods\ESI-_100-1250+PDA.olp
MS Method:OA_ESI-_Default

ID:mohammed210_149-4-20240919-1212
Time:12:37:07
Inlet Method:OA_Default

File:mohammed210_149-4-20240919-1212_COD
Description:Nic-11
Instrument:ACQ-QDA#KBD6021
Detectors:Waters Acquity PDA

Printed: Thu Sep 19 13:03:11 2024

**Sample Report (continued):**

**Sample 4 Nic-11 19-Sep-2024 12:37:07 File: mohammed210_149-4-20240919-1212_COD**

1: MS ES- :TIC Smooth (SG, 2x3) 4.1e+006

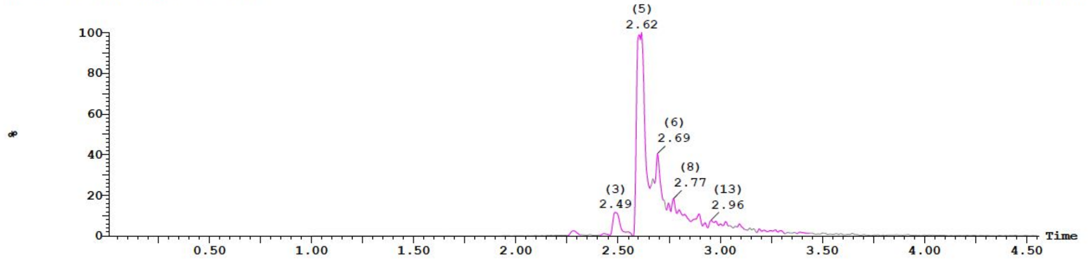


2: UV Detector: TAC: Wavelength Range: (210 - 499) 1.825e+1
Range: 1.825e+1

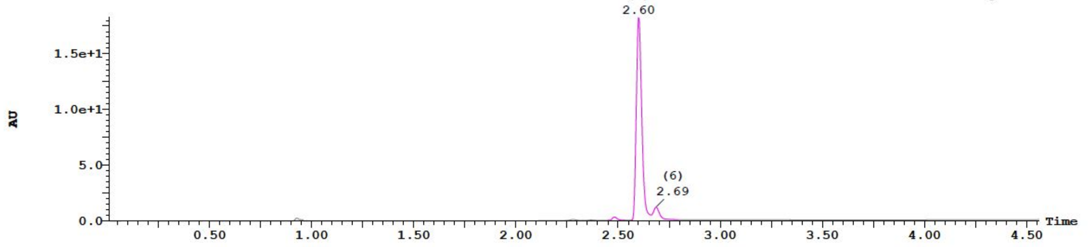


**Sample Report (continued):**

| Peak ID | Time |
|---|---|
| 5 | 2.60 |

5:(Time: 2.60) Combine (778) 2:UV Detector 3.144e-1 AU

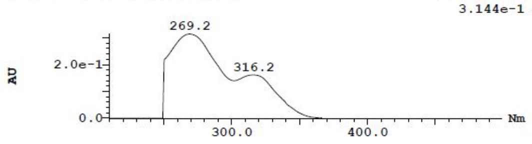


| Peak ID | Time |
|---|---|
| 6 | 2.69 |

6:(Time: 2.69) Combine (476:536-(446:475+537:566)) 1:MS ES- 7.6e+005

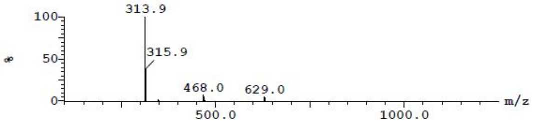


| Peak ID | Time |
|---|---|
| 6 | 2.69 |

6:(Time: 2.69) Combine (804) 2:UV Detector 4.947e-2 AU

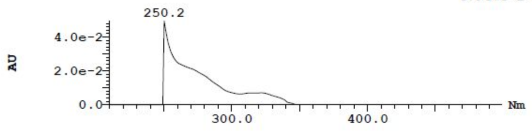


| Peak ID | Time |
|---|---|
| 7 | 2.75 |

7:(Time: 2.75) Combine (494:554-(464:493+555:584)) 1:MS ES- 5.4e+004

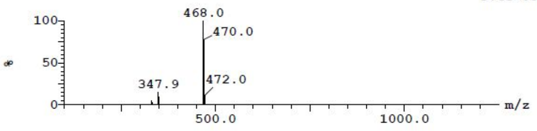


| Peak ID | Time |
|---|---|
| 8 | 2.77 |

8:(Time: 2.77) Combine (502:562-(472:501+563:592)) 1:MS ES- 5.4e+004

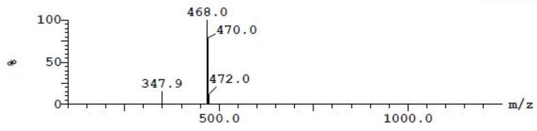


| Peak ID | Time |
|---|---|
| 9 | 2.80 |

9:(Time: 2.80) Combine (510:570-(480:509+571:600)) 1:MS ES- 7.8e+003

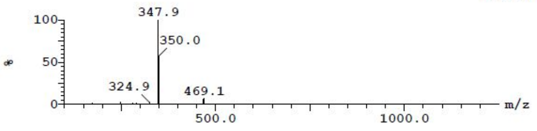

**5-chloro-2-hydroxy-N-(4-methoxyphenyl)benzamide** (**Nic-12**)[28]

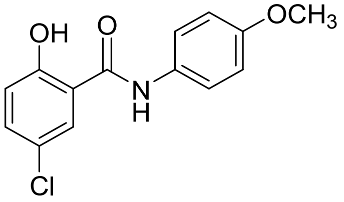

$^{1}$HNMR (400 MHz, $CD_3OD$) δ 7.98 (d, *J* = 2.6 Hz, 1H), 7.57 - 7.54 (m, 2H), 7.40 (dd, *J* = 8.8 Hz, 2.6 Hz, 1H), 6.97 - 6.94 (m, 3H), 3.82 (s, 3H); LC-MS (ESI); $[M-H]^-$: 276.0

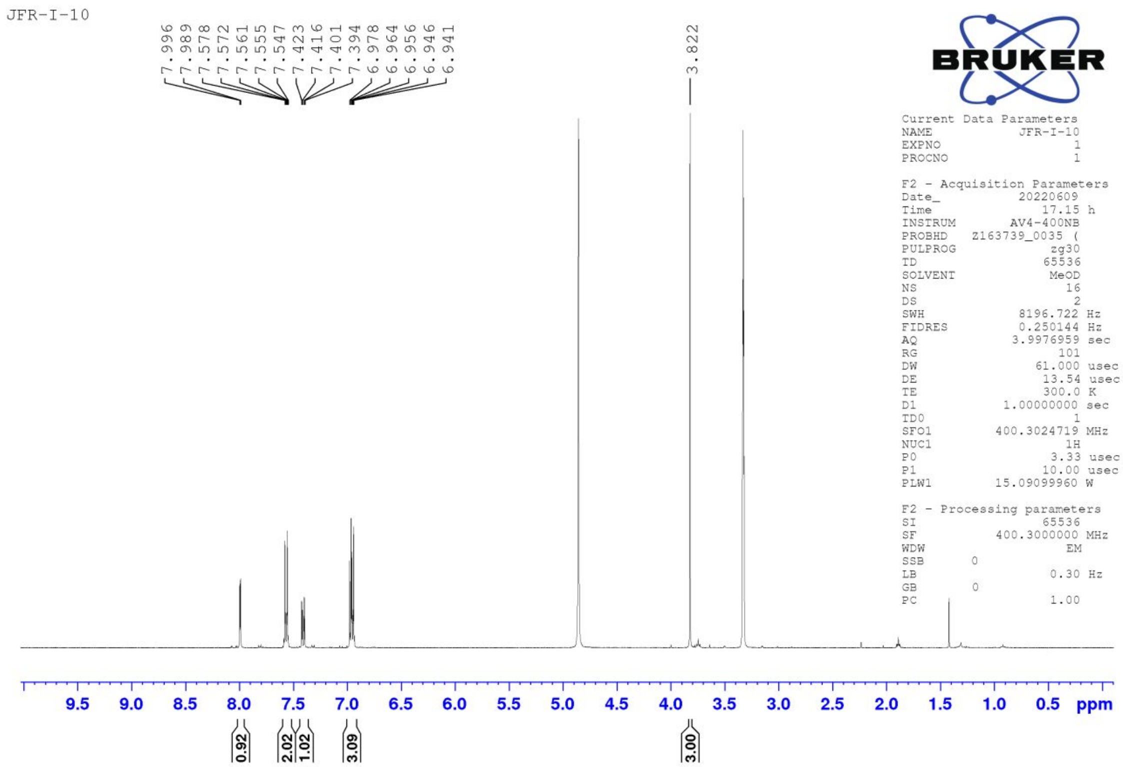

Vial:2:1
Date:16-Sep-2024
Method:C:\MassLynx\OpenLynx_Methods\ESI-_100-1250+PDA.olp
MS Method:OA_ESI-_Default

ID:mohammed210_147-06-20240916-1113
Time:12:41:58
Inlet Method:OA_Default

File:mohammed210_147-06-20240916-1113_COD
Description:NIC-12
Instrument:ACQ-QDA#KBD6021
Detectors:Waters Acquity PDA

Printed: Mon Sep 16 13:31:21 2024

**Sample Report (continued):**

**Sample 6 NIC-12 16-Sep-2024 12:41:58 File: mohammed210_147-06-20240916-1113_COD**

1: MS ES- :TIC Smooth (SG, 2x3) 2.5e+006

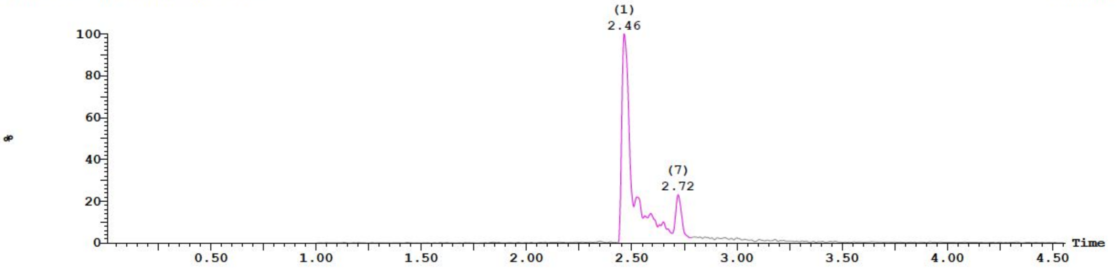


2: UV Detector: TAC: Wavelength Range: (210 - 499) 2.629e+1
Range: 2.629e+1

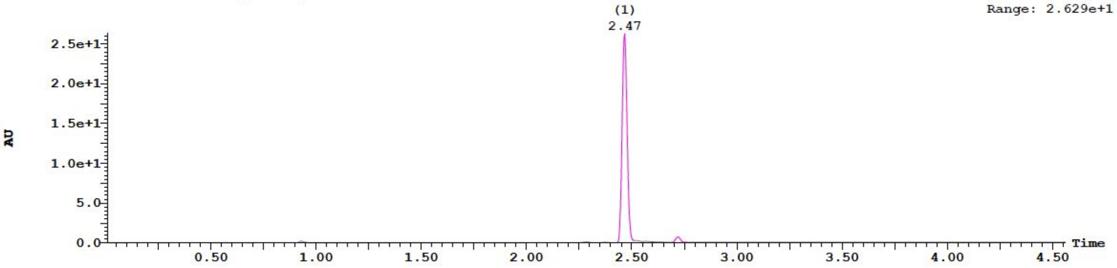


**Sample Report (continued):**

**Peak ID** **Time**
1 2.46
1:(Time: 2.47) Combine (410:470-(380:409+471:500)) 1:MS ES- 3.0e+005

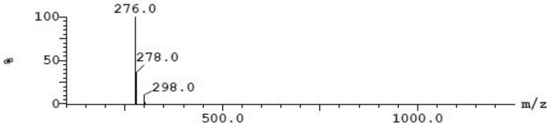


**Peak ID** **Time**
1 2.46
1:(Time: 2.47) Combine (738) 2:UV Detector 3.278e-1 AU

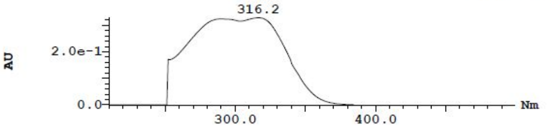


**Peak ID** **Time**
2 2.53
2:(Time: 2.53) Combine (428:488-(398:427+489:518)) 1:MS ES- 3.8e+005

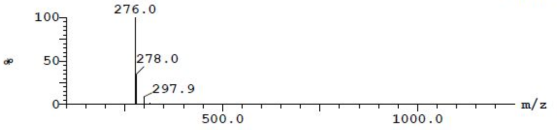


**Peak ID** **Time**
3 2.56
3:(Time: 2.56) Combine (439:499-(409:438+500:529)) 1:MS ES- 2.5e+005

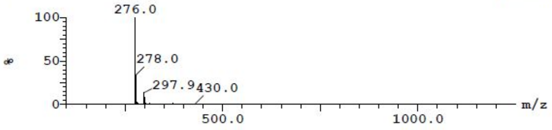


**Peak ID** **Time**
4 2.59
4:(Time: 2.59) Combine (447:507-(417:446+508:537)) 1:MS ES- 3.4e+004

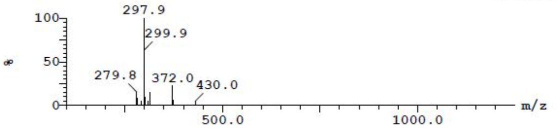


**Peak ID** **Time**
5 2.65
5:(Time: 2.65) Combine (465:525-(435:464+526:555)) 1:MS ES- 3.0e+004

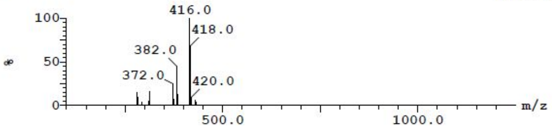

**5-chloro-2-hydroxy-N-(p-tolyl)benzamide** (**Nic 13**)[29]

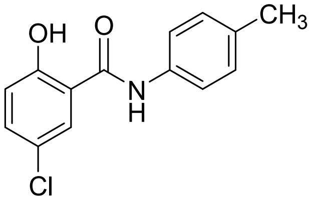

$^1$HNMR (300 MHz, DMSO-$d_6$) δ 11.92 (s, 1H), 11.34 (s, 1H), 7.97 (d, $J$ = 2.6 Hz, 1H), 7.57 (d, $J$ = 8.3 Hz, 2H), 7.46 (dd, $J$ = 8.7 Hz, 2.6 Hz, 1H), 8.18 (d, $J$ = 8.3 Hz, 2H), 7.0 (d, $J$ = 7.2 Hz, 1H), 2.29 (s, 3H); LC-MS (ESI); [M-H]$^-$: 262.1

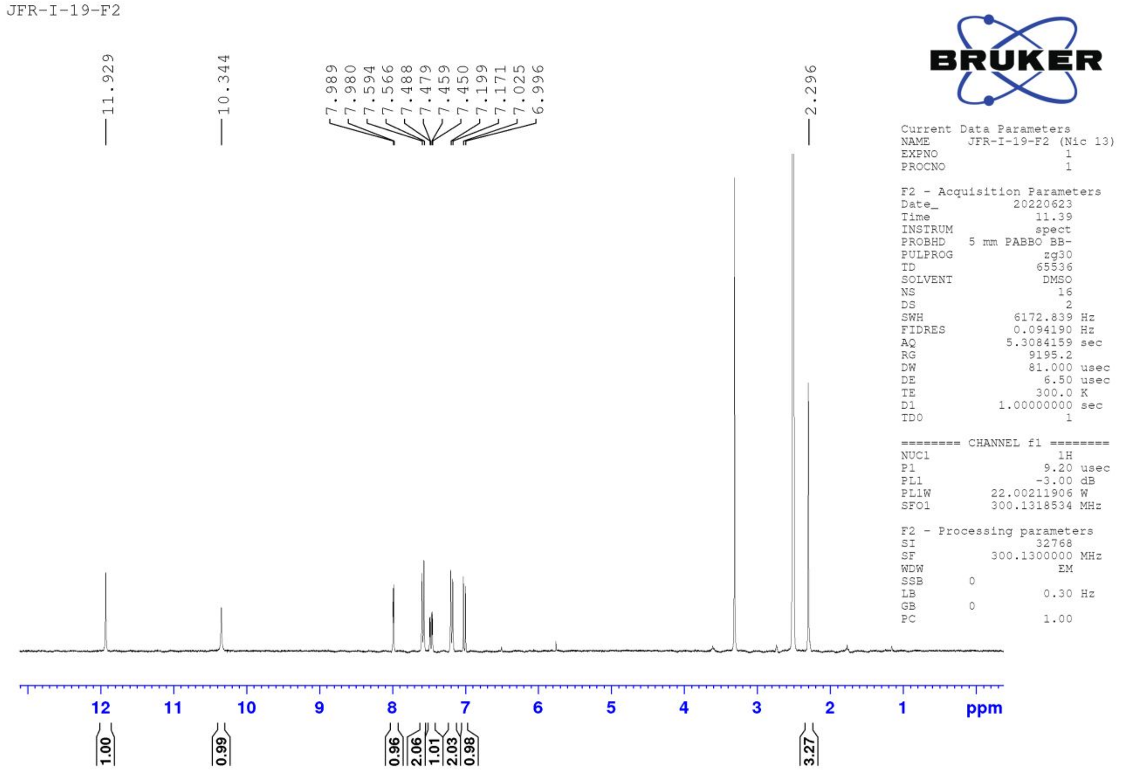

**Openlynx Report – mohammed210**

Vial:1:10
Date:26-Jun-2024
Method:C:\MassLynx\OpenLynx_Methods\ESI+_100-1250+PDA.olp
MS Method:OA_ESI+_Default

ID:mohammed210_119-04-20240626-1706
Time:17:28:24
Inlet Method:OA_Default

File:mohammed210_119-04-20240626-1706_COD
Description:nic 13
Instrument:ACQ-QDA#KBD6021
Detectors:Waters Acquity PDA

Printed: Wed Jun 26 18:17:45 2024

**Sample Report (continued):**

**Sample 4 nic 13 26-Jun-2024 17:28:24 File: mohammed210_119-04-20240626-1706_COD**

1: MS ES+ :TIC Smooth (SG, 2x3) 1.8e+007

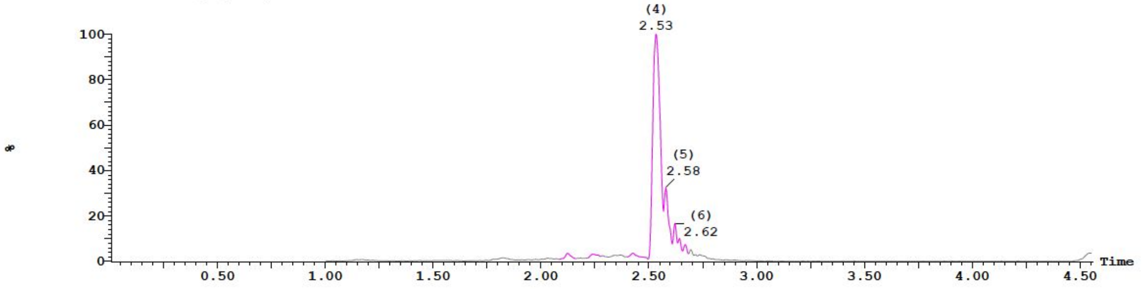


2: UV Detector: TAC: Wavelength Range: (210 - 499) 2.154e+2
Range: 2.154e+2

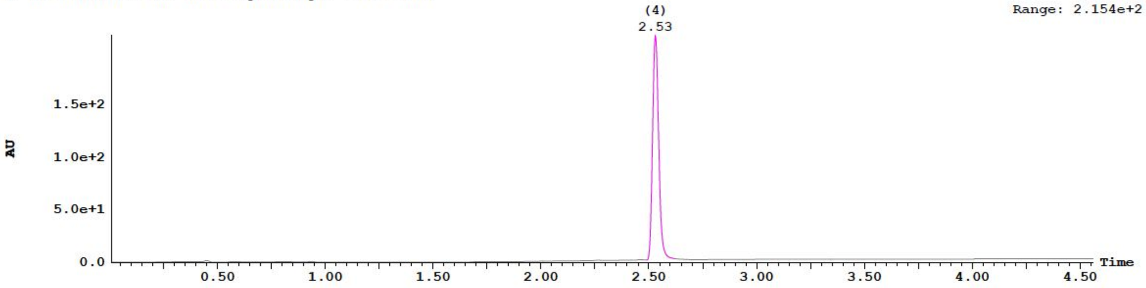


**Sample Report (continued):**

**Peak ID** 1 **Time** 2.12
1:(Time: 2.12) Combine (307:367-(277:306+368:397)) 1:MS ES+ 4.1e+004

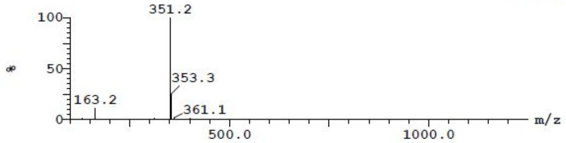


**Peak ID** 2 **Time** 2.24
2:(Time: 2.24) Combine (343:403-(313:342+404:433)) 1:MS ES+ 4.0e+004

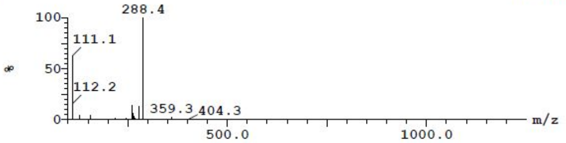


**Peak ID** 3 **Time** 2.43
3:(Time: 2.43) Combine (398:458-(368:397+459:488)) 1:MS ES+ 5.1e+004

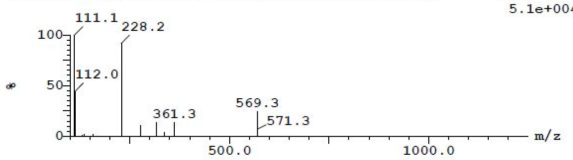


**Peak ID** 4 **Time** 2.53
4:(Time: 2.53) Combine (429:489-(399:428+490:519)) 1:MS ES+ 2.6e+006

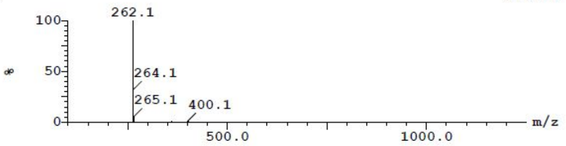


**Peak ID** 4 **Time** 2.53
4:(Time: 2.53) Combine (757) 2:UV Detector 2.457 AU

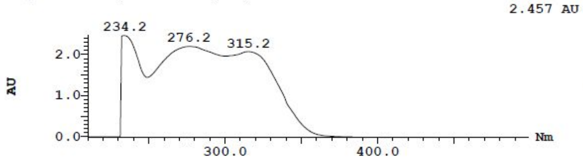


**Peak ID** 5 **Time** 2.58
5:(Time: 2.58) Combine (444:504-(414:443+505:534)) 1:MS ES+ 2.9e+006

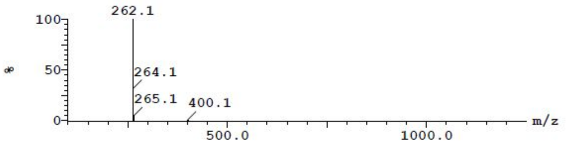

**N-(2-chloro-4-nitrophenyl)benzamide (Nic-14)**[30]

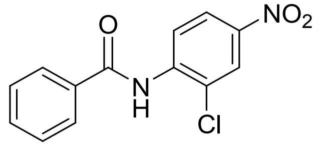

$^1$HNMR (300 MHz, DMSO-$d_6$) δ 10.32 (s, 1H), 8.42 (d, *J* = 2.5 Hz, 1H), 8.27 (dd, *J* = 8.9 Hz, 2.5 Hz, 1H), 8.08 -7.99 (m, 3H), 7.66 -7.55 (m, 3H); LC-MS (ESI); $[M+H]^+$: 277.1

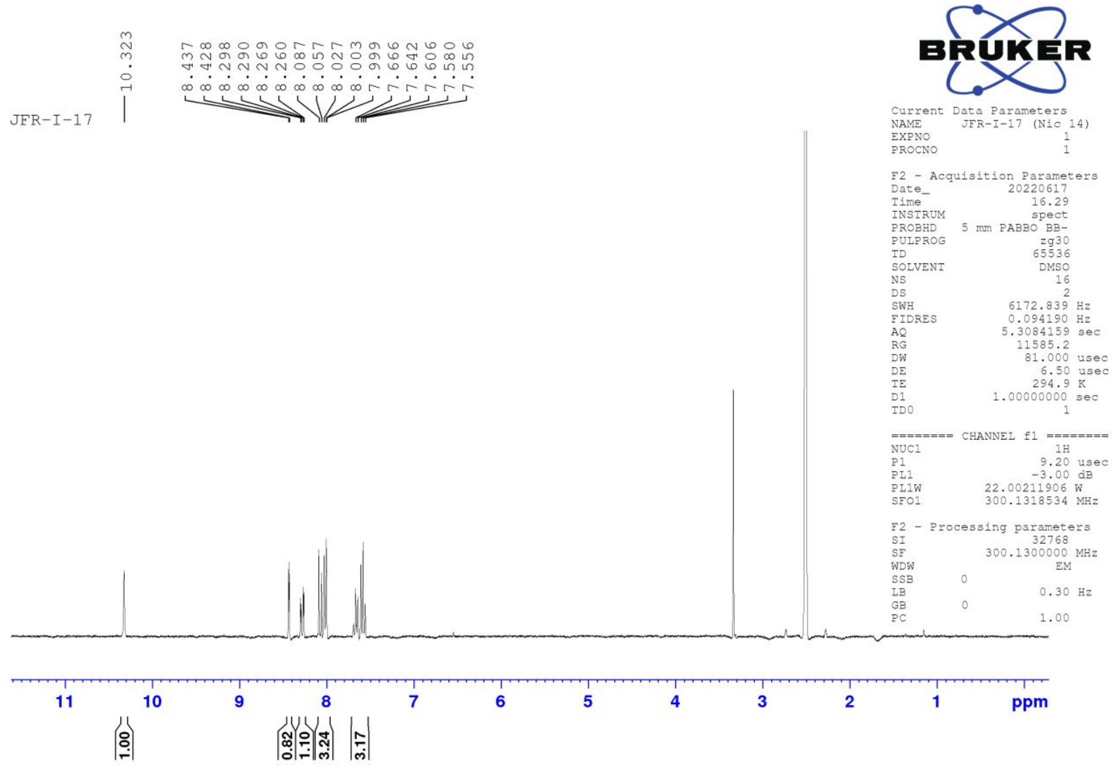

Vial:1:11
Date:26-Jun-2024
Method:C:\MassLynx\OpenLynx_Methods\ESI+_100-1250+PDA.olp
MS Method:OA_ESI+_Default

ID:mohammed210_119-05-20240626-1706
Time:17:34:19
Inlet Method:OA_Default

File:mohammed210_119-05-20240626-1706_COD
Description:nic 14
Instrument:ACQ-QDA#KBD6021
Detectors:Waters Acquity PDA

Printed: Wed Jun 26 18:17:45 2024

**Sample Report (continued):**

**Sample 5 nic 14 26-Jun-2024 17:34:19 File: mohammed210_119-05-20240626-1706_COD**

1: MS ES+ :TIC Smooth (SG, 2x3) 3.1e+006

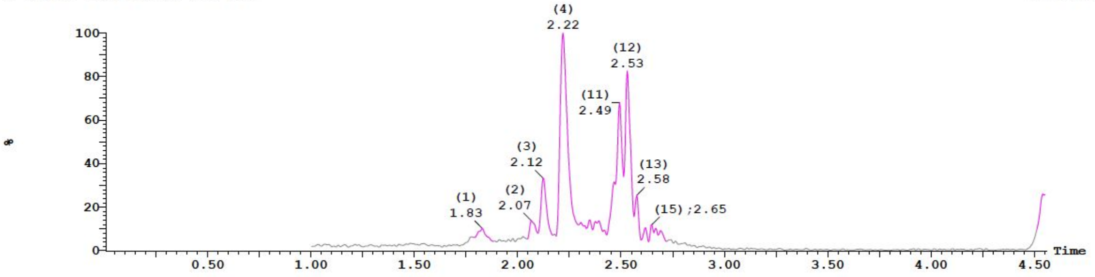


2: UV Detector: TAC: Wavelength Range: (210 - 499) 3.232e+2 Range: 3.232e+2

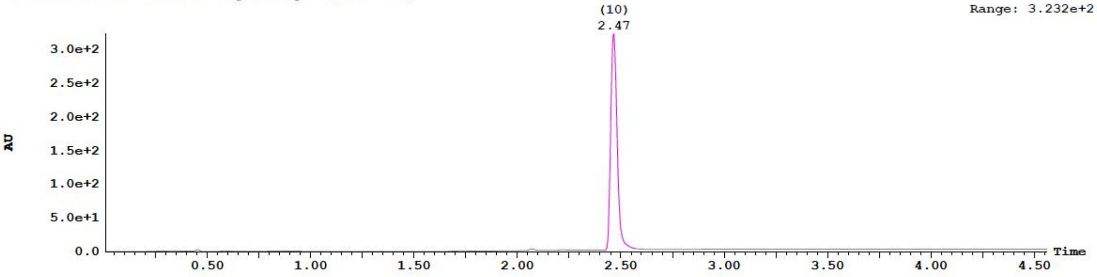


**Sample Report (continued):**

**Peak ID** 7 **Time** 2.38
7:(Time: 2.38) Combine (383:443-(353:382+444:473)) 1:MS ES+ 7.6e+004

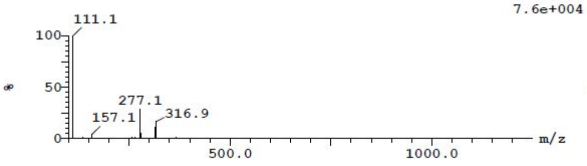


**Peak ID** 8 **Time** 2.39
8:(Time: 2.39) Combine (388:448-(358:387+449:478)) 1:MS ES+ 7.7e+004

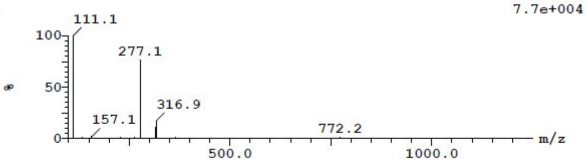


**Peak ID** 9 **Time** 2.42
9:(Time: 2.42) Combine (396:456-(366:395+457:486)) 1:MS ES+ 7.4e+004

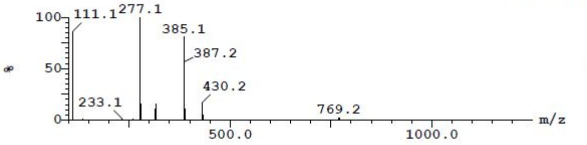


**Peak ID** 10 **Time** 2.47
10:(Time: 2.47) Combine (410:470-(380:409+471:500)) 1:MS ES+ 1.8e+005

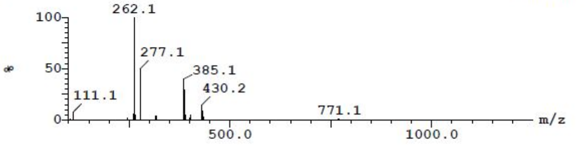


**Peak ID** 10 **Time** 2.47
10:(Time: 2.47) Combine (738) 2:UV Detector 2.841 AU

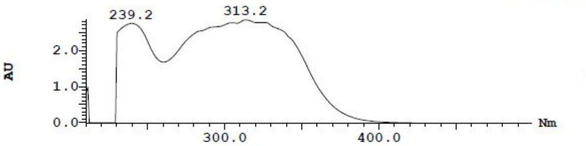


**Peak ID** 11 **Time** 2.49
11:(Time: 2.49) Combine (418:478-(388:417+479:508)) 1:MS ES+ 2.0e+005

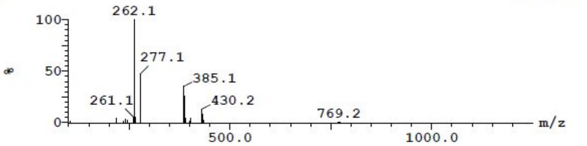

**5-chloro-N-(4-chlorophenyl)-2-hydroxybenzamide** (**Nic-15**)[31]

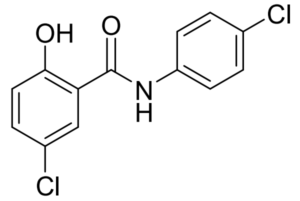


$^{1}$HNMR (400 MHz, DMSO-$d_6$) δ 11.69 (s, 1H), 10.48 (s, 1H), 7.91 (d, $J$ = 2.6 Hz, 1H), 7.74 (d, $J$ = 8.8 Hz, 2H), 7.48 - 7.42 (m, 3H), 7.01 (d, $J$ = 8.8 Hz, 1H), ; LC-MS (ESI); [M+H]$^{+}$: 282.1

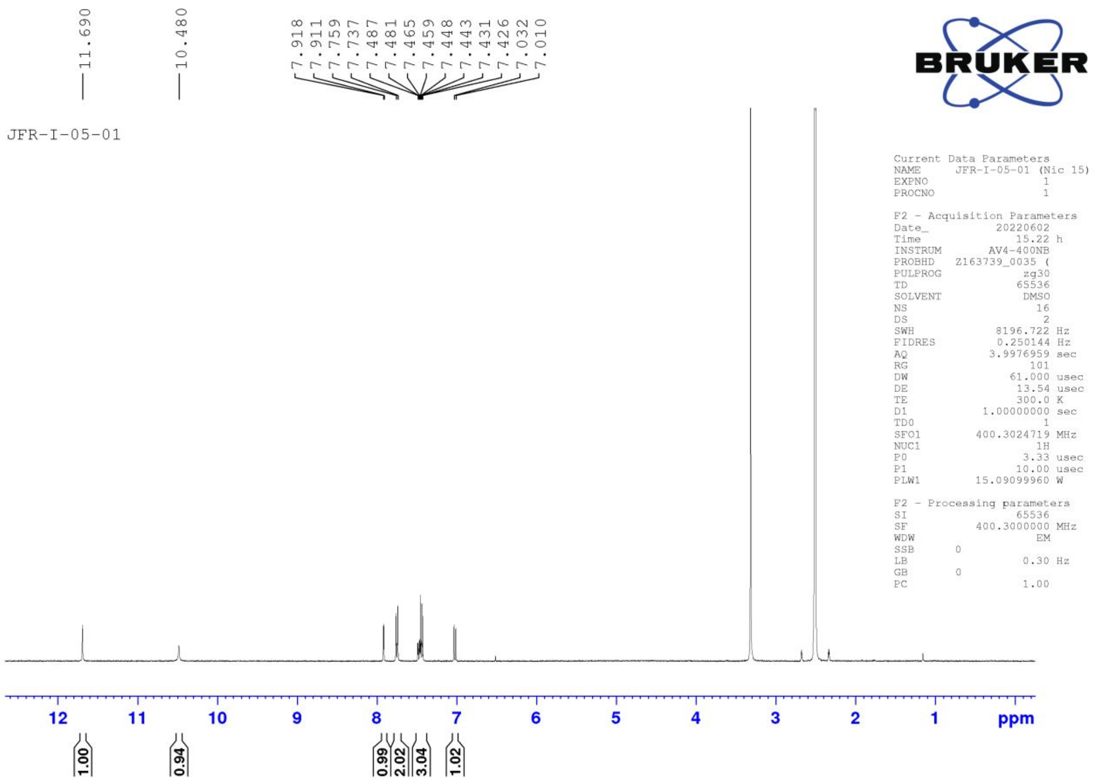

Vial:2:12 | ID:nic-analogs-13-20240625-0902 | File:nic-analogs-13-20240625-0902_COD
Date:25-Jun-2024 | Time:10:19:24 | Description:nic 15
Method:C:\MassLynx\OpenLynx_Methods\ESI+_100-1250+PDA.olp | Instrument:ACQ-QDA#KBD6021
MS Method:OA_ESI+_Default | Inlet Method:OA_Default | Detectors:Waters Acquity PDA

Printed: Tue Jun 25 10:50:57 2024

**Sample Report (continued):**

**Sample 13 nic 15 25-Jun-2024 10:19:24 File: nic-analogs-13-20240625-0902_COD**

1: MS ES+ :TIC Smooth (SG, 2x3) 8.8e+006

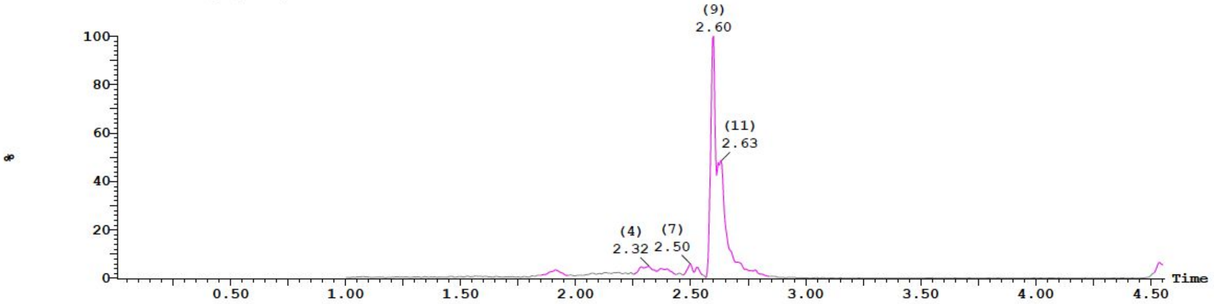


2: UV Detector: TAC: Wavelength Range: (210 - 499) 2.372e+2
Range: 2.372e+2

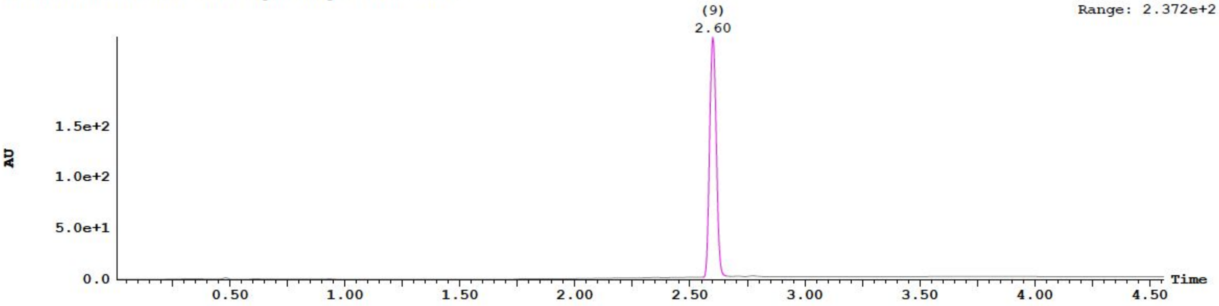


**Sample Report (continued):**

**Peak ID 7 Time 2.50**
7:(Time: 2.50) Combine (420:480-(390:419+481:510)) 1:MS ES+ 3.6e+004

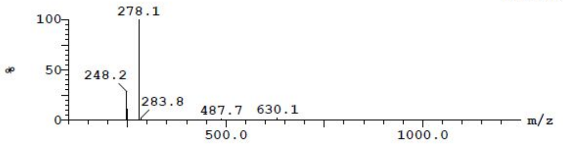


**Peak ID 8 Time 2.53**
8:(Time: 2.53) Combine (429:489-(399:428+490:519)) 1:MS ES+ 3.9e+005

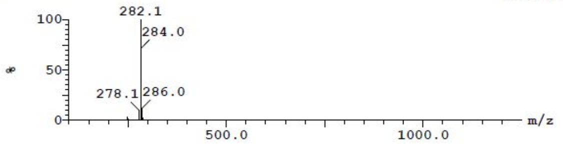


**Peak ID 9 Time 2.60**
9:(Time: 2.60) Combine (450:510-(420:449+511:540)) 1:MS ES+ 8.1e+005

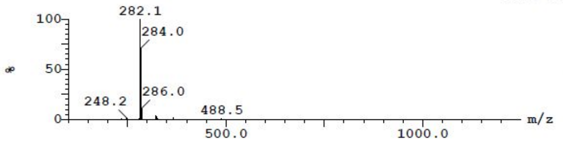


**Peak ID 9 Time 2.60**
9:(Time: 2.60) Combine (778) 2:UV Detector 2.463 AU

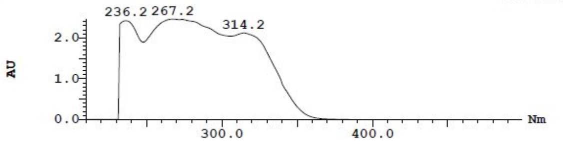


**Peak ID 10 Time 2.62**
10:(Time: 2.62) Combine (456:516-(426:455+517:546)) 1:MS ES+ 8.4e+005

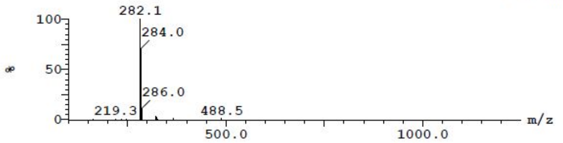


**Peak ID 11 Time 2.63**
11:(Time: 2.63) Combine (460:520-(430:459+521:550)) 1:MS ES+ 8.5e+005

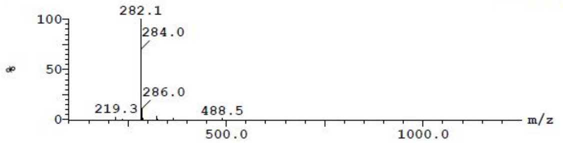

**5-chloro-N-(4-cyanophenyl)-2-hydroxybenzamide** (**Nic-20**)[32]

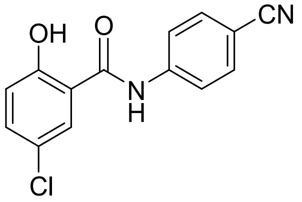


$^{1}$HNMR (400 MHz, DMSO-$d_6$) δ 11.48 (s, 1H), 10.68 (s, 1H), 7.93 - 7.91 (m, 2H), 7.85 - 7.82 (m, 3H), 7.47 (dd, $J$ = 8.8 Hz, 2.7 Hz, 1H), 7.03 (d, $J$ = 8.8 Hz, 1H); LC-MS (ESI); [M-H]$^-$: 271.0

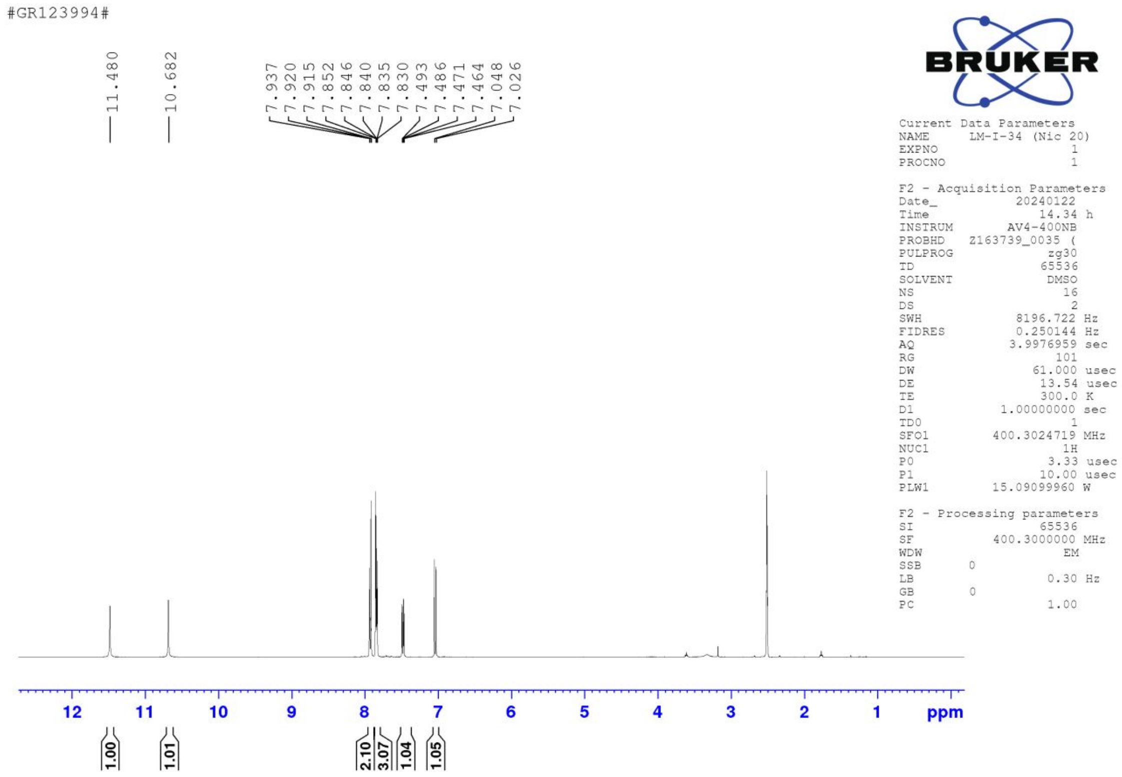

**Openlynx Report – mohammed210**

Vial:1:15
Date:19-Sep-2024
Method:C:\MassLynx\OpenLynx_Methods\ESI-_100-1250+PDA.olp
MS Method:OA_ESI-_Default

ID:mohammed210_149-5-20240919-1212
Time:12:43:05
Inlet Method:OA_Default

File:mohammed210_149-5-20240919-1212_COD
Description:Nic-20
Instrument:ACQ-QDA#KBD6021
Detectors:Waters Acquity PDA

Printed: Thu Sep 19 13:03:11 2024

**Sample Report (continued):**

**Sample 5 Nic-20 19-Sep-2024 12:43:05 File: mohammed210_149-5-20240919-1212_COD**

1: MS ES- :TIC Smooth (SG, 2x3) 1.6e+006

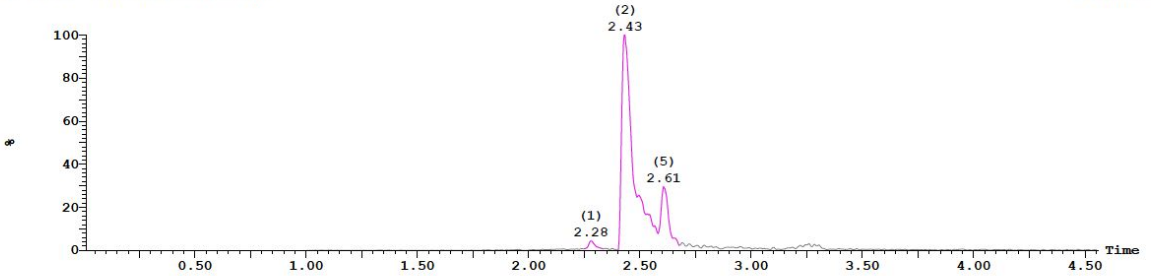


2: UV Detector: TAC: Wavelength Range: (210 - 499) 6.148
Range: 6.148

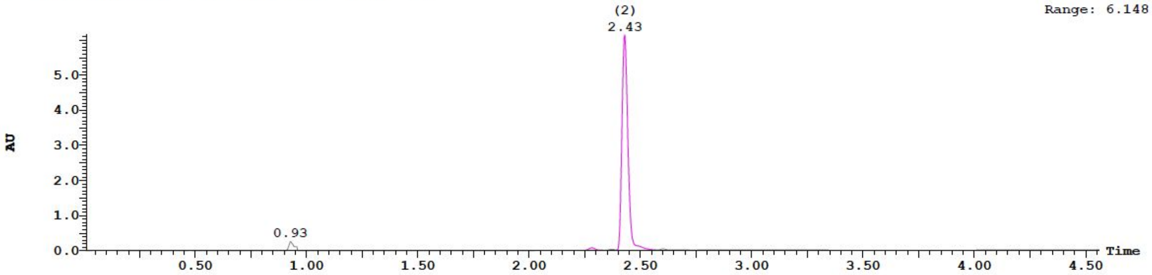


**Sample Report (continued):**

**Peak ID** 1 **Time** 2.28

1:(Time: 2.28) Combine (354:414-(324:353+415:444)) 1:MS ES- 7.3e+003

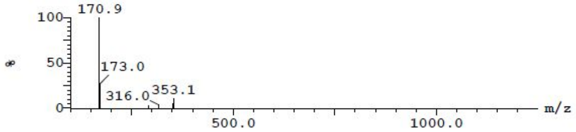


**Peak ID** 1 **Time** 2.28

1:(Time: 2.28) Combine (682) 2:UV Detector 7.597e-3 AU

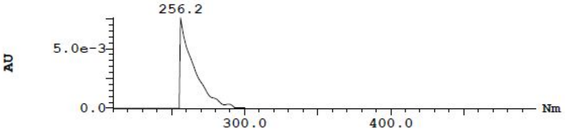


**Peak ID** 2 **Time** 2.43

2:(Time: 2.43) Combine (399:459-(369:398+460:489)) 1:MS ES- 2.4e+005

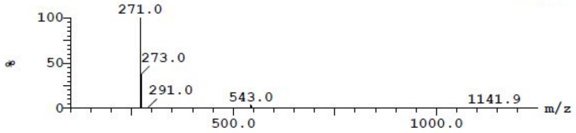


**Peak ID** 2 **Time** 2.43

2:(Time: 2.43) Combine (727) 2:UV Detector 1.126e-1 AU

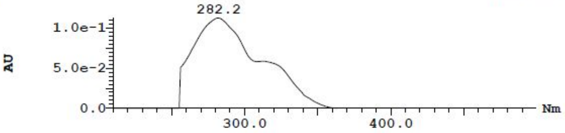


**Peak ID** 3 **Time** 2.50

3:(Time: 2.50) Combine (419:479-(389:418+480:509)) 1:MS ES- 3.1e+005

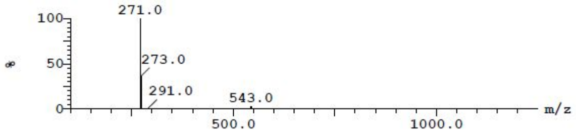


**Peak ID** 3 **Time** 2.50

3:(Time: 2.48) Combine (743) 2:UV Detector 5.977e-3 AU

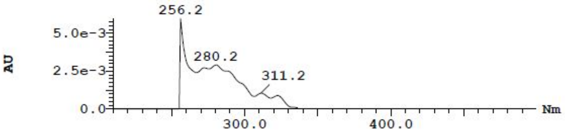

**5-chloro-N-(3-chloro-4-nitrophenyl)-2-hydroxybenzamide** (**Nic-22**)[33]

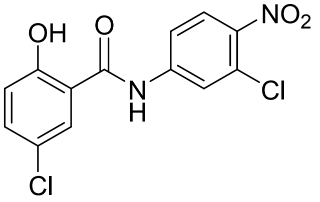


$^{1}$HNMR (400 MHz, $CD_3OD$) δ 8.20 (d, *J* = 2.2 Hz, 1H), 8.05 (d, *J* = 8.9Hz, 1H), 8.0 (d, *J* = 2.6 Hz, 1H), 7.81(dd, *J* = 8.9 Hz, 2.2 Hz, 1H), 7.81(dd, *J* = 8.9 Hz, 2.2 Hz, 1H), 6.9 (d, *J* = 8.8 Hz, 1H); LC-MS (ESI); $[M-H]^-$: 324.9

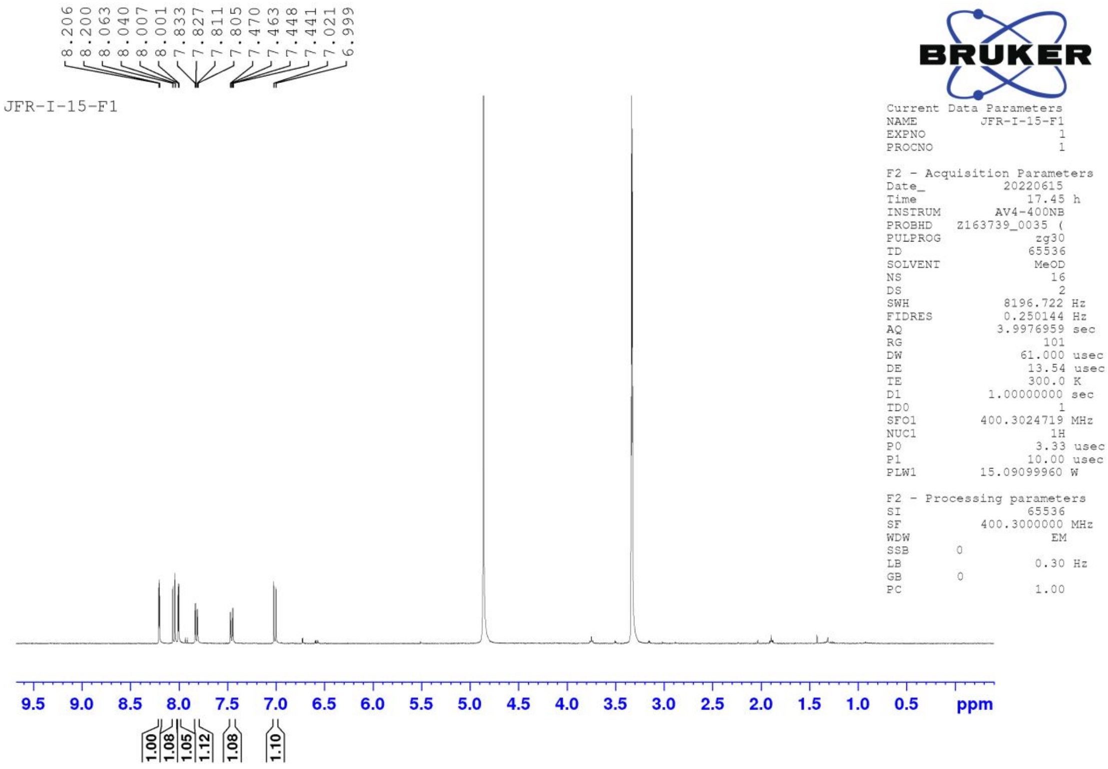

Vial:2:7
Date:16-Sep-2024
Method:C:\MassLynx\OpenLynx_Methods\ESI-_100-1250+PDA.olp
MS Method:OA_ESI-_Default

ID:mohammed210_147-12-20240916-1113
Time:13:17:40
Inlet Method:OA_Default

File:mohammed210_147-12-20240916-1113_COD
Description:NIC-22
Instrument:ACQ-QDA#KBD6021
Detectors:Waters Acquity PDA

Printed: Mon Sep 16 13:31:21 2024

**Sample Report (continued):**

**Sample 12 NIC-22 16-Sep-2024 13:17:40 File: mohammed210_147-12-20240916-1113_COD**

1: MS ES- :TIC Smooth (SG, 2x3) 1.6e+006

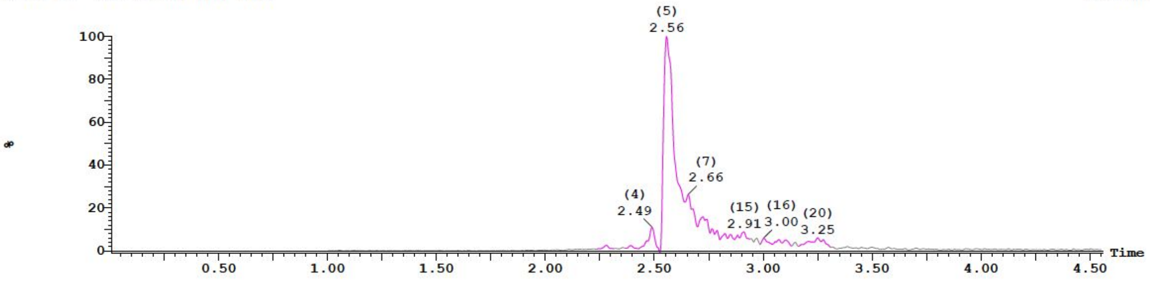


2: UV Detector: TAC: Wavelength Range: (210 - 499) 1.319e+1

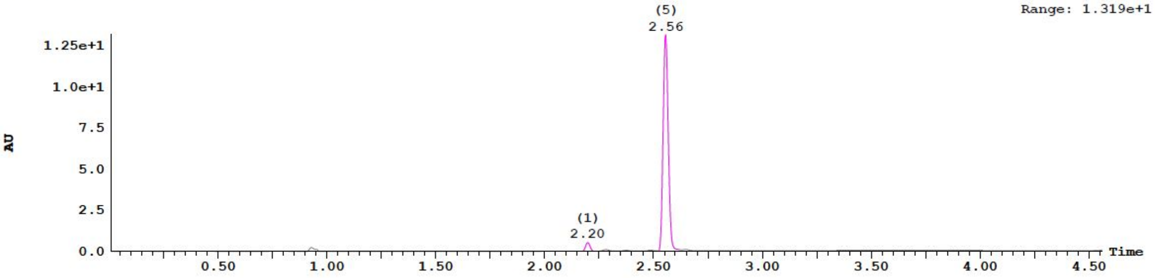


**Sample Report (continued):**

**Peak ID** 1 **Time** 2.20
1:(Time: 2.20) Combine (329:389-(299:328+390:419)) 1:MS ES- 2.2e+003

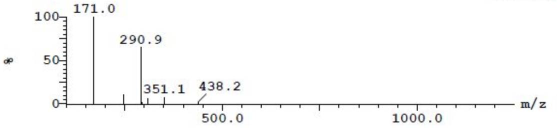


**Peak ID** 1 **Time** 2.20
1:(Time: 2.20) Combine (657) 2:UV Detector 7.711e-3 AU

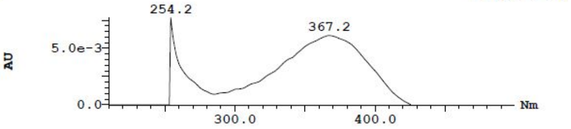


**Peak ID** 2 **Time** 2.28
2:(Time: 2.28) Combine (354:414-(324:353+415:444)) 1:MS ES- 2.3e+003

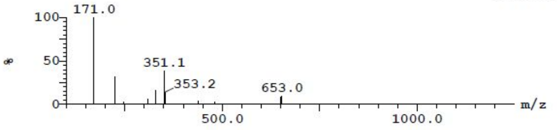


**Peak ID** 3 **Time** 2.39
3:(Time: 2.39) Combine (388:448-(358:387+449:478)) 1:MS ES- 4.6e+003

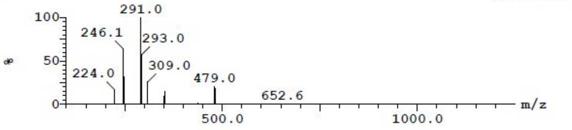


**Peak ID** 4 **Time** 2.49
4:(Time: 2.49) Combine (417:477-(387:416+478:507)) 1:MS ES- 6.0e+004

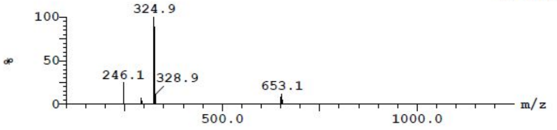


**Peak ID** 5 **Time** 2.56
5:(Time: 2.56) Combine (437:497-(407:436+498:527)) 1:MS ES- 1.8e+005

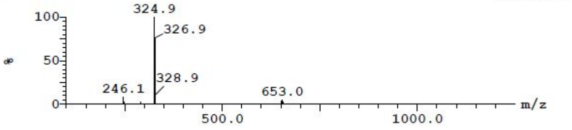

**N-(2,4-dichlorophenyl)-2-hydroxybenzamide** (**Nic-40**)[34]

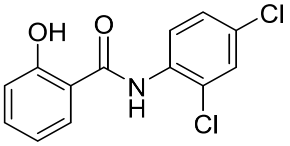


$^{1}$HNMR (400 MHz, DMSO-$d_6$) δ 11.95 (s, 1H), 10.95 (s, 1H), 8.45 (d, *J* = 8.8 Hz, 1H), 8.03 (dd, *J* = 7.9Hz, 1.7 Hz, 1H), 7.73 (d, *J* = 2.4 Hz, 1H), 7.50 - 7.44 (m, 2H), 7.06 - 6.99 (m, 2H); LC-MS (ESI); $[M+H]^+$: 282.0

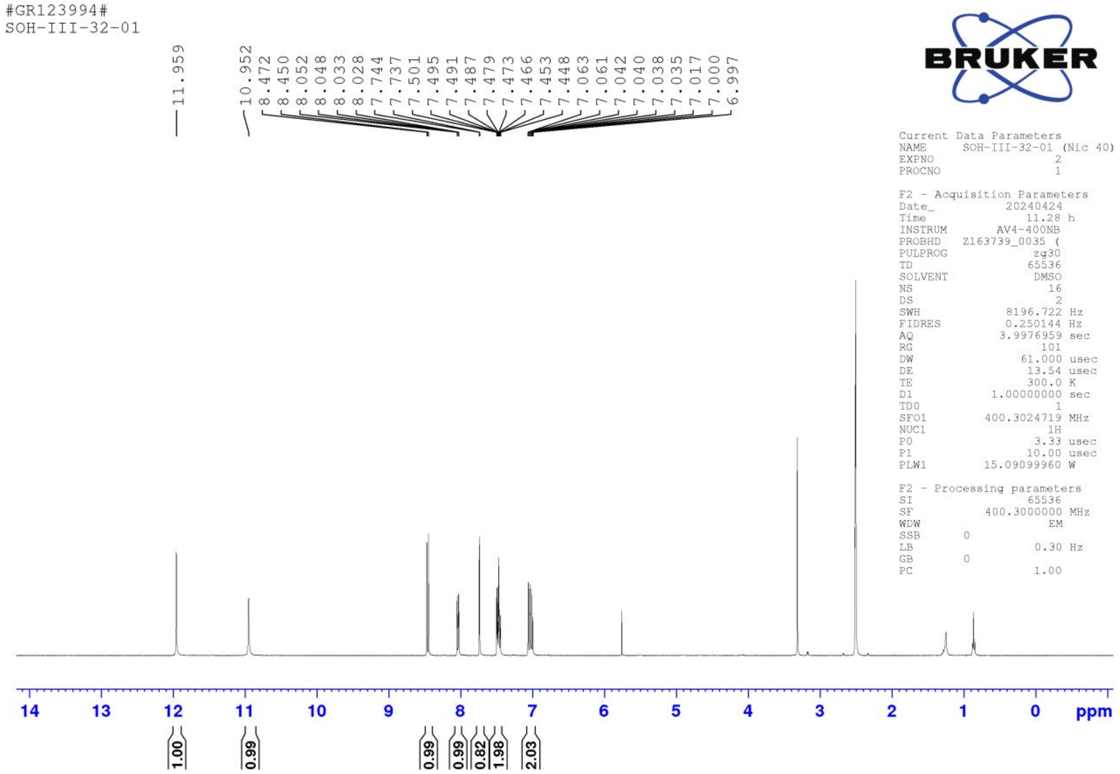

**Openlynx Report – mohammed210**

Vial:2:20
Date:25-Jun-2024
Method:C:\MassLynx\OpenLynx_Methods\ESI+_100-1250+PDA.olp
MS Method:OA_ESI+_Default

ID:mohammed210_116-02-20240625-1643
Time:16:54:49
Inlet Method:OA_Default

File:mohammed210_116-02-20240625-1643_COD
Description:Nic 40
Instrument:ACQ-QDA#KBD6021
Detectors:Waters Acquity PDA

Printed: Tue Jun 25 18:38:24 2024

**Sample Report (continued):**

**Sample 2 Nic 40 25-Jun-2024 16:54:49 File: mohammed210_116-02-20240625-1643_COD**

1: MS ES+ :TIC Smooth (SG, 2x3) 8.0e+006

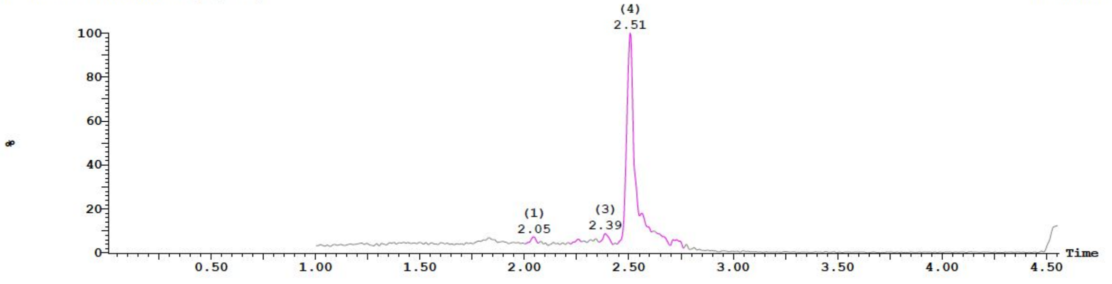


2: UV Detector: TAC: Wavelength Range: (210 - 499) 8.541e+1
Range: 8.541e+1

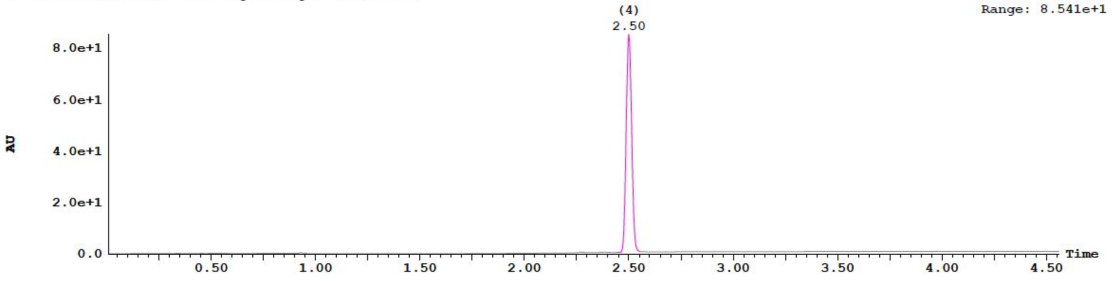


**Sample Report (continued):**

**Peak ID** 1 **Time** 2.05
1:(Time: 2.05) Combine (284:344-(254:283+345:374)) 1:MS ES+ 3.4e+004

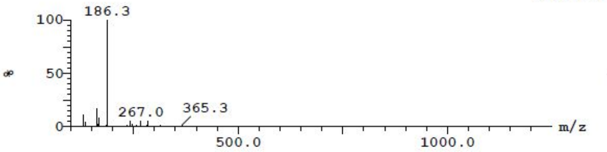


**Peak ID** 2 **Time** 2.26
2:(Time: 2.26) Combine (347:407-(317:346+408:437)) 1:MS ES+ 5.4e+004

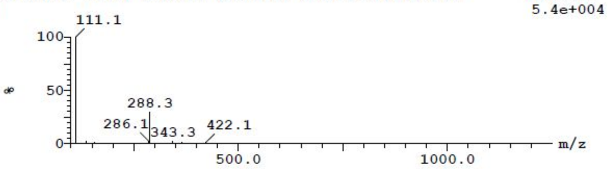


**Peak ID** 3 **Time** 2.39
3:(Time: 2.39) Combine (386:446-(356:385+447:476)) 1:MS ES+ 5.7e+004

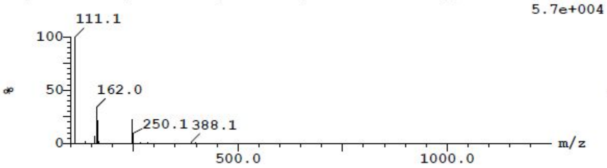


**Peak ID** 4 **Time** 2.51
4:(Time: 2.50) Combine (420:480-(390:419+481:510)) 1:MS ES+ 7.4e+005

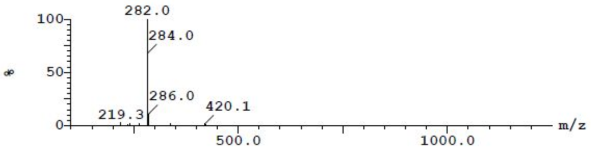


**Peak ID** 4 **Time** 2.51
4:(Time: 2.50) Combine (748) 2:UV Detector 1.343 AU

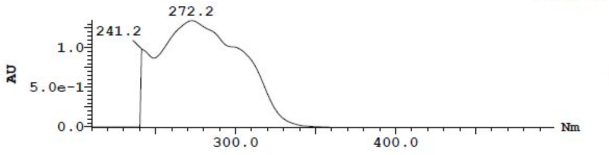


**Peak ID** 5 **Time** 2.56
5:(Time: 2.56) Combine (439:499-(409:438+500:529)) 1:MS ES+ 8.0e+005

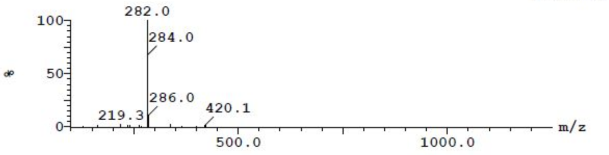

**N-(2-chlorophenyl)-2-hydroxybenzamide** (**Nic-41**)[35]

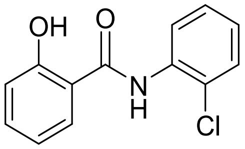

$^1$HNMR (400 MHz, DMSO-$d_6$) δ 11.93 (s, 1H), 10.89 (s, 1H), 8.40 (dd, *J* = 8.2 Hz, 1.5 Hz, 1H), 8.04 (dd, *J* = 7.9 Hz, 1.7 Hz, 1H), 7.56 (dd, *J* = 8.0 Hz, 1.4 Hz, 1H), 7.48 - 7.37 (m, 2H), 7.18 (td, *J* = 7.9 Hz, 1.6 Hz, 1H), 7.05 - 6.99 (m, 2H); LC-MS (ESI); [M+H]$^+$: 248.1

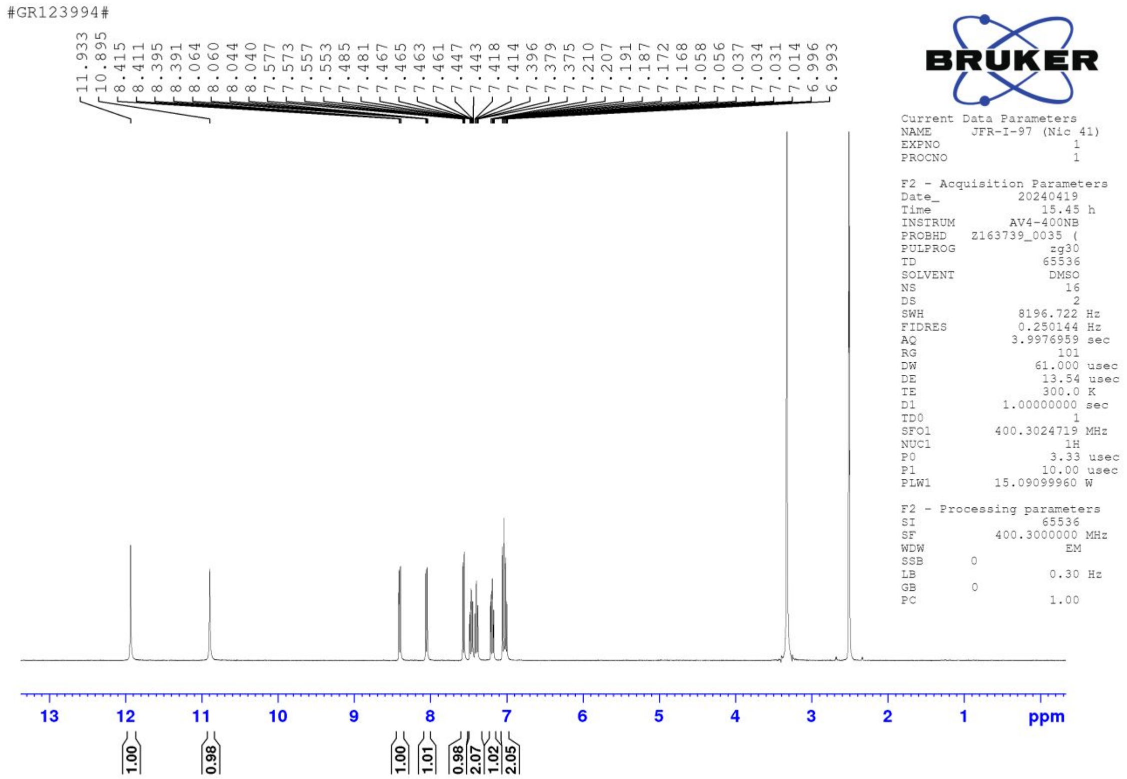

Vial:2:21
Date:25-Jun-2024
Method:C:\MassLynx\OpenLynx_Methods\ESI+_100-1250+PDA.olp
MS Method:OA_ESI+_Default

ID:mohammed210_116-03-20240625-1643
Time:17:00:45
Inlet Method:OA_Default

File:mohammed210_116-03-20240625-1643_COD
Description:Nic 41
Instrument:ACQ-QDA#KBD6021
Detectors:Waters Acquity PDA

Printed: Tue Jun 25 18:38:24 2024

**Sample Report (continued):**

**Sample 3 Nic 41 25-Jun-2024 17:00:45 File: mohammed210_116-03-20240625-1643_COD**

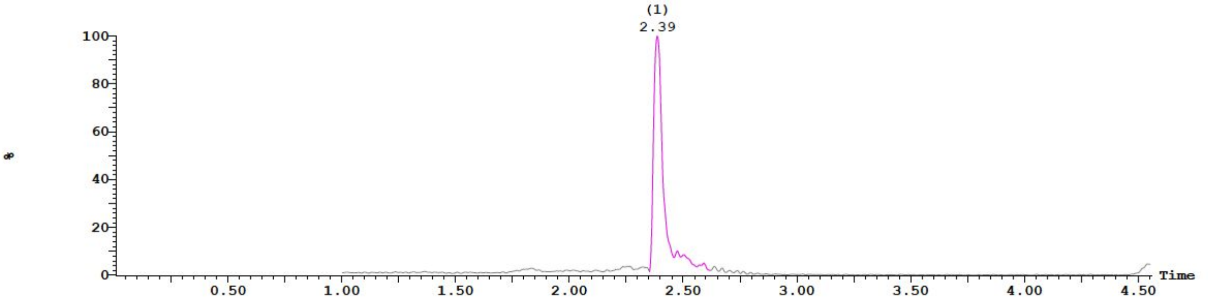


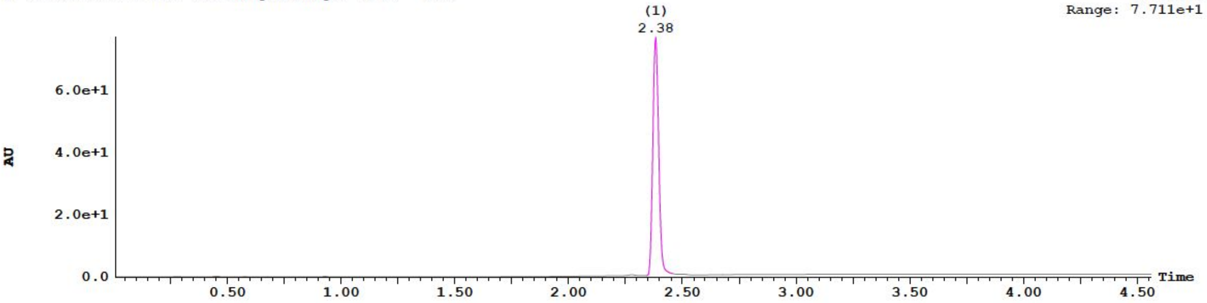


**Sample Report (continued):**

| Peak ID | Time |
|---|---|
| 1 | 2.12 |

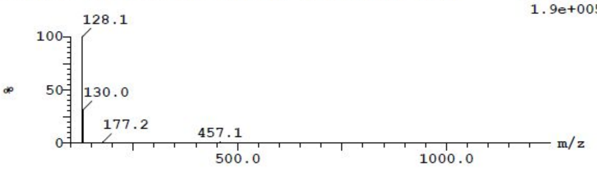


| Peak ID | Time |
|---|---|
| 2 | 2.25 |

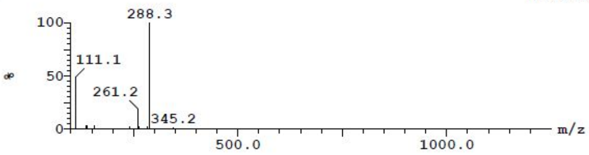


| Peak ID | Time |
|---|---|
| 3 | 2.38 |

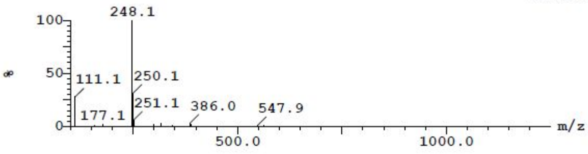


| Peak ID | Time |
|---|---|
| 4 | 2.46 |

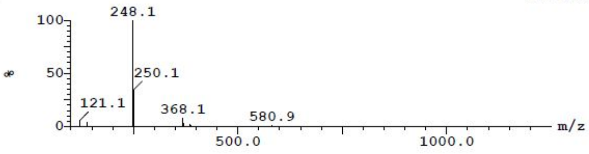


| Peak ID | Time |
|---|---|
| 5 | 2.48 |

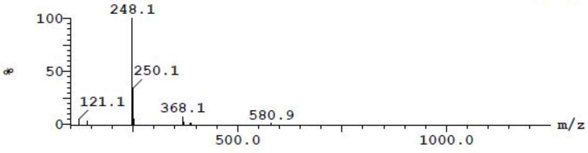


| Peak ID | Time |
|---|---|
| 5 | 2.48 |

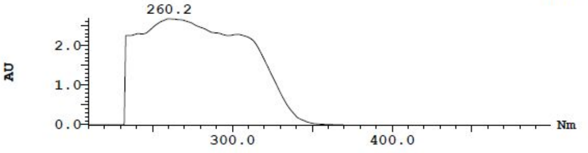

**N-(3-chlorophenyl)-2-hydroxybenzamide** (**Nic-42**)[36]

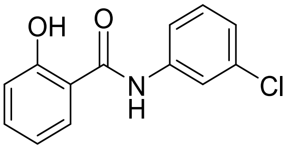


$^1$HNMR (400 MHz, DMSO-$d_6$) δ 11.54 (s, 1H), 10.47 (s, 1H), 7.94 - 7.89 (m, 2H), 7.62 (dd, *J* = 8.2 Hz, 1.0 Hz, 1H), 7.46 - 7.38 (m, 2H), 7.19 (dd, *J* = 7.9 Hz, 1.2 Hz, 1H), 7.00 - 6.95 (m, 2H); LC-MS (ESI); $[M+H]^+$: 248.1

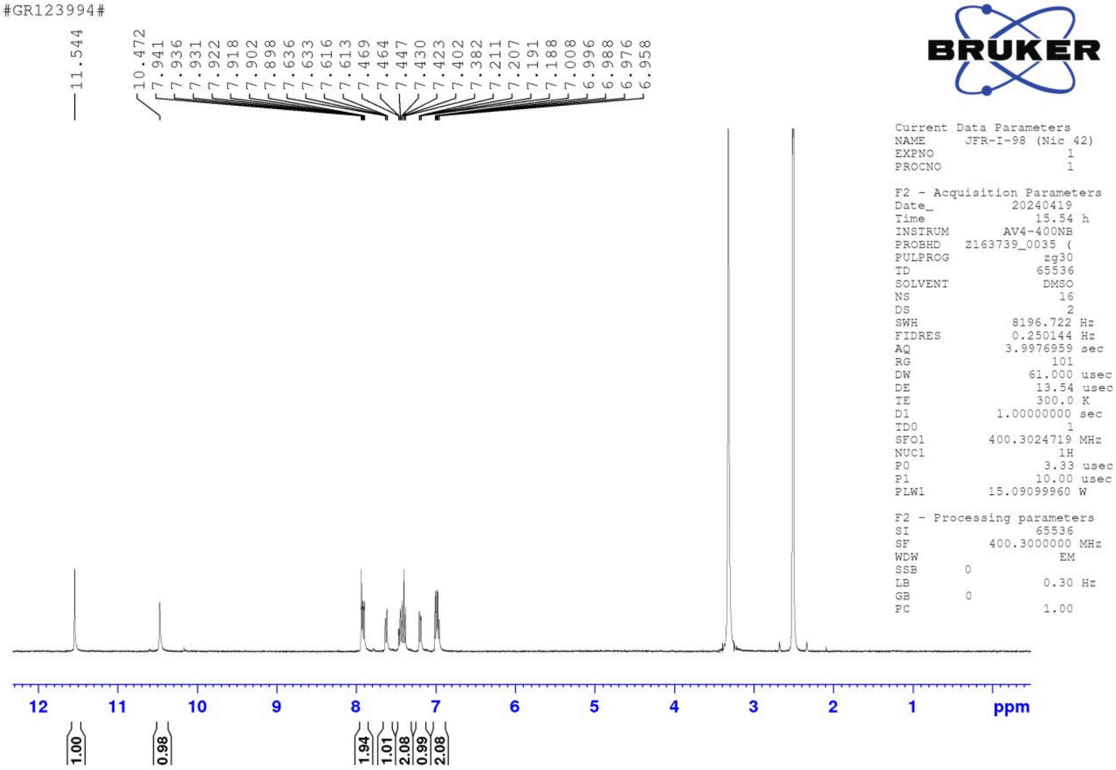

Vial:2:22
Date:25-Jun-2024
Method:C:\MassLynx\OpenLynx_Methods\ESI+_100-1250+PDA.olp
MS Method:OA_ESI+_Default

ID:mohammed210_116-04-20240625-1643
Time:17:06:41
Inlet Method:OA_Default

File:mohammed210_116-04-20240625-1643_COD
Description:Nic 42
Instrument:ACQ-QDA#KBD6021
Detectors:Waters Acquity PDA

Printed: Tue Jun 25 18:38:24 2024

**Sample Report (continued):**

**Sample 4 Nic 42 25-Jun-2024 17:06:41 File: mohammed210_116-04-20240625-1643_COD**

1: MS ES+ :TIC Smooth (SG, 2x3) 1.3e+007

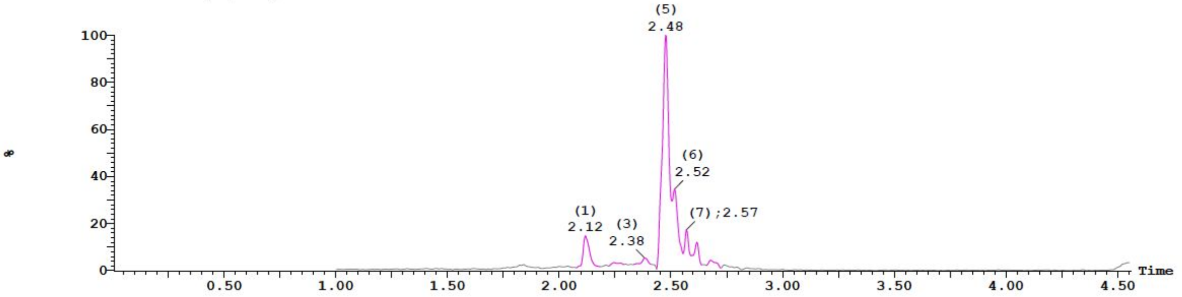


2: UV Detector: TAC: Wavelength Range: (210 - 499) 2.247e+2
Range: 2.247e+2

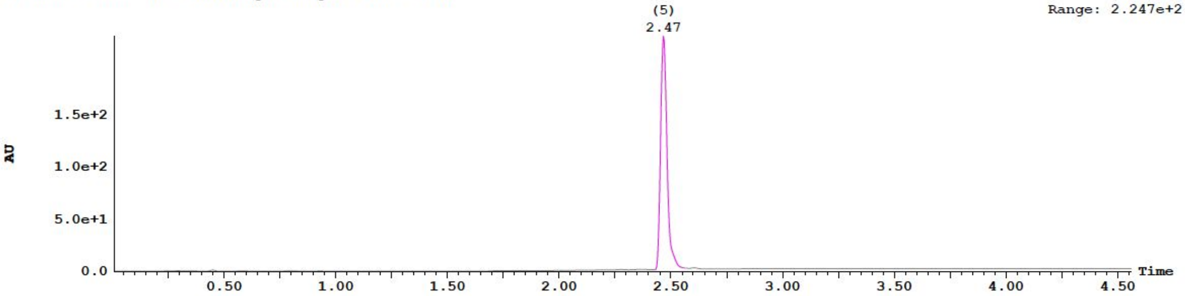


**Sample Report (continued):**

Peak ID 1 Time 2.12
1:(Time: 2.12) Combine (305:365-(275:304+366:395)) 1:MS ES+ 1.9e+005

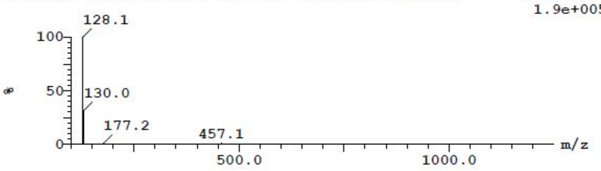


Peak ID 2 Time 2.25
2:(Time: 2.25) Combine (344:404-(314:343+405:434)) 1:MS ES+ 3.3e+004

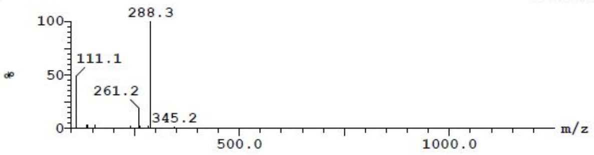


Peak ID 3 Time 2.38
3:(Time: 2.38) Combine (385:445-(355:384+446:475)) 1:MS ES+ 3.0e+005

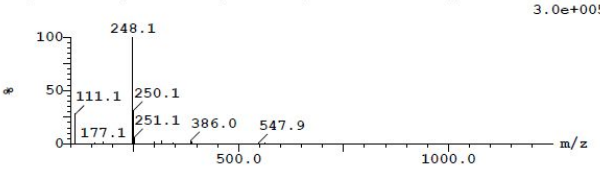


Peak ID 4 Time 2.46
4:(Time: 2.46) Combine (407:467-(377:406+468:497)) 1:MS ES+ 1.3e+006

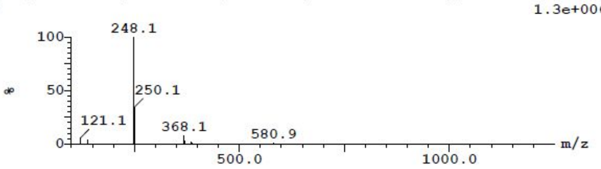


Peak ID 5 Time 2.48
5:(Time: 2.47) Combine (410:470-(380:409+471:500)) 1:MS ES+ 1.4e+006

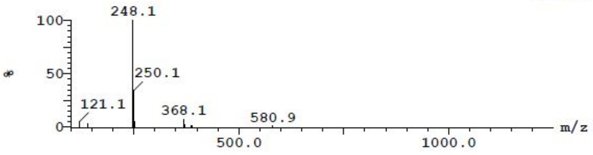


Peak ID 5 Time 2.48
5:(Time: 2.47) Combine (738) 2:UV Detector 2.674 AU

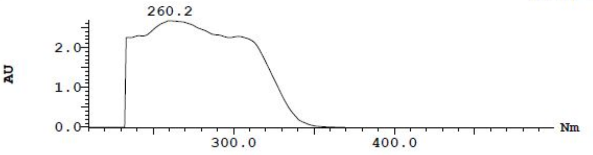

**N-(2-chloro-4-(trifluoromethyl)phenyl)-2-hydroxybenzamide** (**Nic-43**)[23]

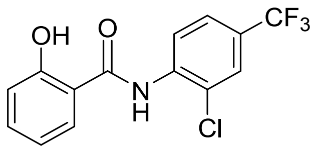

$^1$HNMR (400 MHz, DMSO-d$_6$) δ 12.08 (s, 1H), 11.26 (s, 1H), 8.76 (d, *J* = 8.2 Hz, 1.0 Hz, 1H), 8.05 (d, *J* = 7.9 Hz, 1.7 Hz, 1H), 7.98 (d, *J* = 1.6 Hz, 1H), 7.78 (dd, *J* = 9.3 Hz, 1.5 Hz, 1H), 7.50 - 7.46 (m, 1H), 7.08 - 7.00 (m, 2H); LC-MS (ESI); [M+H]$^+$: 316.1

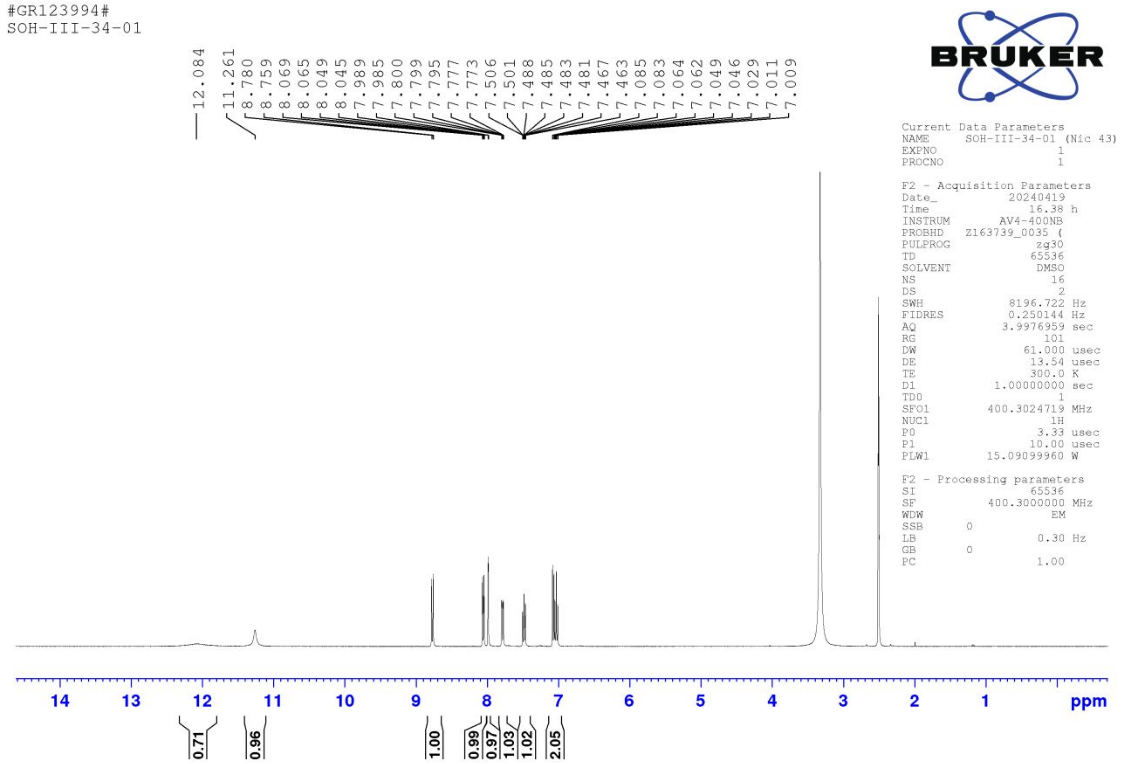

Vial:2:23 | ID:mohammed210_116-05-20240625-1643 | File:mohammed210_116-05-20240625-1643_COD
Date:25-Jun-2024 | Time:17:12:37 | Description:Nlc 43
Method:C:\MassLynx\OpenLynx_Methods\ESI+_100-1250+PDA.olp | Instrument:ACQ-QDA#KBD6021
MS Method:OA_ESI+_Default | Inlet Method:OA_Default | Detectors:Waters Acquity PDA

Printed: Tue Jun 25 18:38:24 2024

**Sample Report (continued):**

**Sample 5 Nlc 43 25-Jun-2024 17:12:37 File: mohammed210_116-05-20240625-1643_COD**

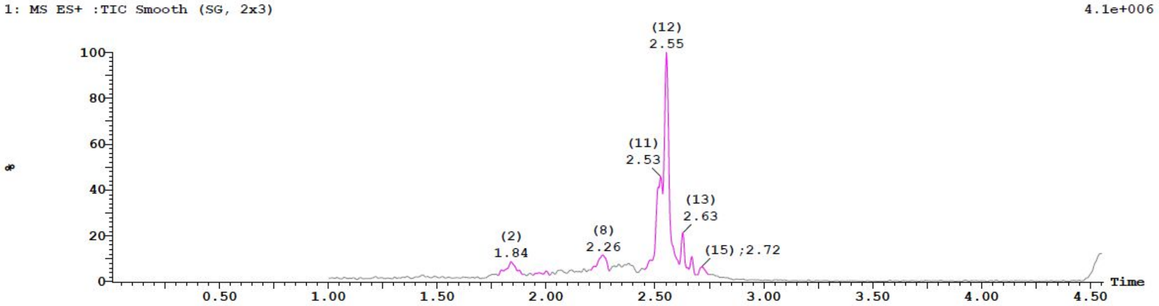


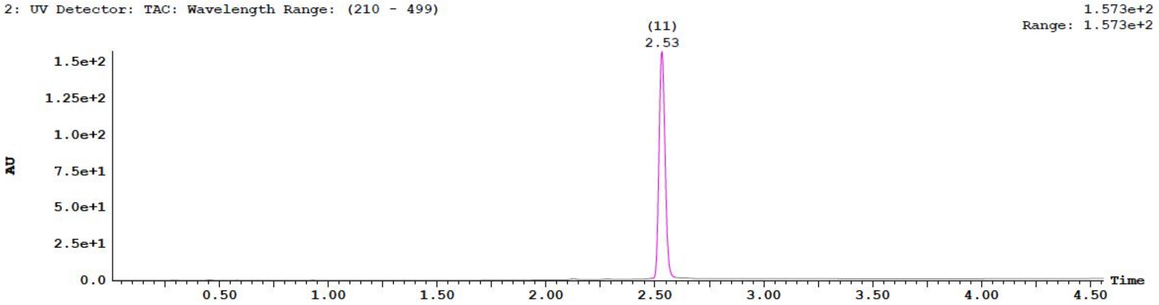


**Sample Report (continued):**

**Peak ID** 7 **Time** 2.25

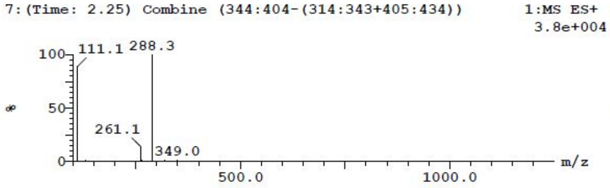


**Peak ID** 8 **Time** 2.26

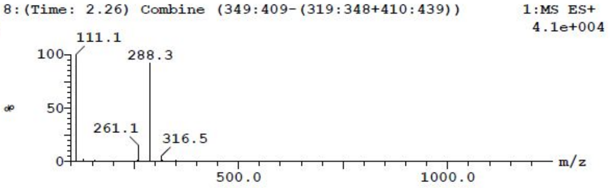


**Peak ID** 9 **Time** 2.48

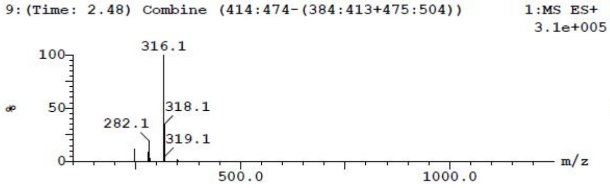


**Peak ID** 10 **Time** 2.52

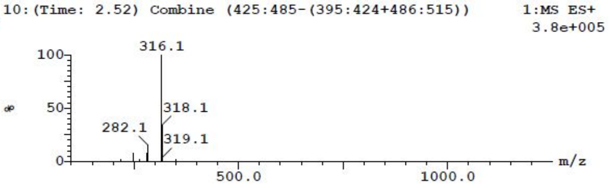


**Peak ID** 11 **Time** 2.53

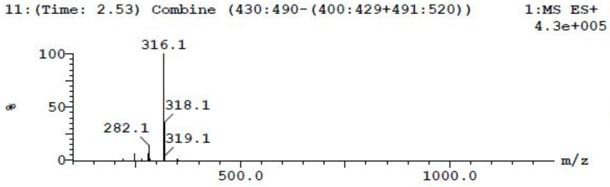


**Peak ID** 11 **Time** 2.53

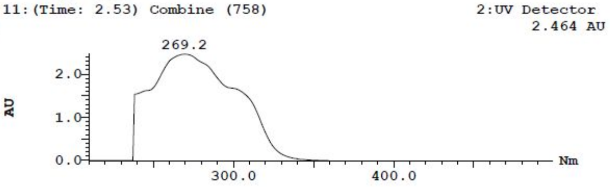

**2-hydroxy-N-(4-(trifluoromethyl)phenyl)benzamide (Nic-44)**[36]

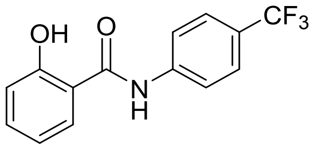


$^{1}$HNMR (400 MHz, DMSO-d$_6$) δ 11.5 (s, 1H), 10.63 (s, 1H), 7.96 - 7.90 (m, 3H), 7.73 (d, *J* = 8.5 Hz, 2H), 7.47 - 7.43 (m, 1H), 7.02 - 6.96 (m, 2H); LC-MS (ESI); [M+H]$^{+}$: 282.1

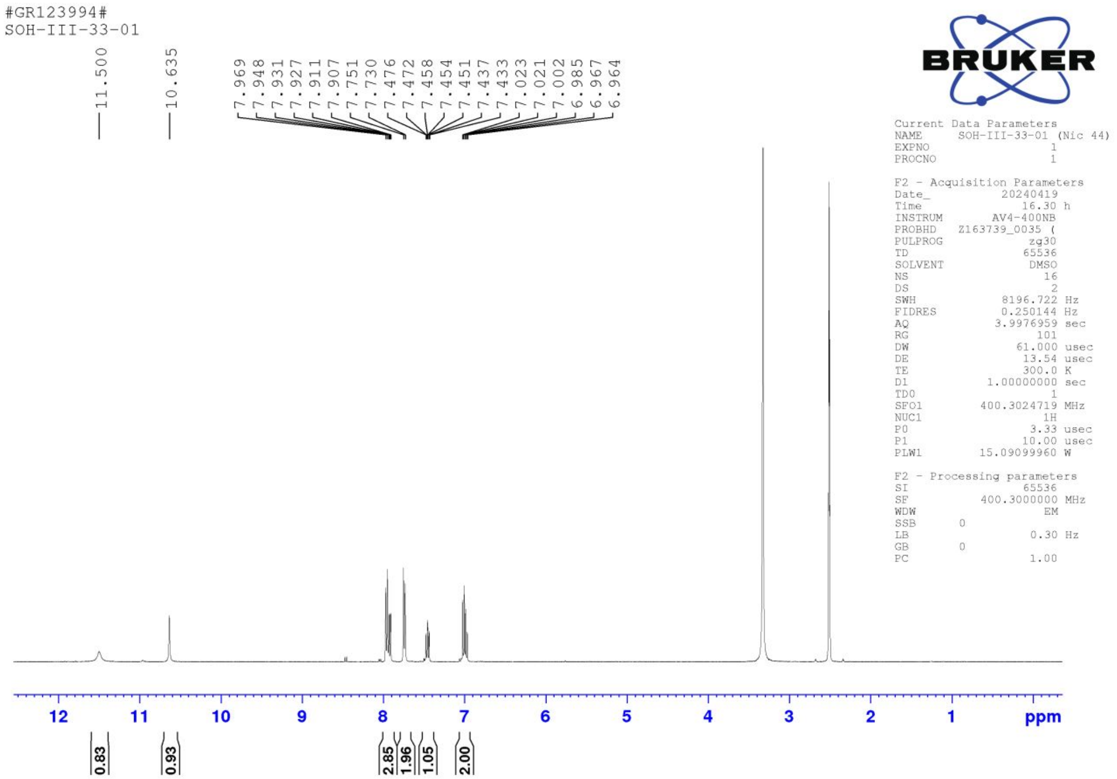

Vial:2:24 ID:mohammed210_116-06-20240625-1643 File:mohammed210_116-06-20240625-1643_COD
Date:25-Jun-2024 Time:17:18:33 Description:Nic 44
Method:C:\MassLynx\OpenLynx_Methods\ESI+_100-1250+PDA.olp Instrument:ACQ-QDA#KBD6021
MS Method:OA_ESI+_Default Inlet Method:OA_Default Detectors:Waters Acquity PDA

Printed: Tue Jun 25 18:38:24 2024

**Sample Report (continued):**

**Sample 6 Nic 44 25-Jun-2024 17:18:33 File: mohammed210_116-06-20240625-1643_COD**

1: MS ES+ :TIC Smooth (SG, 2x3) 1.2e+007

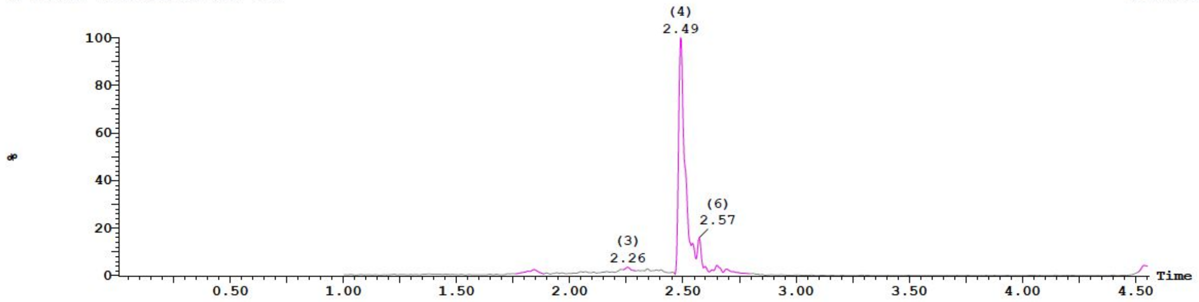


2: UV Detector: TAC: Wavelength Range: (210 - 499) 1.071e+2 Range: 1.071e+2

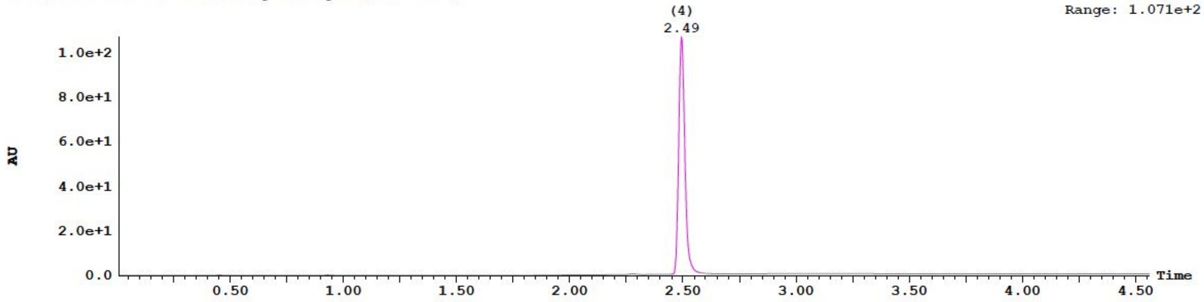


**Sample Report (continued):**

**Peak ID** 1 **Time** 1.82
1:(Time: 1.82) Combine (215:275-(185:214+276:305)) 1:MS ES+ 4.9e+004

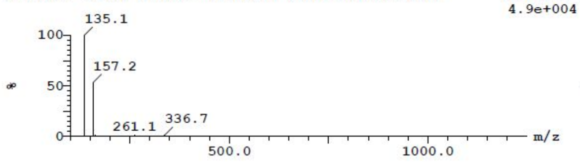


**Peak ID** 2 **Time** 1.84
2:(Time: 1.84) Combine (223:283-(193:222+284:313)) 1:MS ES+ 4.7e+004

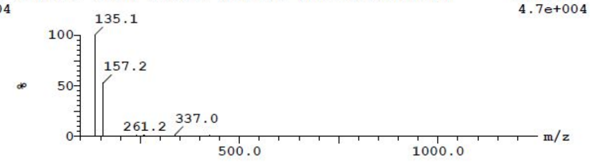


**Peak ID** 3 **Time** 2.26
3:(Time: 2.26) Combine (347:407-(317:346+408:437)) 1:MS ES+ 4.4e+004

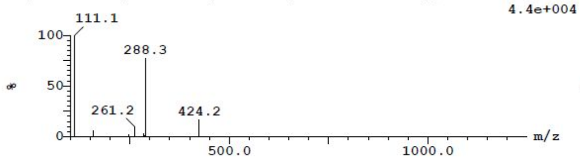


**Peak ID** 4 **Time** 2.49
4:(Time: 2.49) Combine (418:478-(388:417+479:508)) 1:MS ES+ 1.7e+006

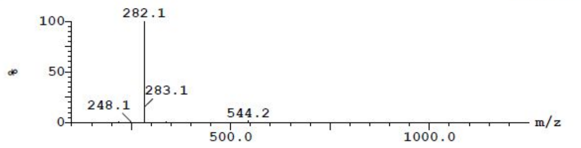


**Peak ID** 4 **Time** 2.49
4:(Time: 2.49) Combine (746) 2:UV Detector 1.761 AU

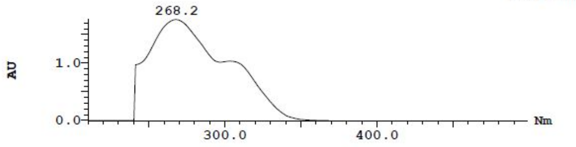


**Peak ID** 5 **Time** 2.54
5:(Time: 2.54) Combine (433:493-(403:432+494:523)) 1:MS ES+ 1.8e+006

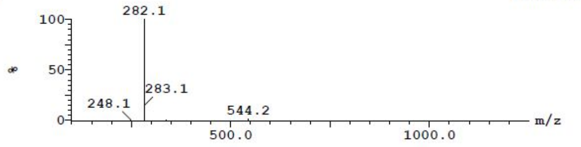

**N-(3,5-bis(trifluoromethyl)phenyl)-2-hydroxybenzamide** (**Nic-45**)[37]

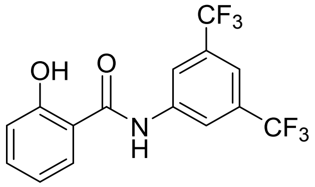

$^{1}$HNMR (400 MHz, $CDCl_3$) δ 11.4 (s, 1H), 8.17 - 8.13 (m, 3H), 7.69 (s, 1H), 7.56 - 7.48 (m, 2H), 7.06 (dd, $J$ = 8.4 Hz, 0.9 Hz, 1H), 6.98- 6.94 (m, 1H); LC-MS (ESI); $[M+H]^+$: 350.1

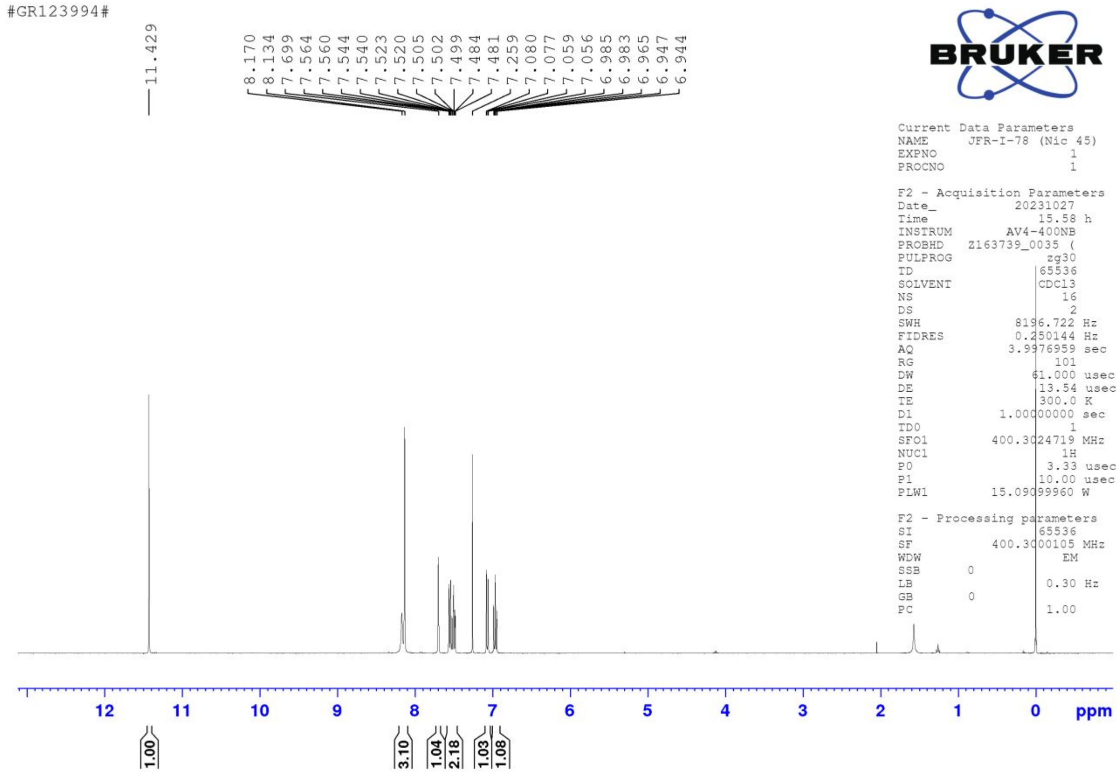

Vial:2:25
Date:25-Jun-2024
Method:C:\MassLynx\OpenLynx_Methods\ESI+_100-1250+PDA.olp
MS Method:OA_ESI+_Default

ID:mohammed210_116-07-20240625-1643
Time:17:24:30
Inlet Method:OA_Default

File:mohammed210_116-07-20240625-1643_COD
Description:Nic 45
Instrument:ACQ-QDA#KBD6021
Detectors:Waters Acquity PDA

Printed: Tue Jun 25 18:38:24 2024

**Sample Report (continued):**

**Sample 7 Nic 45 25-Jun-2024 17:24:30 File: mohammed210_116-07-20240625-1643_COD**

1: MS ES+ :TIC Smooth (SG, 2x3) 2.2e+006

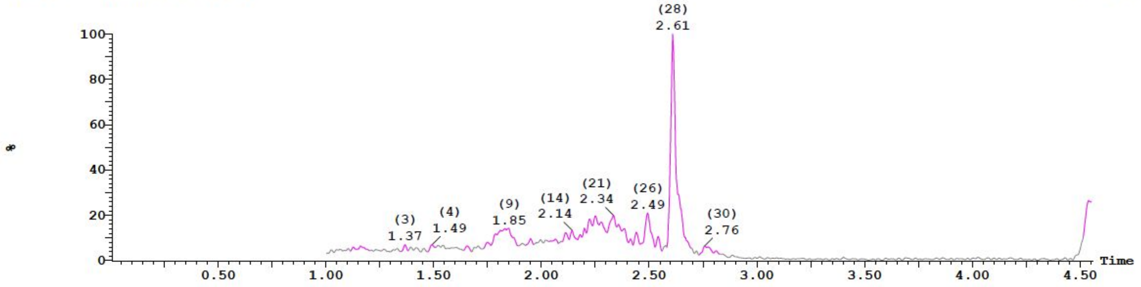

2: UV Detector: TAC: Wavelength Range: (210 - 499) 1.079e+2 Range: 1.079e+2

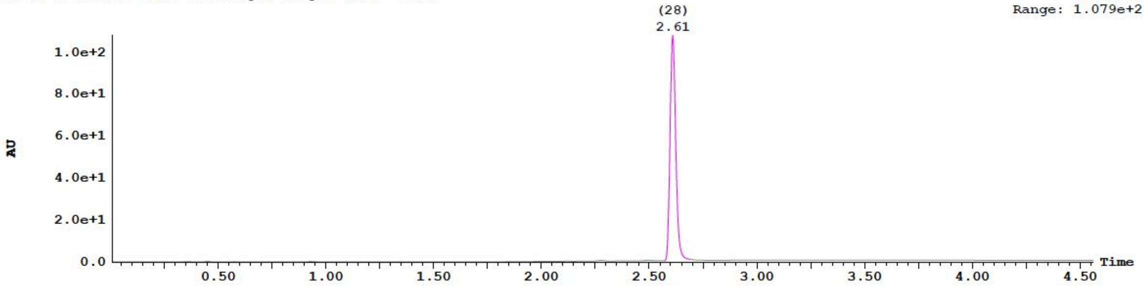

**Sample Report (continued):**

**Peak ID** 25 **Time** 2.44

25:(Time: 2.44) Combine (402:462-(372:401+463:492)) 1:MS ES+ 3.1e+004

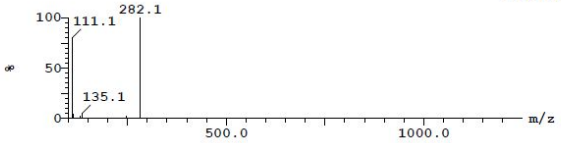

**Peak ID** 26 **Time** 2.49

26:(Time: 2.49) Combine (418:478-(388:417+479:508)) 1:MS ES+ 3.3e+004

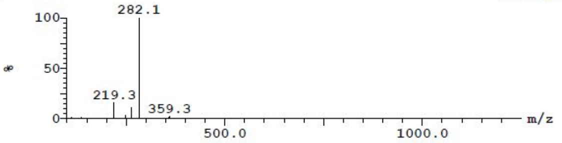

**Peak ID** 27 **Time** 2.54

27:(Time: 2.54) Combine (433:493-(403:432+494:523)) 1:MS ES+ 1.9e+005

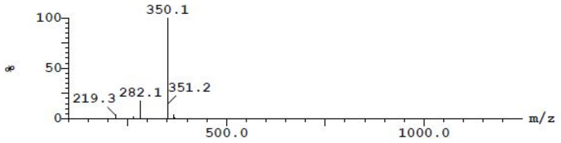

**Peak ID** 28 **Time** 2.61

28:(Time: 2.61) Combine (453:513-(423:452+514:543)) 1:MS ES+ 2.6e+005

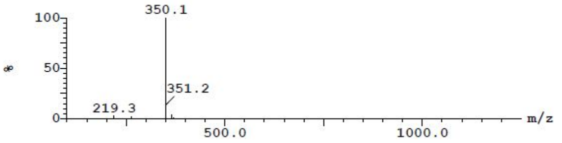

**Peak ID** 28 **Time** 2.61

28:(Time: 2.61) Combine (781) 2:UV Detector 1.884 AU

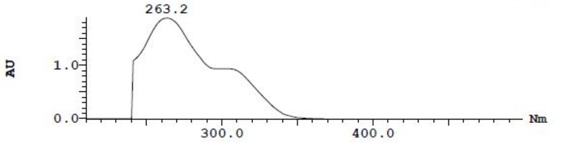

**Peak ID** 29 **Time** 2.64

29:(Time: 2.64) Combine (461:521-(431:460+522:551)) 1:MS ES+ 2.7e+005

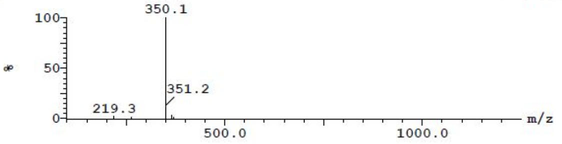

**2-hydroxy-N-(3-(trifluoromethyl)phenyl)benzamide** (**Nic-50**)[38]

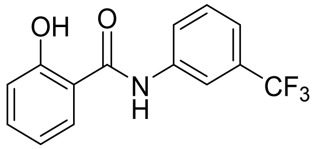


$^1$HNMR (400 MHz, DMSO-$d_6$) δ 11.51 (s, 1H), 10.60 (s, 1H), 8.22 (s, 1H), 7.96 - 7.91 (m, 2H), 7.62 (t, *J* = 8.0 Hz, 1H), 7.50 - 7.43 (m, 2H), 7.01- 6.96 (m, 2H); LC-MS (ESI); [M+H]$^+$: 282.1

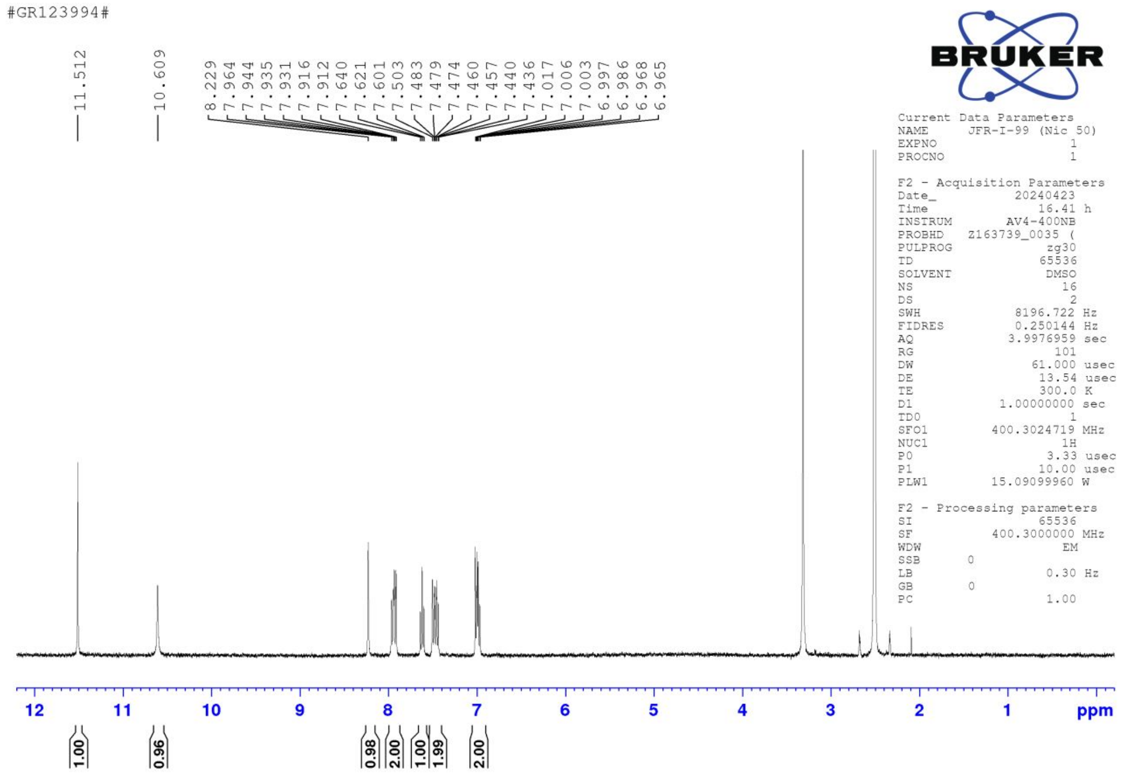

Vial:2:26
Date:25-Jun-2024
Method:C:\MassLynx\OpenLynx_Methods\ESI+_100-1250+PDA.olp
MS Method:OA_ESI+_Default

ID:mohammed210_116-08-20240625-1643
Time:17:30:23
Inlet Method:OA_Default

File:mohammed210_116-08-20240625-1643_COD
Description:Nic 50
Instrument:ACQ-QDA#KBD6021
Detectors:Waters Acquity PDA

Printed: Tue Jun 25 18:38:24 2024

**Sample Report (continued):**

**Sample 8 Nic 50 25-Jun-2024 17:30:23 File: mohammed210_116-08-20240625-1643_COD**

1: MS ES+ :TIC Smooth (SG, 2x3) 1.1e+007

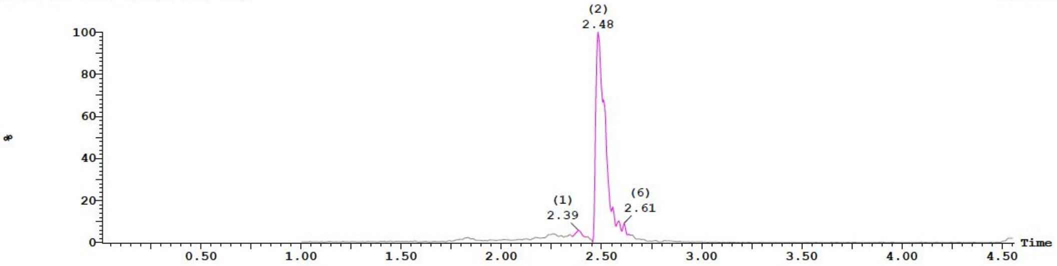


2: UV Detector: TAC: Wavelength Range: (210 - 499) 2.084e+2
Range: 2.084e+2

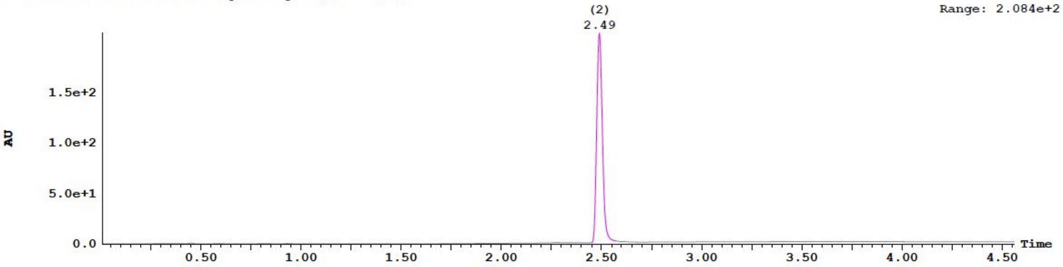




Vial:2:26
Date:25-Jun-2024
Method:C:\MassLynx\OpenLynx_Methods\ESI+_100-1250+PDA.olp
MS Method:OA_ESI+_Default

ID:mohammed210_116-08-20240625-1643
Time:17:30:23
Inlet Method:OA_Default

File:mohammed210_116-08-20240625-1643_COD
Description:Nic 50
Instrument:ACQ-QDA#KBD6021
Detectors:Waters Acquity PDA

Printed: Tue Jun 25 18:38:24 2024

**Sample Report (continued):**

| Peak ID | Time |
|---|---|
| 1 | 2.39 |

1:(Time: 2.39) Combine (386:446-(356:385+447:476)) 1:MS ES+ 7.6e+004

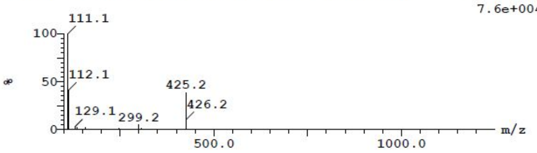


| Peak ID | Time |
|---|---|
| 2 | 2.48 |

2:(Time: 2.49) Combine (417:477-(387:416+478:507)) 1:MS ES+ 2.3e+006

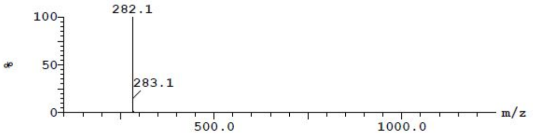


| Peak ID | Time |
|---|---|
| 2 | 2.48 |

2:(Time: 2.49) Combine (745) 2:UV Detector 2.602 AU

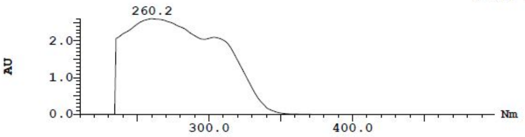


| Peak ID | Time |
|---|---|
| 3 | 2.51 |

3:(Time: 2.51) Combine (423:483-(393:422+484:513)) 1:MS ES+ 2.4e+006

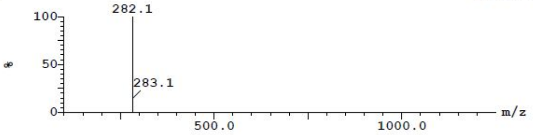


| Peak ID | Time |
|---|---|
| 4 | 2.56 |

4:(Time: 2.56) Combine (437:497-(407:436+498:527)) 1:MS ES+ 2.5e+006

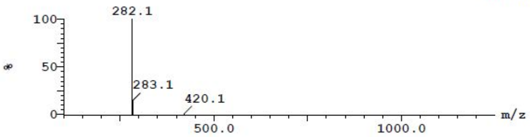


| Peak ID | Time |
|---|---|
| 5 | 2.59 |

5:(Time: 2.59) Combine (446:506-(416:445+507:536)) 1:MS ES+ 1.3e+006

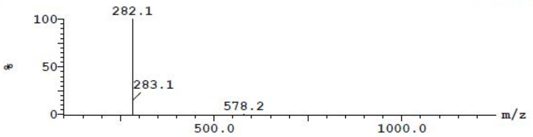

**5-chloro-2-hydroxy-N-(2-(trifluoromethyl)phenyl)benzamide** (**Nic-51**)[28]

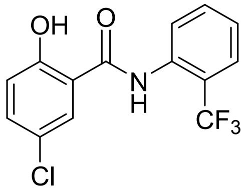


$^{1}$HNMR (400 MHz, DMSO-$d_6$) δ 11.57 (s, 1H), 10.65 (s, 1H), 7.94 (d, $J$ = 8.4 Hz, 2H), 7.88 (d, $J$ = 2.6 Hz, 1H), 7.74 (d, $J$ = 8.6 Hz, 2H), 7.47 (dd, $J$ = 8.8 Hz, 2.7 Hz, 1H), 7.03 (d, $J$ = 8.7 Hz, 1H); LC-MS (ESI); $[M+H]^+$: 282.1

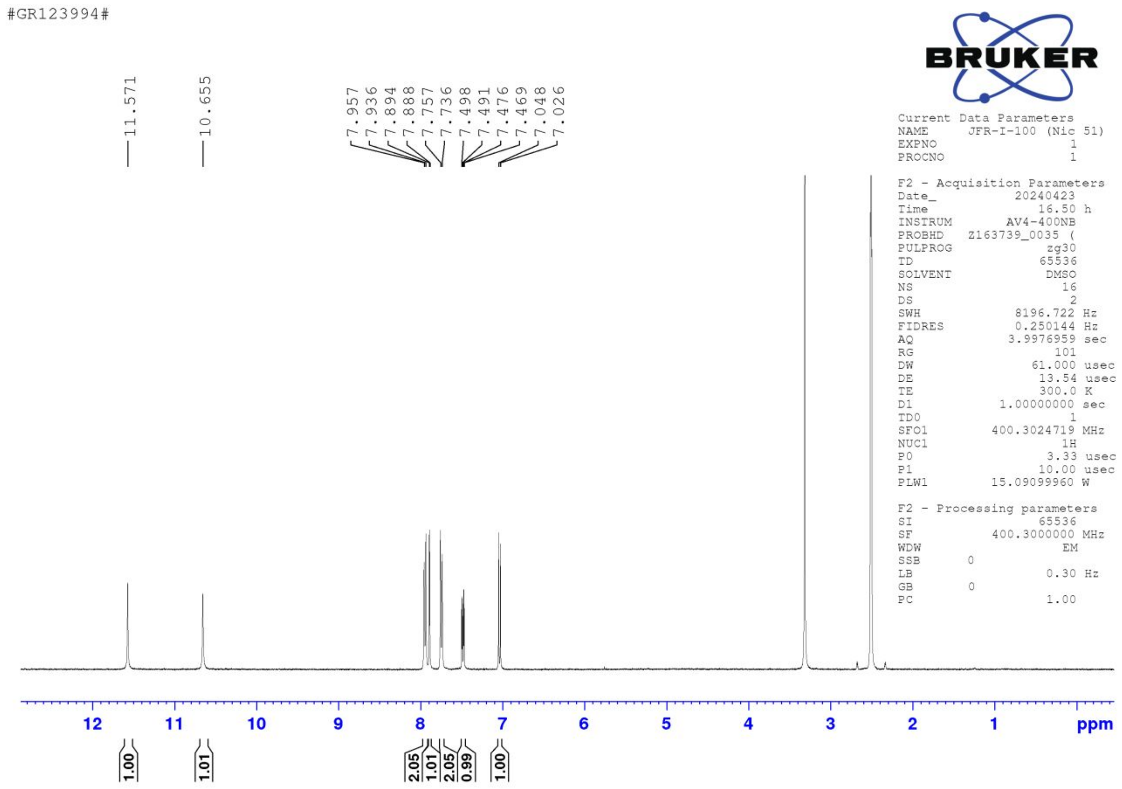

**Sample Report (continued):**

**Sample 9 Nic 51 25-Jun-2024 17:36:18 File: mohammed210_116-09-20240625-1643_COD**

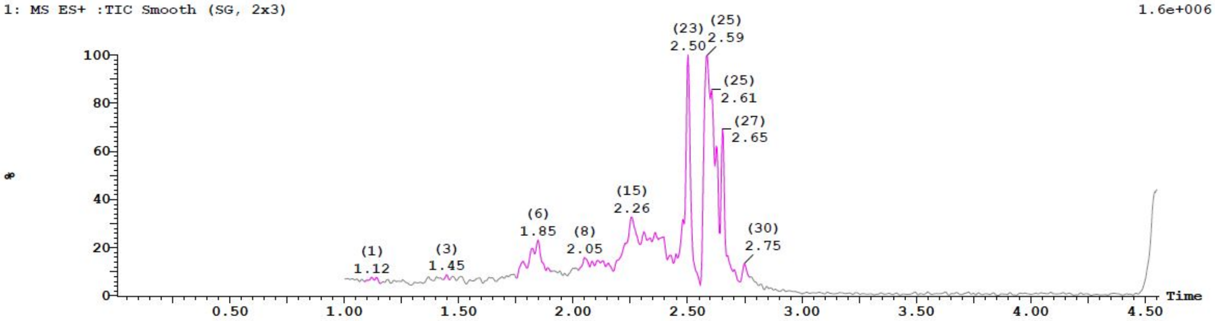


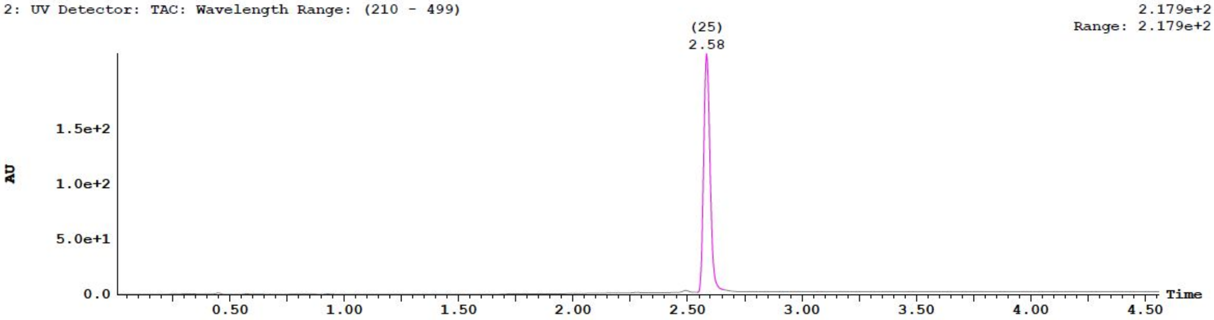


**Sample Report (continued):**

**Peak ID** 19 **Time** 2.40

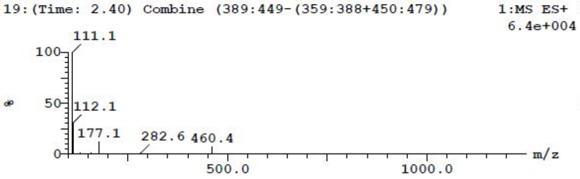


**Peak ID** 20 **Time** 2.43

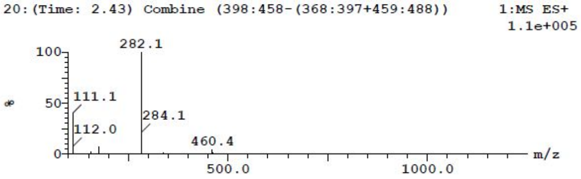


**Peak ID** 21 **Time** 2.45

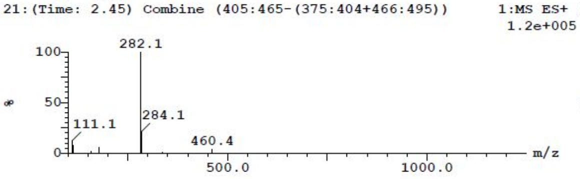


**Peak ID** 22 **Time** 2.48

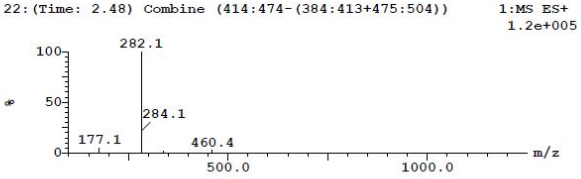


**Peak ID** 23 **Time** 2.50

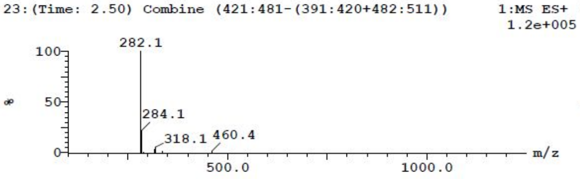


**Peak ID** 24 **Time** 2.58

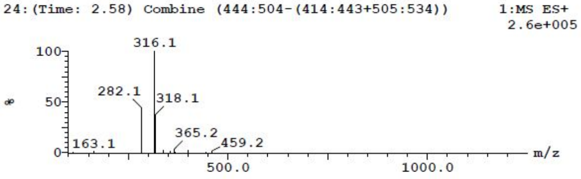

**5-bromo-N-(2-chloro-4-nitrophenyl)-2-hydroxybenzamide (Nic-52)[39]**

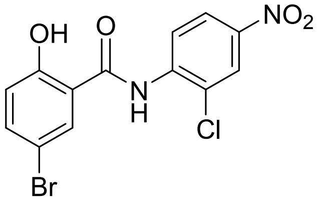


(Grey solid, 62% yield): $^1$HNMR (400 MHz, DMSO-$d_6$) δ 12.52 (bs, 1H), 11.30 (s, 1H), 8.80 (d, *J* = 9.2 Hz, 1H), 8.43 (d, 2.6 Hz, 1H), 8.30 (dd, *J* = 9.2 Hz, 2.6 Hz, 1H), 8.09 (d, *J* = 2.6 Hz, 1H), 7.65 (dd, *J* = 8.7 Hz, 2.6 Hz, 1H), 7.04 (d, *J* = 8.7 Hz, 1H); LC-MS (ESI); [M-1]: 371.0

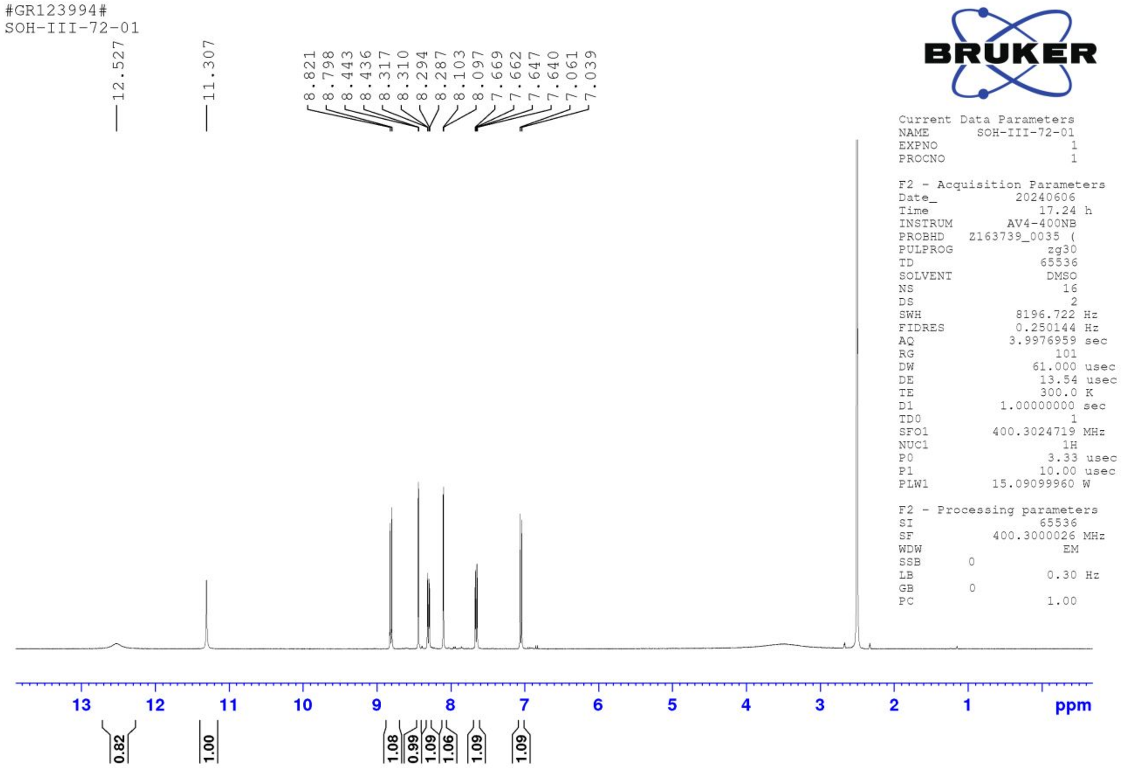

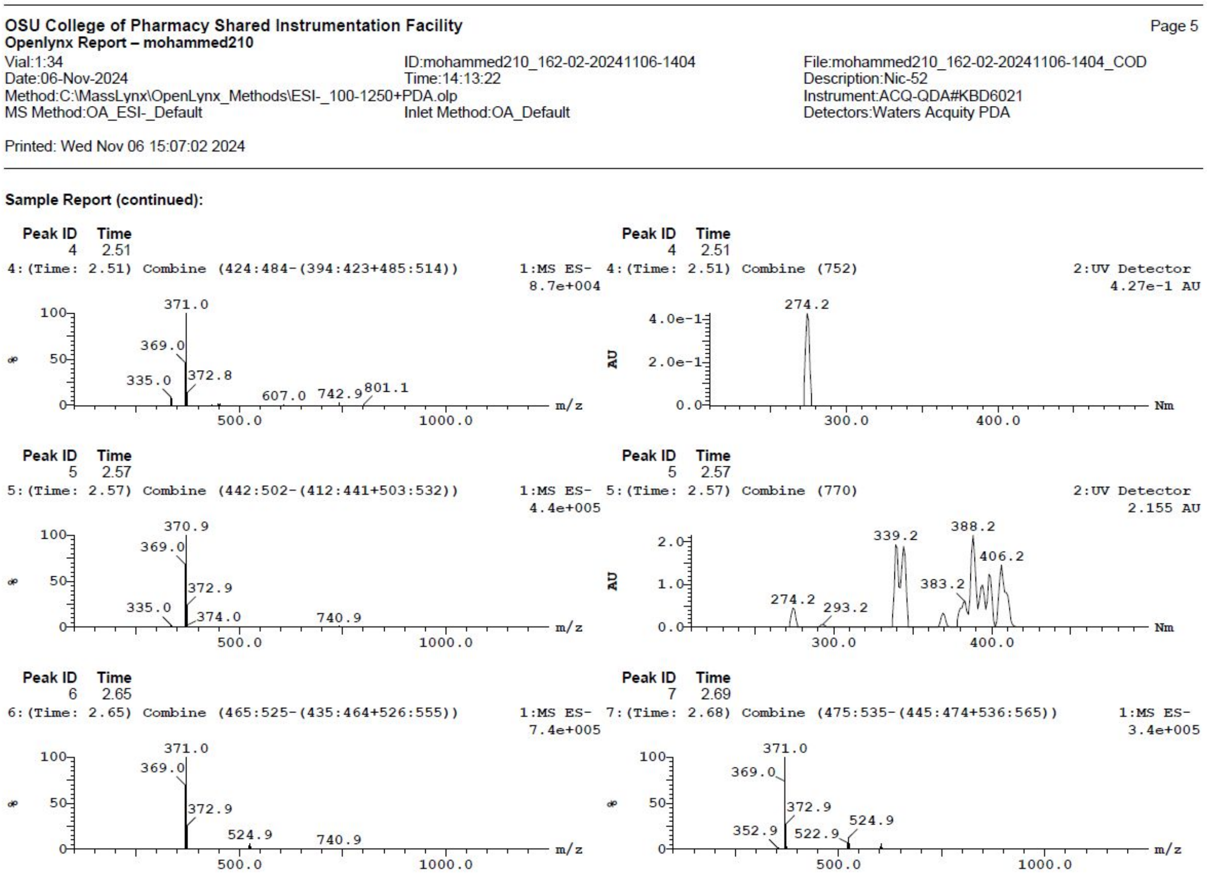


## N-(2-chloro-4-nitrophenyl)-5-fluoro-2-hydroxybenzamide (Nic-53)[40]

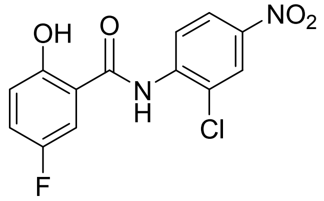


(Grey solid, 51% yield): $^1$HNMR (400 MHz, DMSO-$d_6$) δ 12.23 (bs, 1H), 11.41 (s, 1H), 8.82 (d, *J* = 9.2 Hz, 1H), 8.43 (d, 2.6 Hz, 1H), 8.29 (dd, *J* = 9.2 Hz, 2.6 Hz, 1H), 7.72 (d, *J* = 9.6 Hz, 3.3 Hz, 1H), 7.40 - 7.35 (m, 1H), 7.10 - 7.07 (m, 1H); LC-MS (ESI); $[M-H]^-$: 309

#GR123994#

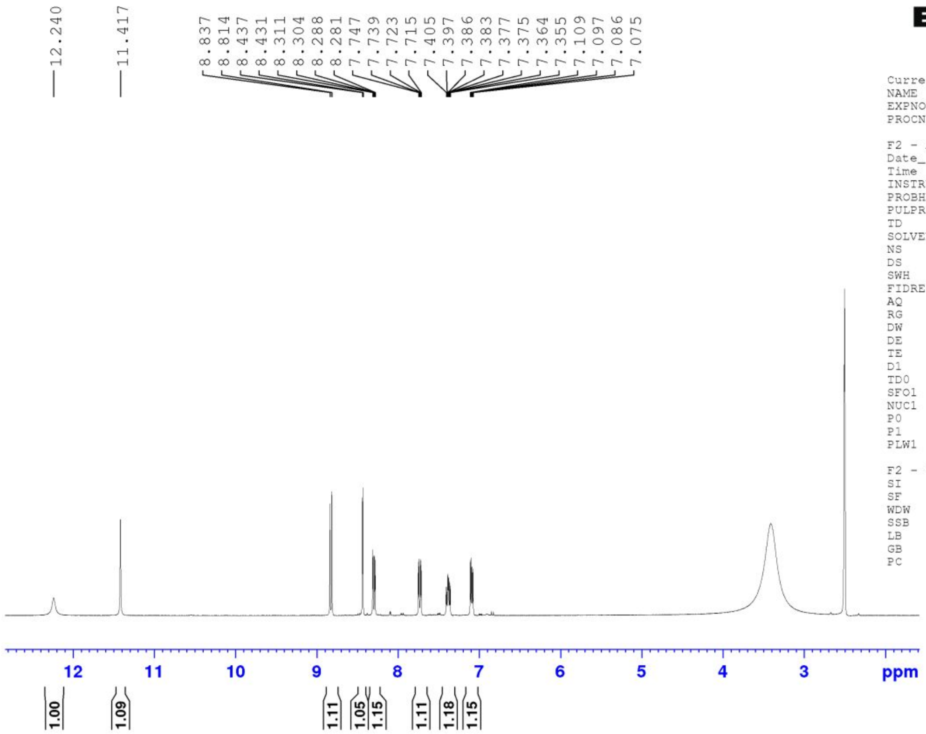


BRUKER

Current Data Parameters
NAME SOH-III-76-01
EXPNO 10
PROCNO 1

F2 - Acquisition Parameters
Date_ 20240617
Time 14.36 h
INSTRUM AV4-400NB
PROBHD Z163739_0035 (
PULPROG zg30
TD 65536
SOLVENT DMSO
NS 16
DS 2
SWH 8196.722 Hz
FIDRES 0.250144 Hz
AQ 3.9976959 sec
RG 101
DW 61.000 usec
DE 13.54 usec
TE 300.0 K
D1 1.00000000 sec
TD0 1
SFO1 400.3024719 MHz
NUC1 1H
P0 3.33 usec
P1 10.00 usec
PLW1 15.09099960 W

F2 - Processing parameters
SI 65536
SF 400.3000029 MHz
WDW EM
SSB 0
LB 0.30 Hz
GB 0
PC 1.00

**OSU College of Pharmacy Shared Instrumentation Facility** Page 11
**Openlynx Report – mohammed210**

Vial:1:35 ID:mohammed210_162-03-20241106-1404 File:mohammed210_162-03-20241106-1404_COD
Date:06-Nov-2024 Time:14:19:16 Description:Nic-53
Method:C:\MassLynx\OpenLynx_Methods\ESI-_100-1250+PDA.olp Instrument:ACQ-QDA#KBD6021
MS Method:OA_ESI-_Default Inlet Method:OA_Default Detectors:Waters Acquity PDA

Printed: Wed Nov 06 15:07:02 2024

**Sample Report (continued):**

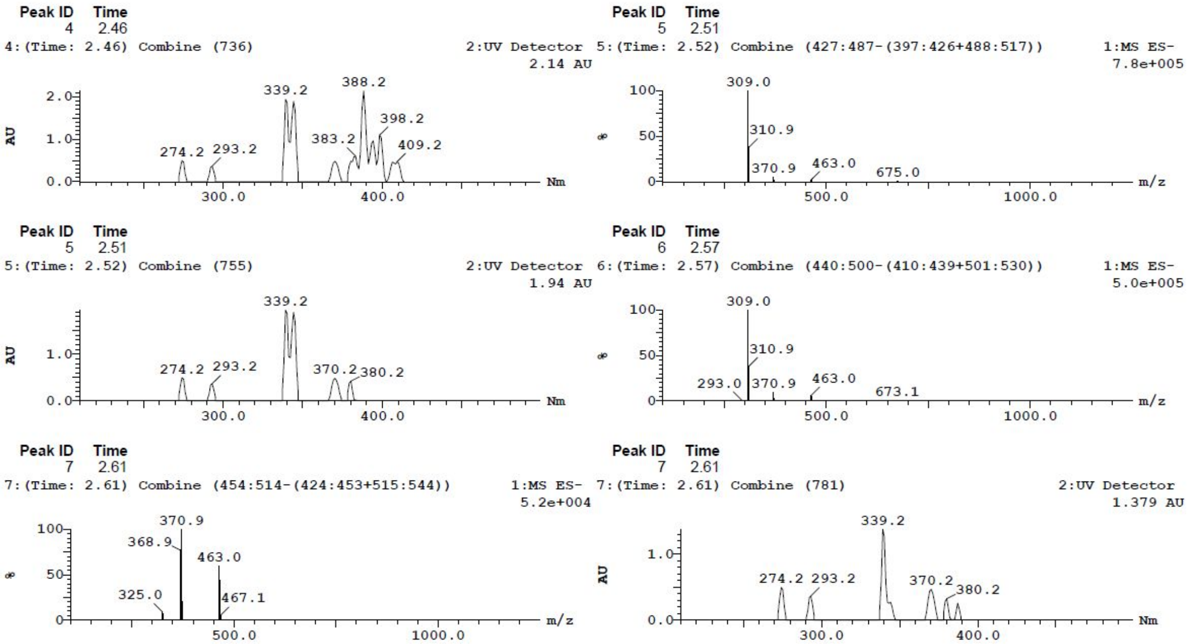


**N-(2-chloro-4-nitrophenyl)-2-hydroxy-5-methylbenzamide (Nic-54)[41]**

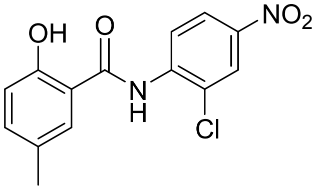


(Grey solid, 64% yield): $^1$HNMR (400 MHz, DMSO-$d_6$) δ 12.11 (bs, 1H), 11.62 (s, 1H), 9.06 (d, $J$ = 9.2 Hz, 1H), 8.63 (d, $J$ = 2.6 Hz, 1H), 8.50 (dd, $J$ = 9.2 Hz, 2.6 Hz, 1H), 8.05 (d, $J$ = 1.8 Hz, 1H), 7.51 (dd, $J$ = 8.3 Hz, 2.0 Hz, 1H), 7.19 (d, $J$ = 8.2 Hz, 1H), 2.49 (s, 3H); LC-MS (ESI); [M-H]$^-$: 305.0

#GR123994#
SOH-III-67-02

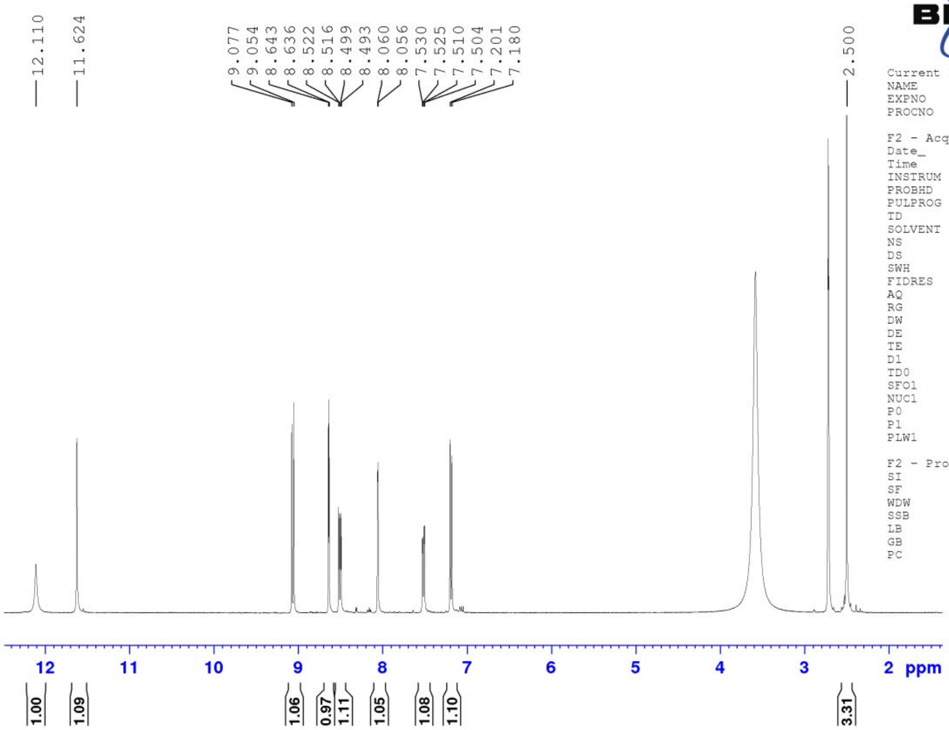


Current Data Parameters
NAME SOH-III-67-02
EXPNO 10
PROCNO 1

F2 - Acquisition Parameters
Date_ 20240604
Time 15.16 h
INSTRUM AV4-400NB
PROBHD Z163739_0035 (
PULPROG zg30
TD 65536
SOLVENT DMSO
NS 16
DS 2
SWH 8196.722 Hz
FIDRES 0.250144 Hz
AQ 3.9976959 sec
RG 101
DW 61.000 usec
DE 13.54 usec
TE 300.0 K
D1 1.00000000 sec
TD0 1
SFO1 400.3024719 MHz
NUC1 1H
P0 3.33 usec
P1 10.00 usec
PLW1 15.09099960 W

F2 - Processing parameters
SI 65536
SF 400.2999156 MHz
WDW EM
SSB 0
LB 0.30 Hz
GB 0
PC 1.00

Vial:1:36
Date:06-Nov-2024
Method:C:\MassLynx\OpenLynx_Methods\ESI-_100-1250+PDA.olp
MS Method:OA_ESI-_Default
ID:mohammed210_162-04-20241106-1404
Time:14:25:11
Inlet Method:OA_Default
File:mohammed210_162-04-20241106-1404_COD
Description:Nic-54
Instrument:ACQ-QDA#KBD6021
Detectors:Waters Acquity PDA

Printed: Wed Nov 06 15:07:02 2024

**Sample Report (continued):**

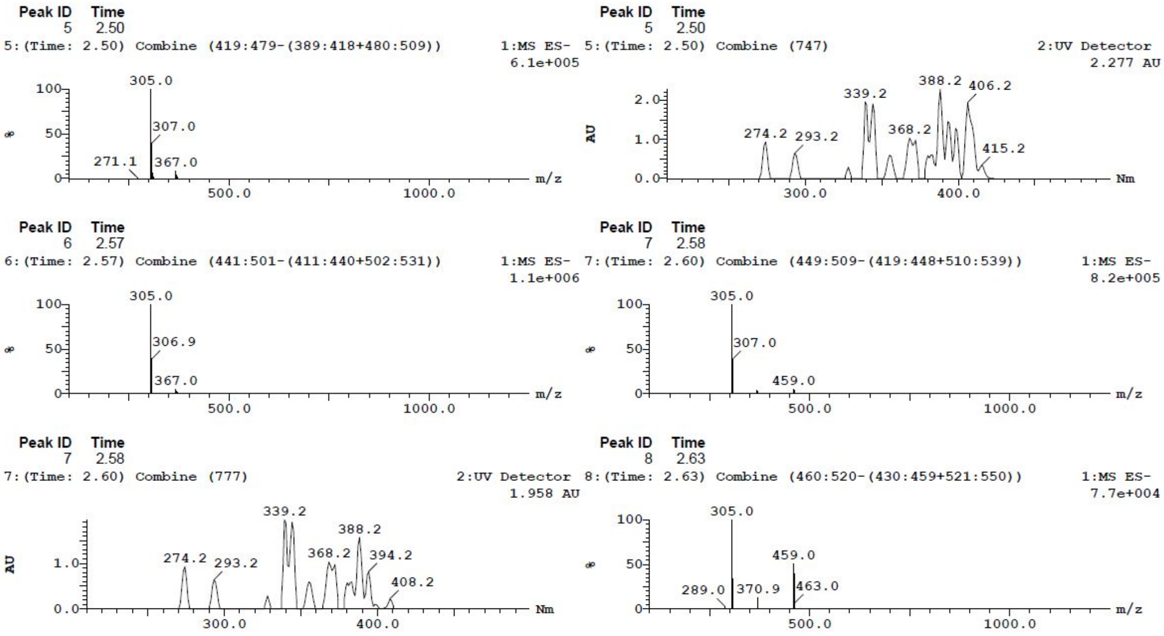


**N-(2-chloro-4-nitrophenyl)-2,5-dihydroxybenzamide (Nic-55)[39]**

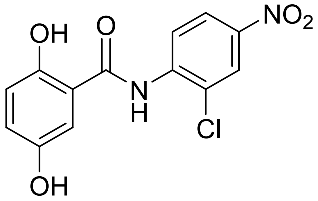


(Yellow solid, 25% yield): $^1$HNMR (400 MHz, DMSO-$d_6$) δ 11.51 - 11.44 (m, 2H), 9.22 (s, 1H), 8.85 (d, *J* = 9.2 Hz, 1H), 8.42 (d, *J* = 2.6 Hz, 1H), 8.28 (dd, *J* = 9.2 Hz, 2.6 Hz, 1H), 7.45 - 7.44 (m, 1H), 6.91 (d, *J* = 2.4 Hz, 2H); LC-MS (ESI); [M-H]$^-$: 307

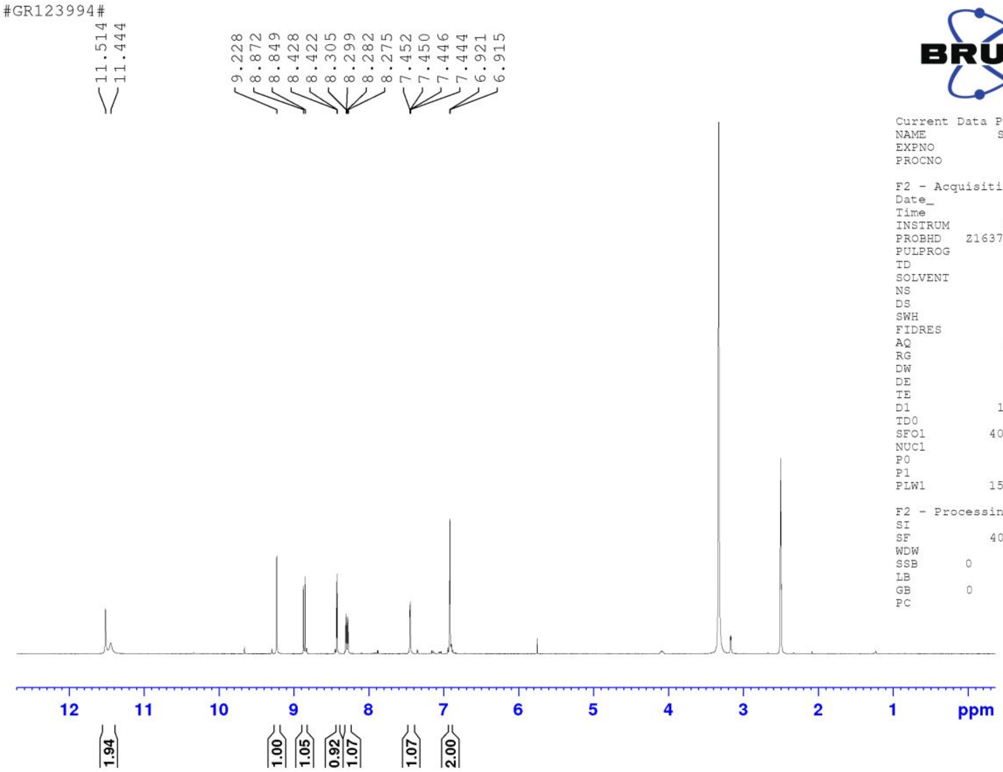


```
Current Data Parameters
NAME          SOH-III-81
EXPNO                  1
PROCNO                 1

F2 - Acquisition Parameters
Date_           20240617
Time               14.59 h
INSTRUM        AV4-400NB
PROBHD   Z163739_0035 (
PULPROG             zg30
TD                 65536
SOLVENT             DMSO
NS                    16
DS                     2
SWH             8196.722 Hz
FIDRES          0.250144 Hz
AQ             3.9976959 sec
RG                   101
DW                61.000 usec
DE                 13.54 usec
TE                 300.0 K
D1            1.00000000 sec
TD0                    1
SFO1         400.3024719 MHz
NUC1                  1H
P0                  3.33 usec
P1                 10.00 usec
PLW1         15.09099960 W

F2 - Processing parameters
SI                 65536
SF           400.3000028 MHz
WDW                   EM
SSB      0
LB                  0.30 Hz
GB       0
PC                  1.00
```

OSU College of Pharmacy Shared Instrumentation Facility
Openlynx Report – mohammed210

Page 18

Vial:1:37
Date:06-Nov-2024
Method:C:\MassLynx\OpenLynx_Methods\ESI-_100-1250+PDA.olp
MS Method:OA_ESI-_Default

ID:mohammed210_162-05-20241106-1404
Time:14:31:07
Inlet Method:OA_Default

File:mohammed210_162-05-20241106-1404_COD
Description:Nic-55
Instrument:ACQ-QDA#KBD6021
Detectors:Waters Acquity PDA

Printed: Wed Nov 06 15:07:02 2024

**Sample Report (continued):**

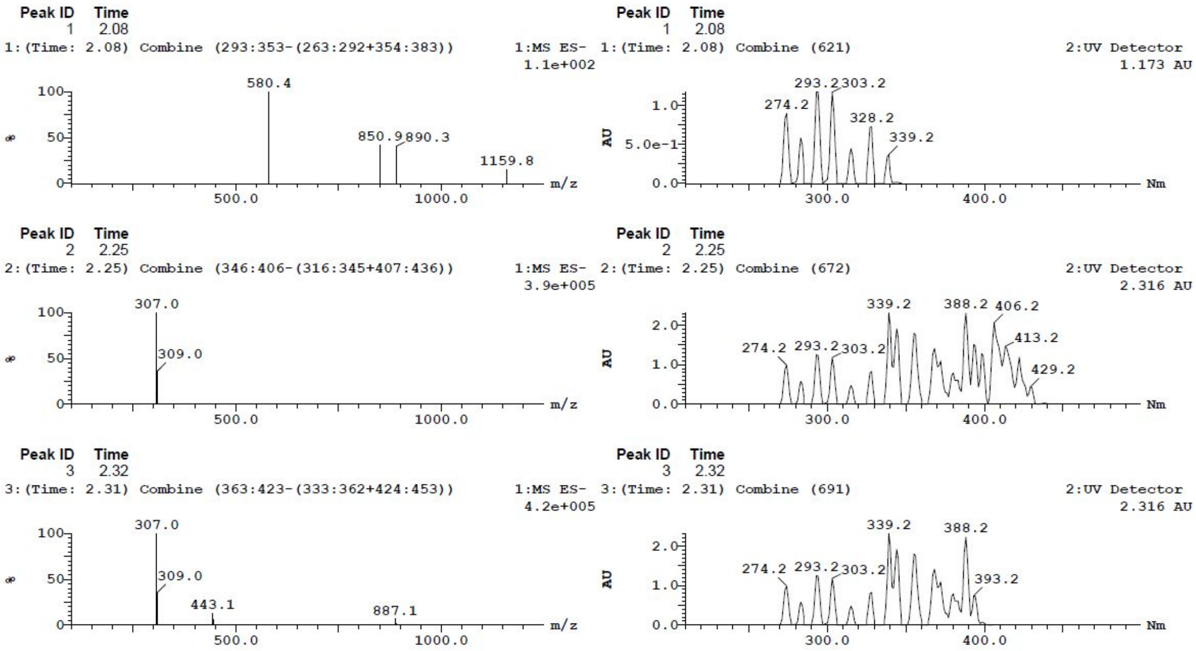


**N-(3,5-bis(trifluoromethyl)phenyl)-2,5-dihydroxybenzamide (Nic-56)[38]**

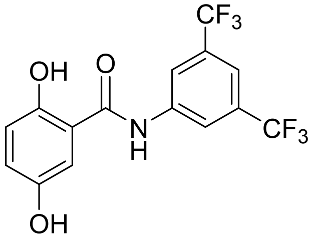


(White solid, 38% yield): $^1$HNMR (400 MHz, DMSO-$d_6$) δ 10.81 - 10.65 (m, 2H), 9.14 (s, 1H), 8.45 (s, 2H), 7.80 (s, 1H), 7.28 (d, $J$ = 2.8 Hz, 1H), 6.92 - 6.84 (m, 2H); LC-MS (ESI); [M-H]$^-$: 364

#GR123994#
SOH-III-93-01

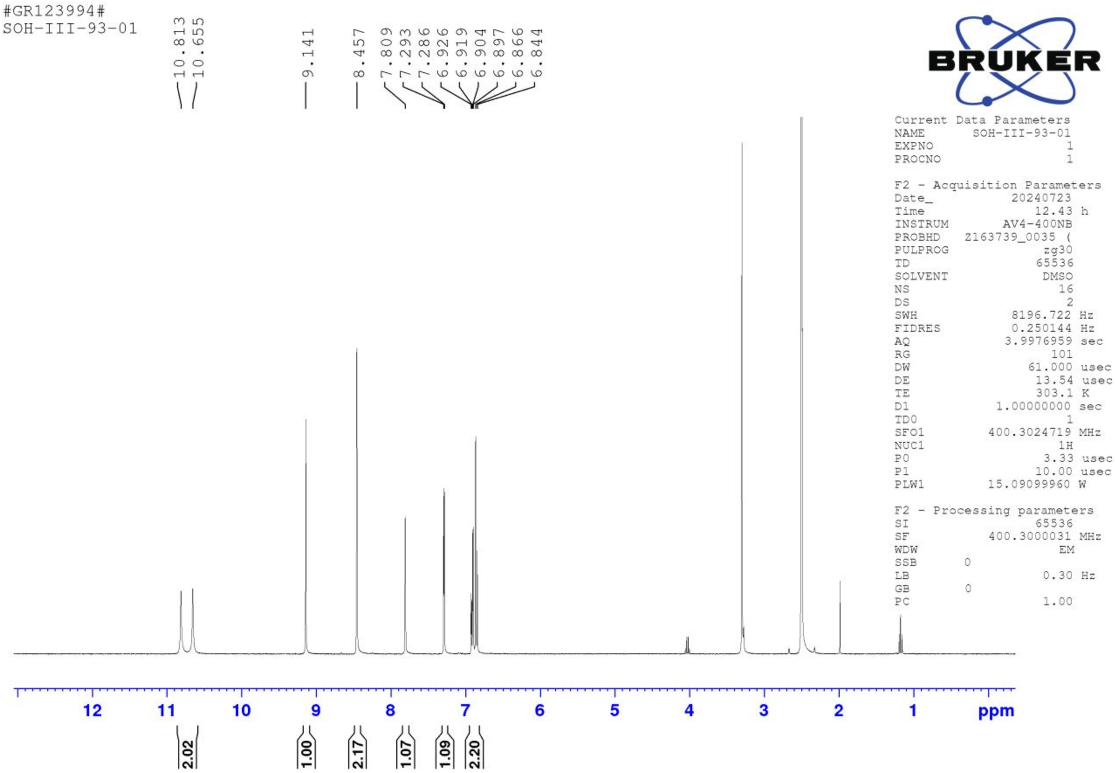


Current Data Parameters
NAME SOH-III-93-01
EXPNO 1
PROCNO 1

F2 - Acquisition Parameters
Date_ 20240723
Time 12.43 h
INSTRUM AV4-400NB
PROBHD Z163739_0035 (
PULPROG zg30
TD 65536
SOLVENT DMSO
NS 16
DS 2
SWH 8196.722 Hz
FIDRES 0.250144 Hz
AQ 3.9976959 sec
RG 101
DW 61.000 usec
DE 13.54 usec
TE 303.1 K
D1 1.00000000 sec
TD0 1
SFO1 400.3024719 MHz
NUC1 1H
P0 3.33 usec
P1 10.00 usec
PLW1 15.09099960 W

F2 - Processing parameters
SI 65536
SF 400.3000031 MHz
WDW EM
SSB 0
LB 0.30 Hz
GB 0
PC 1.00

**OSU College of Pharmacy Shared Instrumentation Facility** Page 23
**Openlynx Report – mohammed210**
Vial:1:38 ID:mohammed210_162-06-20241106-1404 File:mohammed210_162-06-20241106-1404_COD
Date:06-Nov-2024 Time:14:37:04 Description:Nic-56
Method:C:\MassLynx\OpenLynx_Methods\ESI-_100-1250+PDA.olp Instrument:ACQ-QDA#KBD6021
MS Method:OA_ESI-_Default Inlet Method:OA_Default Detectors:Waters Acquity PDA

Printed: Wed Nov 06 15:07:02 2024

**Sample Report (continued):**

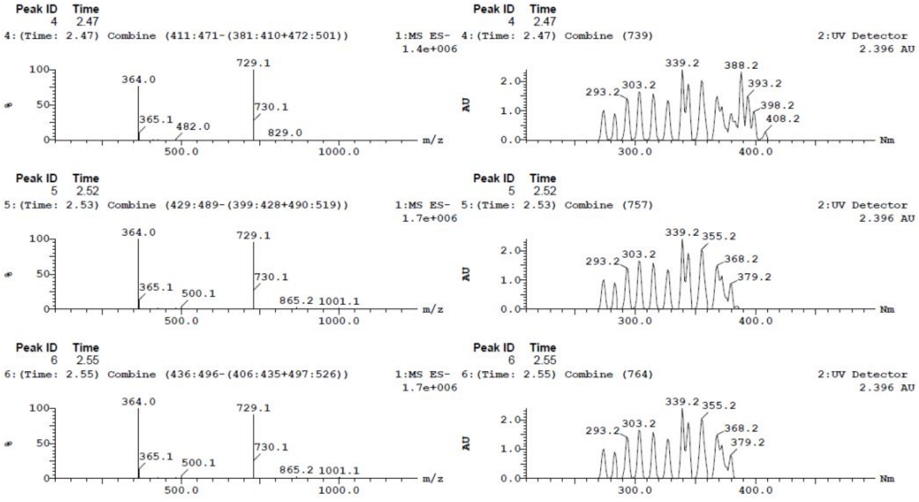

**4-chloro-N-(2-chloro-4-nitrophenyl)-2-hydroxybenzamide (Nic-57)[40]**

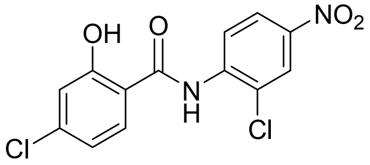

(Grey solid, 61% yield): $^{1}$HNMR (400 MHz, DMSO-d$_6$) δ 12.70 (bs, 1H), 11.22 (s, 1H), 8.81 (d, *J* = 9.2 Hz, 1H), 8.41 (d, *J* = 2.6 Hz, 1H), 8.27 (dd, *J* = 9.2 Hz, 2.6 Hz, 1H), 8.03 (d, *J* = 8.7 Hz, 1H), 7.10 - 7.08 (m, 2H); LC-MS (ESI); [M-H]$^-$: 325.0

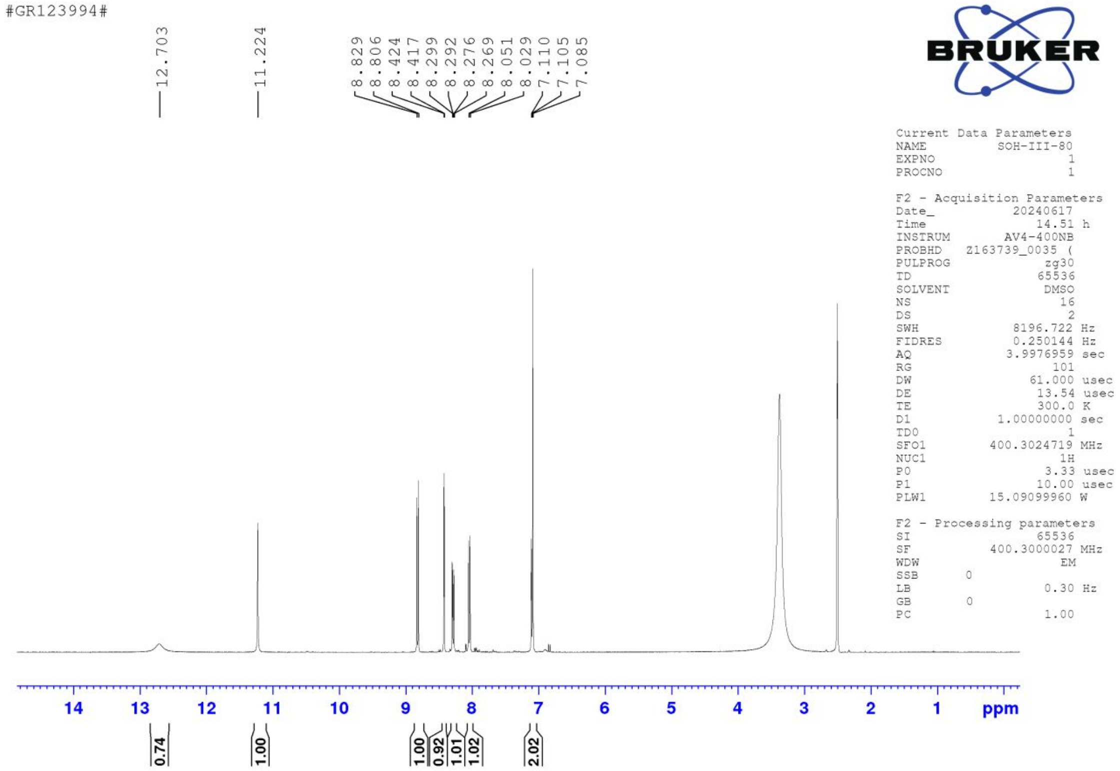

OSU College of Pharmacy Shared Instrumentation Facility
Openlynx Report – mohammed210



Vial:1:39
Date:06-Nov-2024
Method:C:\MassLynx\OpenLynx_Methods\ESI-_100-1250+PDA.olp
MS Method:OA_ESI-_Default

ID:mohammed210_162-07-20241106-1404
Time:14:43:00
Inlet Method:OA_Default

File:mohammed210_162-07-20241106-1404_COD
Description:Nic-57
Instrument:ACQ-QDA#KBD6021
Detectors:Waters Acquity PDA

Printed: Wed Nov 06 15:07:02 2024

**Sample Report (continued):**

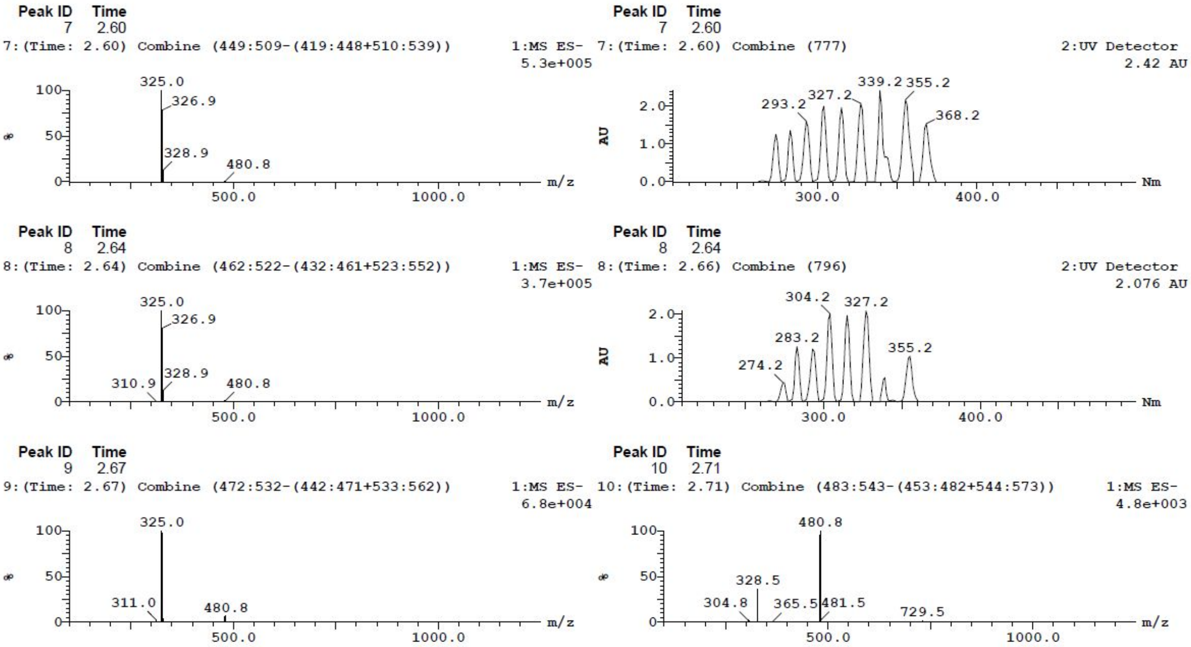


**3-chloro-N-(2-chloro-4-nitrophenyl)-2-hydroxybenzamide (Nic-58)**

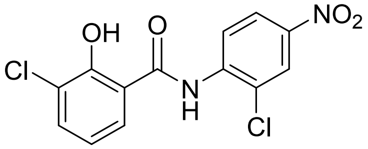


(Yellow solid, 52% yield): $^{1}$HNMR (400 MHz, DMSO-$d_6$) δ 11.4 (bs, 1H), 8.55 (d, $J$ = 9.1 Hz, 1H), 8.43 (d, $J$ = 2.6 Hz, 1H), 8.31- 8.28 (m, 1H), 8.02 (dd, $J$ = 8.0 Hz, 1.6 Hz, 1H), 7.70 (dd, $J$ = 7.8 Hz, 1.6 Hz, 1H), 7.07 (t, $J$ = 7.9 Hz, 1H), 6.89 (bs, 1H); LC-MS (ESI); [M-H]$^-$: 325.0

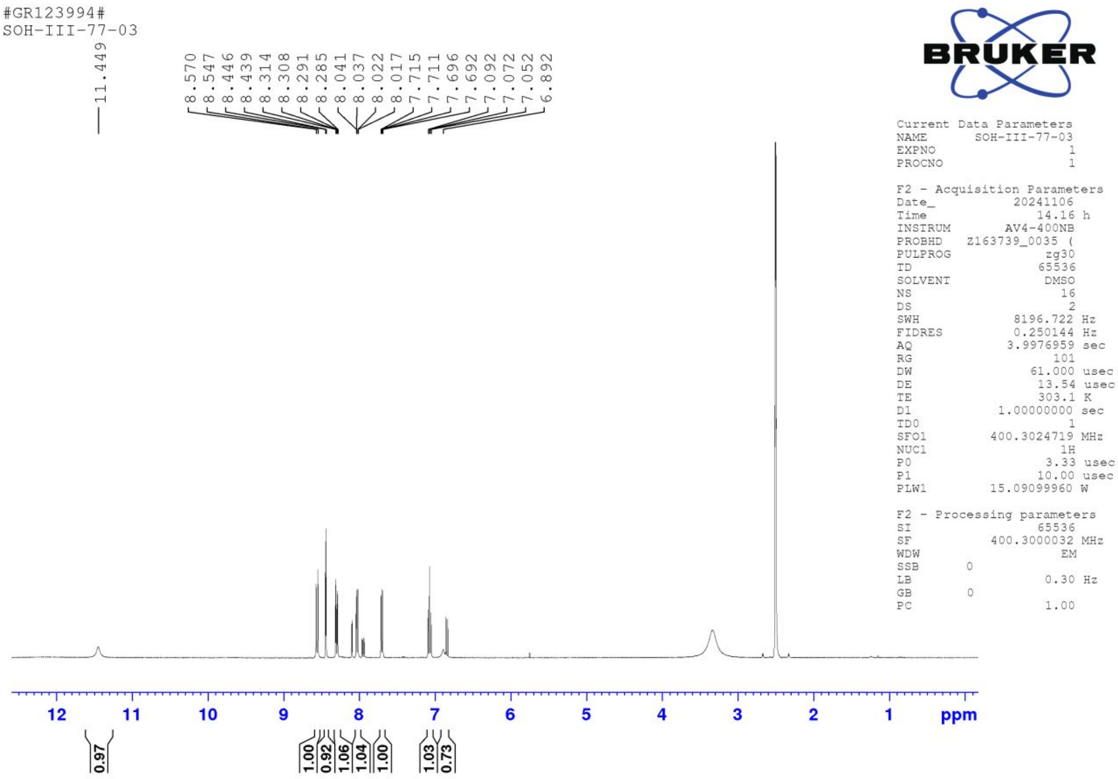




**Openlynx Report – mohammed210**

Vial:1:40
Date:06-Nov-2024
Method:C:\MassLynx\OpenLynx_Methods\ESI-_100-1250+PDA.olp
MS Method:OA_ESI-_Default

ID:mohammed210_162-08-20241106-1404
Time:14:48:56
Inlet Method:OA_Default

File:mohammed210_162-08-20241106-1404_COD
Description:Nic-58
Instrument:ACQ-QDA#KBD6021
Detectors:Waters Acquity PDA

Printed: Wed Nov 06 15:07:02 2024

**Sample Report (continued):**

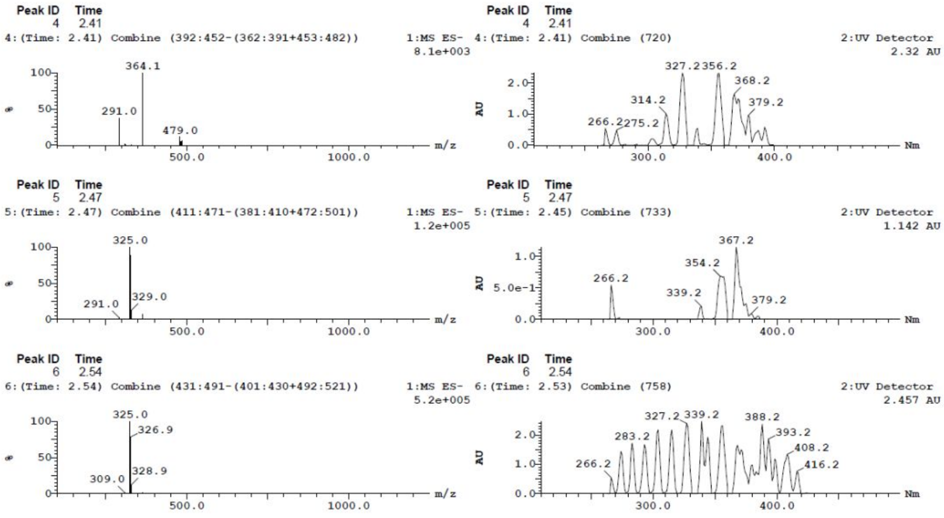

**5-bromo-2-hydroxy-N-(4-(trifluoromethyl)phenyl)benzamide (Nic-59)[28]**

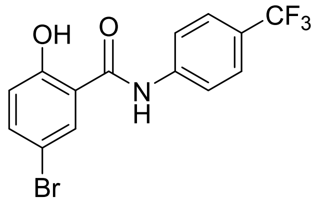

(White solid, 56% yield): $^{1}$HNMR (400 MHz, DMSO-$d_6$) δ 11.58 (bs, 1H), 10.63 (s, 1H), 7.99 (d, *J* = 2.5 Hz, 1H), 7.94 - 7.92 (m, 2H), 7.74 - 7.72 (m, 2H), 7.58 (dd, *J* = 8.7 Hz, 2.5 Hz, 1H), 6.97 (d, *J* = 8.7 Hz, 1H); LC-MS (ESI); $[M-H]^-$: 360.0

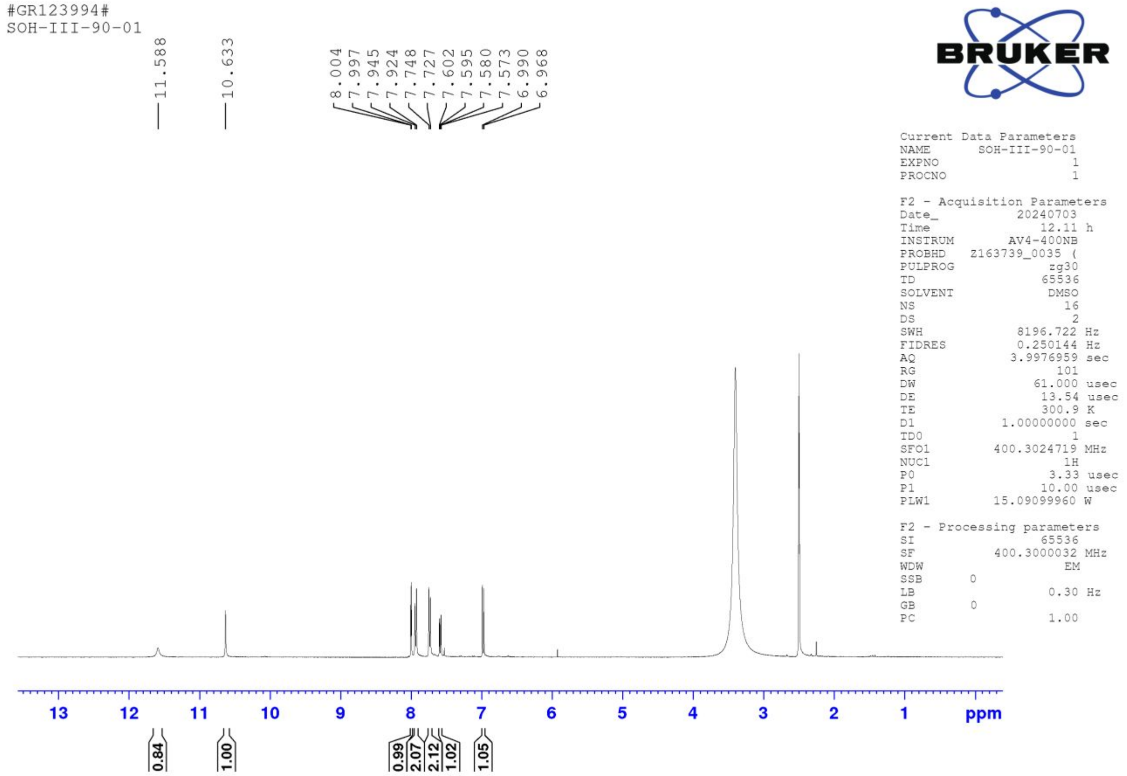

Vial:1:41
Date:06-Nov-2024
Method:C:\MassLynx\OpenLynx_Methods\ESI-_100-1250+PDA.olp
MS Method:OA_ESI-_Default
ID:mohammed210_162-09-20241106-1404
Time:14:54:55
Inlet Method:OA_Default
File:mohammed210_162-09-20241106-1404_COD
Description:Nic-59
Instrument:ACQ-QDA#KBD6021
Detectors:Waters Acquity PDA

Printed: Wed Nov 06 15:07:02 2024

**Sample Report (continued):**

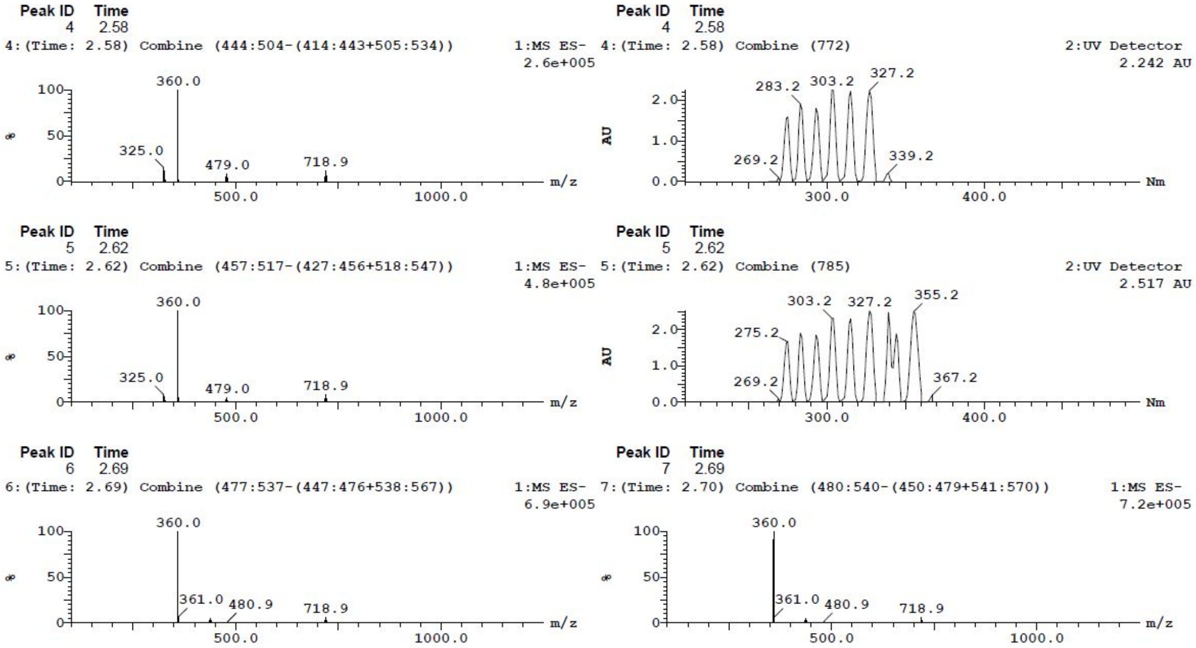


**5-fluoro-2-hydroxy-N-(4-(trifluoromethyl)phenyl)benzamide (Nic-60)[42]**

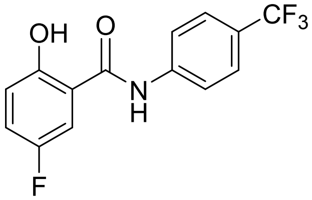


(White solid, 47% yield): $^{1}$HNMR (400 MHz, DMSO-$d_6$) δ 11.38 (bs, 1H), 10.64 (s, 1H), 7.95 - 7.93 (m, 2H), 7.74 - 7.67 (m, 3H), 7.34 - 7.29 (m, 1H), 7.04 - 7.00 (m, 1H); LC-MS (ESI); [M-H]$^{-}$: 298.0

#GR123994#
SOH-III-91-01

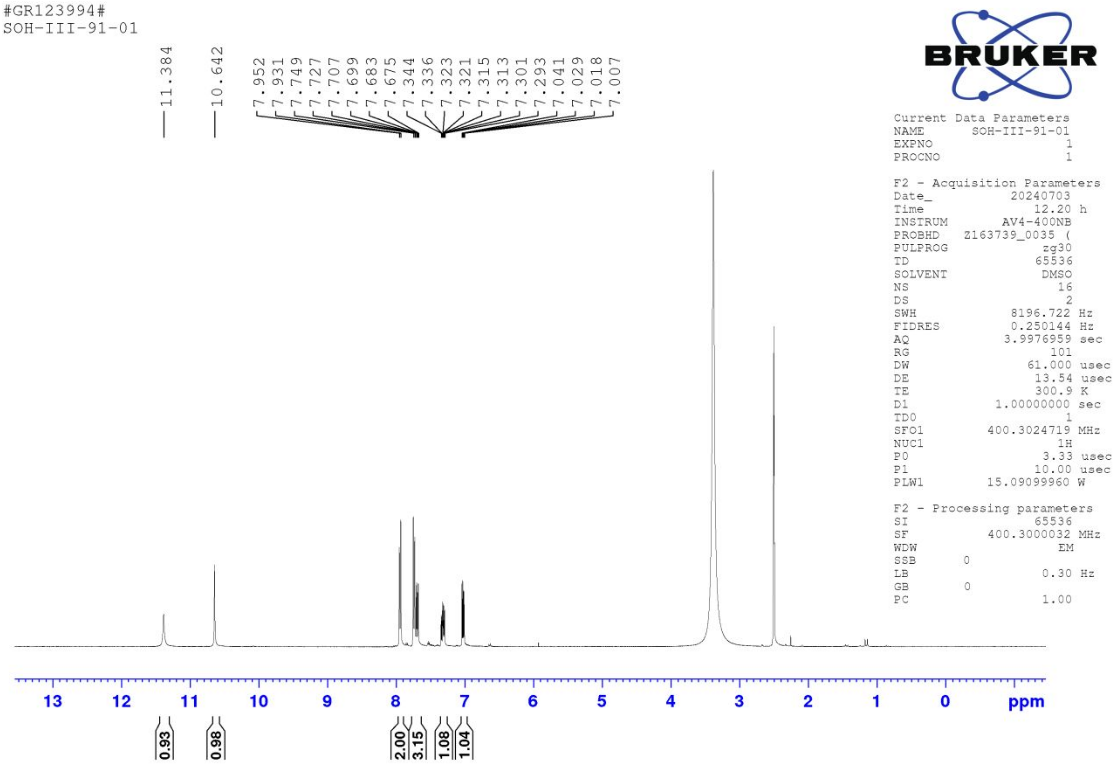

| | | |
|---|---|---|
| Vial:1:42 | ID:mohammed210_162-10-20241106-1404 | File:mohammed210_162-10-20241106-1404_COD |
| Date:06-Nov-2024 | Time:15:00:51 | Description:Nic-60 |
| Method:C:\MassLynx\OpenLynx_Methods\ESI-_100-1250+PDA.olp | | Instrument:ACQ-QDA#KBD6021 |
| MS Method:OA_ESI-_Default | Inlet Method:OA_Default | Detectors:Waters Acquity PDA |

Printed: Wed Nov 06 15:07:02 2024

**Sample Report (continued):**

**Peak ID** 7 **Time** 2.59

7:(Time: 2.59) Combine (446:506-(416:445+507:536)) 1:MS ES- 1.5e+006

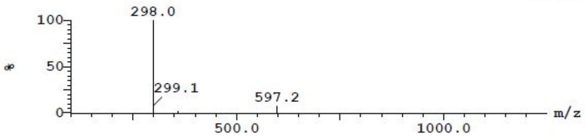


**Peak ID** 7 **Time** 2.59

7:(Time: 2.59) Combine (774) 2:UV Detector 1.46 AU

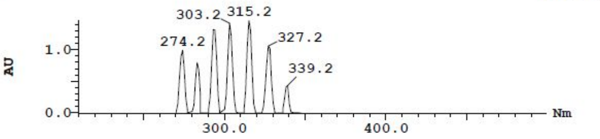


**Peak ID** 8 **Time** 2.63

8:(Time: 2.63) Combine (458:518-(428:457+519:548)) 1:MS ES- 8.3e+005

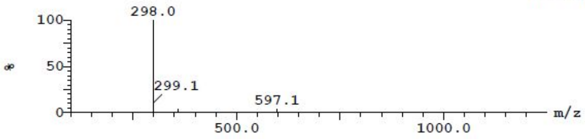


**Peak ID** 9 **Time** 2.66

9:(Time: 2.66) Combine (469:529-(439:468+530:559)) 1:MS ES- 2.9e+004

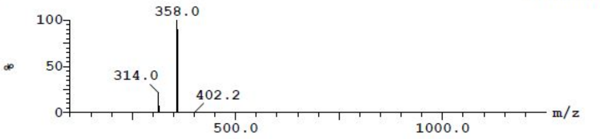


**Peak ID** 10 **Time** 2.69

10:(Time: 2.69) Combine (477:537-(447:476+538:567)) 1:MS ES- 3.0e+004

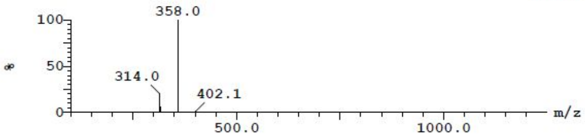


**Peak ID** 10 **Time** 2.69

10:(Time: 2.69) Combine (806) 2:UV Detector 5.528e-1 AU

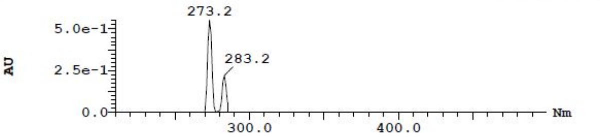

## Reference:


(1) Kam Y, R. G., Winer L, Schwalfenberg M, Romero N. A Customized XF Workflow for Detection and Characterization of Mitochondrial Toxicity. *Agilent Technologies* **2022**. DOI: https://www.agilent.com/cs/library/applications/an-customized-xf-workflow-for-detection-of-mitocondrial-toxicity-5994-4778en-agilent.pdf.

(2) Jiang, H.; Greathouse, R. L.; Tiche, S. J.; Zhao, M.; He, B.; Li, Y.; Li, A. M.; Forgo, B.; Yip, M.; Li, A. Mitochondrial uncoupling induces epigenome remodeling and promotes differentiation in neuroblastoma. *Cancer research* **2023**, *83* (2), 181-194.

(3) Jiang, H.; Tiche, S. J.; He, C. J.; Liu, J.; Bian, F.; Jedoui, M.; Forgo, B.; Islam, M. T.; Zhao, M.; Emengo, P.; et al. Restoring mitochondrial quantity and quality to reverse the Warburg effect and drive neuroblastoma differentiation. *Proceedings of the National Academy of Sciences* **2025**, *122* (36), e2502483122. DOI: doi:10.1073/pnas.2502483122.

(4) Frisch, M.; Trucks, G.; Schlegel, H.; Scuseria, G.; Robb, M.; Cheeseman, J.; Scalmani, G.; Barone, V.; Petersson, G.; Nakatsuji, H. Gaussian 16, revision a. 03, gaussian, inc., wallingford ct. *Gaussian16 (Revision A. 03)* **2016**.

(5) Tetko, I. V.; Gasteiger, J.; Todeschini, R.; Mauri, A.; Livingstone, D.; Ertl, P.; Palyulin, V. A.; Radchenko, E. V.; Zefirov, N. S.; Makarenko, A. S. Virtual computational chemistry laboratory–design and description. *Journal of computer-aided molecular design* **2005**, *19*, 453-463.

(6) Pedretti, A.; Mazzolari, A.; Gervasoni, S.; Fumagalli, L.; Vistoli, G. The VEGA suite of programs: An versatile platform for cheminformatics and drug design projects. *Bioinformatics* **2021**, *37* (8), 1174-1175.

(7) Stewart, J. J. Mopac2016. *Stewart Computational Chemistry: Colorado Springs, CO, USA* **2016**, *650*.

(8) Schrödinger Release 2024-3: LigPrep, Schrödinger, LLC, New York, NY, 2024.

(9) Johnston, R. C.; Yao, K.; Kaplan, Z.; Chelliah, M.; Leswing, K.; Seekins, S.; Watts, S.; Calkins, D.; Chief Elk, J.; Jerome, S. V. Epik: p K a and protonation state prediction through machine learning. *Journal of chemical theory and computation* **2023**, *19* (8), 2380-2388.

(10) Roos, K.; Wu, C.; Damm, W.; Reboul, M.; Stevenson, J. M.; Lu, C.; Dahlgren, M. K.; Mondal, S.; Chen, W.; Wang, L. OPLS3e: Extending force field coverage for drug-like small molecules. *Journal of chemical theory and computation* **2019**, *15* (3), 1863-1874.

(11) Watts, K. S.; Dalal, P.; Tebben, A. J.; Cheney, D. L.; Shelley, J. C. Macrocycle conformational sampling with MacroModel. *Journal of chemical information and modeling* **2014**, *54* (10), 2680-2696.

(12) Mohamadi, F.; Richards, N. G. J.; Guida, W. C.; Liskamp, R.; Lipton, M.; Caufield, C.; Chang, G.; Hendrickson, T.; Still, W. C. Macromodel—an integrated software system for modeling organic and bioorganic molecules using molecular mechanics. *Journal of Computational Chemistry* **1990**, *11* (4), 440-467.

(13) Schrödinger Release 2024-3: MacroModel, Schrödinger, LLC, New York, NY, 2024.

(14) Viayna, A.; Pinheiro, S.; Curutchet, C.; Luque, F. J.; Zamora, W. J. Prediction of n-octanol/water partition coefficients and acidity constants (pK a) in the SAMPL7 blind challenge with the IEFPCM-MST model. *Journal of Computer-Aided Molecular Design* **2021**, *35* (7), 803-811.

(15) Kendall, R. A.; Dunning Jr, T. H.; Harrison, R. J. Electron affinities of the first-row atoms revisited. Systematic basis sets and wave functions. *The Journal of chemical physics* **1992**, *96* (9), 6796-6806.

(16) Hansch, C.; Rockwell, S. D.; Jow, P. Y.; Leo, A.; Steller, E. E. Substituent constants for correlation analysis. *Journal of medicinal chemistry* **1977**, *20* (2), 304-306.

(17) Tribble, M. T.; Traynham, J. G. Nuclear magnetic resonance studies of ortho-substituted phenols in dimethyl sulfoxide solutions. Electronic effects of ortho substituents. *Journal of the American Chemical Society* **1969**, *91* (2), 379-388.

(18) Charton, M. Nature of the ortho effect. V. Ortho-substituent constants. *Journal of the American Chemical Society* **1969**, *91* (24), 6649-6654.

(19) Hanch, C.; Leo, A. Substituent Constants for Correlation Analysis in Chemistry and Biology, Wiley-Interscience. **1979**.

(20) Fabian, P. Scikit-learn: Machine learning in Python. *Journal of machine learning research 12* **2011**, 2825.
(21) Roy, K.; Kar, S.; Ambure, P. On a simple approach for determining applicability domain of QSAR models. *Chemometrics and Intelligent Laboratory Systems* **2015**, *145*, 22-29.
(22) Lal, J.; Ramalingam, K.; Meena, R.; Ansari, S. B.; Saxena, D.; Chopra, S.; Goyal, N.; Reddy, D. N. Design and synthesis of novel halogen rich salicylanilides as potential antileishmanial agents. *European Journal of Medicinal Chemistry* **2023**, *246*, 114996. DOI: https://doi.org/10.1016/j.ejmech.2022.114996.
(23) Mito, S.; Cheng, B.; Garcia, B. A.; Yee Ooi, X.; Gonzalez, D.; Ruiz, T. C.; Elisarraras, F. X.; Tsin, A. SAR study of niclosamide derivatives for neuroprotective function in SH-SY5Y neuroblastoma. *Bioorganic & Medicinal Chemistry Letters* **2023**, *96*, 129498. DOI: https://doi.org/10.1016/j.bmcl.2023.129498.
(24) Mook Jr, R. A.; Wang, J.; Ren, X.-R.; Chen, M.; Spasojevic, I.; Barak, L. S.; Lyerly, H. K.; Chen, W. Structure–activity studies of Wnt/β-catenin inhibition in the Niclosamide chemotype: Identification of derivatives with improved drug exposure. *Bioorganic & medicinal chemistry* **2015**, *23* (17), 5829-5838.
(25) Kang, S.; Min, H.-J.; Kang, M.-S.; Jung, M.-G.; Kim, S. Discovery of novel 2-hydroxydiarylamide derivatives as TMPRSS4 inhibitors. *Bioorganic & Medicinal Chemistry Letters* **2013**, *23* (6), 1748-1751. DOI: https://doi.org/10.1016/j.bmcl.2013.01.055.
(26) Lee, I.-Y.; Gruber, T. D.; Samuels, A.; Yun, M.; Nam, B.; Kang, M.; Crowley, K.; Winterroth, B.; Boshoff, H. I.; Barry, C. E. Structure–activity relationships of antitubercular salicylanilides consistent with disruption of the proton gradient via proton shuttling. *Bioorganic & Medicinal Chemistry* **2013**, *21* (1), 114-126. DOI: https://doi.org/10.1016/j.bmc.2012.10.056.
(27) Krátký, M.; Bősze, S.; Baranyai, Z.; Szabó, I.; Stolaříková, J.; Paraskevopoulos, G.; Vinšová, J. Synthesis and in vitro biological evaluation of 2-(phenylcarbamoyl)phenyl 4-substituted benzoates. *Bioorganic & Medicinal Chemistry* **2015**, *23* (4), 868-875. DOI: https://doi.org/10.1016/j.bmc.2014.12.019.
(28) Shamim, K.; Xu, M.; Hu, X.; Lee, E. M.; Lu, X.; Huang, R.; Shah, P.; Xu, X.; Chen, C. Z.; Shen, M.; et al. Application of niclosamide and analogs as small molecule inhibitors of Zika virus and SARS-CoV-2 infection. *Bioorganic & Medicinal Chemistry Letters* **2021**, *40*, 127906. DOI: https://doi.org/10.1016/j.bmcl.2021.127906.
(29) Li, Y.-R.; Lin, C.-C.; Huang, C.-Y.; Wong, Y.-H.; Hsieh, C.-H.; Wu, H.-W.; Chen, J. J. W.; Wu, Y.-S. Study of the inhibitory effects on TNF-α-induced NF-κB activation of IMD0354 analogs. *Chemical Biology & Drug Design* **2017**, *90* (6), 1307-1311. DOI: https://doi.org/10.1111/cbdd.13032.
(30) Carbone, G.; Burnley, J.; Moses, J. E. A catalytic and tert-butoxide ion-mediated amidation of aldehydes with para-nitro azides. *Chemical Communications* **2013**, *49* (27), 2759-2761.
(31) Krátký, M.; Štěpánková, Š.; Vorčáková, K.; Vinšová, J. Investigation of salicylanilide and 4-chlorophenol-based N-monosubstituted carbamates as potential inhibitors of acetyl- and butyrylcholinesterase. *Bioorganic Chemistry* **2018**, *80*, 668-673. DOI: https://doi.org/10.1016/j.bioorg.2018.07.017.
(32) Chae, H.-D.; Cox, N.; Capolicchio, S.; Lee, J. W.; Horikoshi, N.; Kam, S.; Ng, A. A.; Edwards, J.; Butler, T.-L.; Chan, J.; et al. SAR optimization studies on modified salicylamides as a potential treatment for acute myeloid leukemia through inhibition of the CREB pathway. *Bioorganic & Medicinal Chemistry Letters* **2019**, *29* (16), 2307-2315. DOI: https://doi.org/10.1016/j.bmcl.2019.06.023.
(33) Xu, J.; Berastegui-Cabrera, J.; Chen, H.; Pachón, J.; Zhou, J.; Sánchez-Céspedes, J. Structure–Activity Relationship Studies on Diversified Salicylamide Derivatives as Potent Inhibitors of Human Adenovirus Infection. *Journal of Medicinal Chemistry* **2020**, *63* (6), 3142-3160. DOI: 10.1021/acs.jmedchem.9b01950.
(34) Zhu, Z.-W.; Shi, L.; Ruan, X.-M.; Yang, Y.; Li, H.-Q.; Xu, S.-P.; Zhu, H.-L. Synthesis and antiproliferative activities against Hep-G2 of salicylanide derivatives: potent inhibitors of the epidermal growth factor receptor (EGFR) tyrosine kinase. *Journal of enzyme inhibition and medicinal chemistry* **2011**, *26* (1), 37-45.

(35) Stachulski, A. V.; Pidathala, C.; Row, E. C.; Sharma, R.; Berry, N. G.; Lawrenson, A. S.; Moores, S. L.; Iqbal, M.; Bentley, J.; Allman, S. A. Thiazolides as novel antiviral agents. 2. Inhibition of hepatitis C virus replication. *Journal of medicinal chemistry* **2011**, *54* (24), 8670-8680.
(36) Madasamy, K.; Balakrishnan, M. H.; Korivi, R.; Mannathan, S. Trifluoroacetic Acid-Mediated Denitrogenative ortho-Hydroxylation of 1,2,3-Benzotriazin-4(3H)-ones: A Metal-Free Approach. *The Journal of Organic Chemistry* **2022**, *87* (13), 8752-8756. DOI: 10.1021/acs.joc.2c00354.
(37) Nemeth, A. M.; Basak, A. K.; Weig, A. W.; Marrujo, S. A.; Barker, W. T.; Jania, L. A.; Hendricks, T. A.; Sullivan, A. E.; O'Connor, P. M.; Melander, R. J.; et al. Structure–Function Studies on IMD-0354 Identifies Highly Active Colistin Adjuvants. *ChemMedChem* **2020**, *15* (2), 210-218. DOI: https://doi.org/10.1002/cmdc.201900560.
(38) Tian, M.; Abdelrahman, A.; Baqi, Y.; Fuentes, E.; Azazna, D.; Spanier, C.; Densborn, S.; Hinz, S.; Schmid, R.; Müller, C. E. Discovery and Structure Relationships of Salicylanilide Derivatives as Potent, Non-acidic P2X1 Receptor Antagonists. *Journal of Medicinal Chemistry* **2020**, *63* (11), 6164-6178. DOI: 10.1021/acs.jmedchem.0c00435.
(39) Lu, T.; Zheng, X.; Mao, F.; Cao, Q.; Cao, Q.; Zhu, J.; Li, X.; Lan, L.; Li, B.; Li, J. Novel niclosamide-derived adjuvants elevating the efficacy of polymyxin B against MDR Pseudomonas aeruginosa DK2. *European Journal of Medicinal Chemistry* **2022**, *236*, 114318.
(40) Coomar, S.; Gasser, J. A.; Słabicki, M.; Donovan, K. A.; Fischer, E. S.; Ebert, B. L.; Gillingham, D.; Thomä, N. H. Niclosamide: CRL4 AMBRA1 mediated degradation of cyclin D1 following mitochondrial membrane depolarization. *RSC Medicinal Chemistry* **2025**.
(41) Gundamalla, R.; Bantu, R.; Sridhar, B.; Reddy, B. S. C–H Activation: A Versatile Tool for the Synthesis of Niclosamide and Its Derivatives. *Synlett* **2024**, *35* (20), 2520-2524.
(42) Li, R.; Zhang, Z.; Huang, S.; Peng, K.; Jiang, H.; Shen, J.; Zhang, B.; Jiang, X. Synthesis, cytotoxicity, and pharmacokinetic evaluations of niclosamide analogs for anti-SARS-CoV-2. *European journal of medicinal chemistry* **2023**, *253*, 115320.
